# Comparing the Efficacy and Safety of Cell Transplantation for Spinal Cord Injury: A Systematic Review and Bayesian Network Meta-Analysis

**DOI:** 10.3389/fncel.2022.860131

**Published:** 2022-04-04

**Authors:** Xiongjie Xu, Zeyan Liang, Yike Lin, Jian Rao, Fabin Lin, Zhelun Yang, Rui Wang, Chunmei Chen

**Affiliations:** Department of Neurosurgery, Fujian Medical University Union Hospital, Fuzhou, China

**Keywords:** Bayesian network meta-analysis, cell transplantation, efficacy, spinal cord injury, functional recovery

## Abstract

**Objective:**

To compare the safety and effectiveness of transplanted cells from different sources for spinal cord injury (SCI).

**Design:**

A systematic review and Bayesian network meta-analysis.

**Data Sources:**

Medline, Embase, and the Cochrane Central Register of Controlled Trials.

**Study Selection:**

We included randomized controlled trials, case–control studies, and case series related to cell transplantation for SCI patients, that included at least 1 of the following outcome measures: American Spinal Cord Injury Association (ASIA) Impairment Scale (AIS grade), ASIA motor score, ASIA sensory score, the Functional Independence Measure score (FIM), International Association of Neurorestoratology Spinal Cord Injury Functional Rating Scale (IANR-SCIFRS), or adverse events. Follow-up data were analyzed at 6 and 12 months.

**Results:**

Forty-four eligible trials, involving 1,266 patients, investigated 6 treatments: olfactory ensheathing cells (OECs), neural stem cells/ neural progenitor cells (NSCs), mesenchymal stem cells (MSCs), Schwann cells, macrophages, and combinations of cells (MSCs plus Schwann cells). Macrophages improved the AIS grade at 12 months (mean 0.42, 95% credible interval: 0–0.91, low certainty) and FIM score at 12 months (42.83, 36.33–49.18, very low certainty). MSCs improved the AIS grade at 6 months (0.42, 0.15–0.73, moderate certainty), the motor score at 6 months (4.43, 0.91–7.78, moderate certainty), light touch at 6 (10.01, 5.81–13.88, moderate certainty) and 12 months (11.48, 6.31–16.64, moderate certainty), pinprick score at 6 (14.54, 9.76–19.46, moderate certainty) and 12 months (12.48, 7.09–18.12, moderate certainty), and the IANR-SCIFRS at 6 (3.96, 0.62–6.97, moderate certainty) and 12 months (5.54, 2.45–8.42, moderate certainty). OECs improved the FIM score at 6 months (9.35, 1.71–17.00, moderate certainty). No intervention improved the motor score significantly at 12 months. The certainty of other interventions was low or very low. Overall, the number of adverse events associated with transplanted cells was low.

**Conclusions:**

Patients with SCI who receive transplantation of macrophages, MSCs, NSCs, or OECs may have improved disease prognosis. MSCs are the primary recommendations. Further exploration of the mechanism of cell transplantation in the treatment of SCI, transplantation time window, transplantation methods, and monitoring of the number of transplanted cells and cell survival is needed.

**Systematic Review Registration:**

https://www.crd.york.ac.uk/PROSPERO/#recordDetails, identifier: CRD 42021282043.

## Introduction

Spinal cord injury (SCI) accounts for a relatively large proportion of the global injury burden (Collaborators GTBIaSCI, 2019). In 2016, there were 27.04 million (24.98–30.15 million) SCI cases globally, including 0.93 million (0.78–1.16 million) new cases. Traumatic SCI in China showed an increasing trend from 2009 to 2018, with an estimated 66.5 cases/million population (95% confidence interval, 65.2–67.8) (Hao et al., 2021). SCI can impose an enormous physical, emotional, and financial burden on patients, families, and society (Badhiwala et al., 2019; Hejrati and Fehlings, 2021).

Clinical manifestations of SCI include paralysis, numbness, or loss of bladder or bowel control (Badhiwala et al., 2021). Surgical treatment mainly restores the spinal canal volume by removing bone fragments, ligaments, and hematomas that compress the spinal cord (Wilson et al., 2013). A considerable number of patients do not show neurological improvement after decompression. A study involving 1,548 patients with SCI found that only about 30% of patients with complete paralysis had some improvement in neurological function at 1-year post-surgery, regardless of whether decompression was achieved early (≤ 24 h post-injury) or late (>24 h post-injury) (Badhiwala et al., 2021). Therefore, more effective therapeutic measures are needed.

In addition to external forces and other injury factors acting directly on the spinal cord, leading to SCI, inflammatory reactions, bleeding, and other secondary cascade injuries lead to neuronal and glial cell apoptosis at the injury site. Nerve axonal demyelination can further cause and aggravate SCI (Pukos et al., 2019; Suzuki and Sakai, 2021). During the inflammatory response, secretion of tumor necrosis factor (TNF), interleukins (ILs), and other inflammatory cytokines allows neutrophils, monocytes, and other cells to penetrate the blood–spinal cord barrier into the injury site, coupled with destruction of the blood–spinal cord barrier and glial scar formation. Thus, SCI is gradually aggravated and neurological function deteriorates continuously (Ahuja et al., 2017; Shinozaki et al., 2021).

Neuroprotection and nerve regeneration therapy are important SCI treatments. Currently, nerve regeneration therapy measures used for SCI treatment include pharmacological therapy, cell transplantation, biological scaffolds, and neuromodulatory agents (Hejrati and Fehlings, 2021).

Cell transplantation is the most promising SCI treatment at present, with mechanisms involving immune regulation, neuroprotection, axon regeneration, and myelin regeneration (Ahuja et al., 2017; Assinck et al., 2017; Shinozaki et al., 2021). The most widely studied transplanted cells for SCI treatment include neural stem/neural precursor cells (NSCs), mesenchymal stem cells (MSCs), Schwann cells, olfactory ensheathing cells (OECs), and macrophages, etc. (Bartlett et al., 2020; Hejrati and Fehlings, 2021; Suzuki and Sakai, 2021). Numerous clinical trials have applied cell therapy in SCI treatment (Knoller et al., 2005; Bhanot et al., 2011; Chen et al., 2014; Cheng et al., 2014; Oraee-Yazdani et al., 2016; Anderson et al., 2017; Curt et al., 2020). Yang et al. (2021) and Vaquero et al. (2017) both reported that patients with SCI who received MSC transplantation showed significant neurological recovery during the follow-up period. However, Kishk et al. (2010) found no significant neurological recovery in patients who received MSC transplantation. Knoller et al. (2005) reported that clinical symptoms were improved in 3 patients (3/8) who underwent macrophage transplantation.

Different types of transplanted cells have therefore been reported to be effective in restoring neurological function in SCI patients, but studies have shown that the efficacy of transplanted cells is not uniform. While various transplanted cell types have been used in clinical practice, no clinical studies have compared the results of two or more transplanted cell types, either directly or indirectly, as most of these clinical applications involved phase I or phase II studies (Knoller et al., 2005; Lammertse et al., 2012; Tabakow et al., 2013; Ghobrial et al., 2017; Curtis et al., 2018; Gant et al., 2021). The lack of such information complicates the choice of cell for clinical applications.

Paired meta-analysis to date have mainly analyzed MSCs (Xu and Yang, 2019; Muthu et al., 2021) or OECs (Li et al., 2015), and none have investigated ≥2 different transplanted cell types, leaving the difference in the efficacy of different transplanted cell types in SCI treatment unknown. The single network meta-analysis (NMA) published to date (Chen et al., 2021) compared MSCs from different sources or transplantation routes. Therefore, comparison of different transplanted cell types would be valuable.

Additionally, pairwise meta-analysis can only perform direct pairwise comparisons and cannot compare ≥2. NMA, by combining direct and indirect comparisons, allows comparison of different interventions in the absence of direct two-by-two comparisons, and allows ranking of different interventions by efficacy (Mills et al., 2013), thus making fuller use of the limited data available.

Our study compared the differences in safety and efficacy of transplanted cells from different sources in SCI by means of a systematic review and NMA.

## Methods

### Literature Search and Study Selection

This systematic review and NMA were performed according to Preferred Reporting Items for Systematic Reviews and Meta-Analyses for Network Meta-Analyses (PRISMA-NMA) checklist for network meta-analysis (Hutton et al., 2015) (Supplementary Table S1). The study protocol was registered on PROSPERO (CRD 42021282043).

Three databases, i.e., Medline, Embase, and the Cochrane Central Register of Controlled Trials, were comprehensively searched from their inception to September 2021, without language limitation. We also systematically screened the reference lists of the relevant articles to identify potentially eligible studies (Supplementary Table S2).

The inclusion and exclusion criteria were listed in Table 1. Two trained investigators with extensive retrieval experience separately performed a comprehensive literature search. Any discrepancies were settled through discussion to consensus or were adjudicated by a third investigator.

**Table 1:** The inclusion and exclusion criteria.

**Inclusion criteria:**
Those in which patients with SCI treated with at least 1 of the following cell types: OECs, NSCs, MSCs, Schwann cells, or macrophages.
Those with outcomes of interest: American SCI Association (ASIA) Impairment Scale (AIS grade) (Kirshblum et al., 2011; Committee AaIIS, 2019), ASIA motor score, ASIA sensory score (light touch or pinprick score), the Functional Independence Measure score (FIM) (Dickson and Köhler, 1995), International Association of Neurorestoratology SCI Functional Rating Scale (IANR-SCIFRS) (Huang et al., 2020a), or adverse events.
Randomized controlled trials (RCTs), case–control studies, and case series.
Those in which the cells used had a clear source or were of a well-defined cell type (Dominici et al., 2006; Viswanathan et al., 2019; Hejrati and Fehlings, 2021).
Exclusion criteria:
A life-threatening disease: penetrating injury, comma, ventilator supporting breath, severe cardiopulmonary dysfunction, and so on.
Metabolic bone disease.
Sample size < 3.
Did not involve human patients.
Contained none of the outcome measures from the inclusion criteria.
The follow-up time was <6 months.

### Data Extraction and Outcome Measures

We extracted the author information; publication year; study type; number, age, and sex of subjects; injury time; injury segment; type of transplanted cells; source of transplanted cells, follow-up time; and outcome measures of interest from each eligible article. For case series articles that included patient follow-up, we collected detailed information specific to each patient. We extracted outcome measures for all follow-up periods, but focused on functional recovery at 6 and 12 months after transplantation in patients who underwent cell transplantation.

The AIS grade is an international standard for the classification of neurological function in patients with SCI (Kirshblum et al., 2011; Committee AaIIS, 2019). The AIS grade is used as an important measure in guideline development; hence, we selected it as the primary outcome measure in this study.

The primary outcome, the AIS grade, is classified into 5 grades (A–E; A for complete SCI, and B–E for incomplete SCI, Supplementary Table S3). Secondary outcome measures were the ASIA motor, light touch, and pinprick scores, the FIM score, IANR-SCIFRS score, and adverse events. Overall, the higher the score, the better was the recovery.

Adverse events were mainly discussed as those related to cell transplantation, such as cerebrospinal fluid leakage, and central nervous system infection. Based on the National Cancer Institute Common Terminology Criteria (NCICTC) (NCICTC, 2017) and the descriptions of severe adverse events (SAE) in the included studies (Bhanot et al., 2011; Lammertse et al., 2012; Satti et al., 2016; Curt et al., 2020), we classified events requiring hospitalization or resulting in prolonged hospitalization, events requiring urgent intervention, and life-threatening events as SAE (NCICTC grade 3 and 4). Events leading to the occurrence of death are categorized as very severe adverse events (NCICTC grade 5). The remaining events were classified as mild-moderate adverse events (NCICTC grade 1 and 2). And the safety was compared among transplant cells by counting the number of adverse events per capita.

Data extraction was performed separately by 2 investigators. Any discrepancies were resolved by negotiation or by a third investigator. For outcome measures that were only represented on graphs, we extracted data using the GetData Graph Digitizer (version 2.24). For missing values, we would contact the corresponding author to obtain information.

### Risk-of-Bias Assessment

The risk-of-bias was assessed by 2 trained investigators using the Joanna Briggs Institute (JBI) manual for evidence synthesis (Moola et al., 2020; Tufanaru et al., 2020). Investigators resolved any disagreements by discussion, or adjudication by a third investigator.

### Data Synthesis and Statistical Analysis

In data synthesis, the mean difference and 95% credible interval (CI) were used to express the effect value of continuous variables. For case series, in which the overall sample means and standard deviations were not provided, we used SPSS 20.0 to calculate the collected individual data and presented the results as means and standard deviations. Data analysis could be performed approximately as for a continuous variable by converting AIS grade grades A–D to 1–4.

Network plots were constructed separately for each outcome measure. In constructing these plots, we included the baseline status of the recipient cells before transplantation in the same group for synthesis and analysis, and compared it with other transplanted cells to construct the network plots.

We use the Bayesian framework based on Markov chain Monte-Carlo simulation methods to conduct NMA under the random-effects model for the chosen outcome measures (Lumley, 2002; Ades et al., 2006). Fifty thousand iterations were generated with 20 000 burn-ins and a thinning interval of 1 for continuous variables. The trace plots and density plots were used to assess aggregation. The league table was used to present the direct comparison of different interventions. Calculating the surface under the cumulative ranking (SUCRA), was used to rank the therapeutic effects of transplanted cells from different sources for SCI (Salanti et al., 2011). The SUCRA value ranges between 0 and 100%: the larger the value, the more prominent is the ranking.

### Assessing Evidence Certainty

We used the Grade of Recommendations Assessment, Development, and Evaluation (GRADE) for NMA to evaluate evidence quality (Brignardello-Petersen et al., 2018, 2020a,b). Two experienced investigators separately assessed evidence certainty using a minimally contextualized framework. The baseline was chosen as reference intervention and a null effect was set as the decision threshold. According to whether the 95% CI between 2 interventions crossed the threshold, we classified category 0, representing that the intervention's 95% CI contained the decision threshold; otherwise, the intervention was included in category 1. An intervention would be classified as category 2 if it was significantly superior to any of the other interventions. Based on the risk-of-bias, inconsistency, publication bias, and transitivity, 2 main groups were identified: the high-certainty group or low-certainty group. The level of certainty of the evidence for different interventions was rated as high, moderate, low, or very low.

### Hypothesis Test of NMA: Homogeneity, Transitivity, and Consistency Test

For the homogeneity test and transitivity test, heterogeneity was first assessed by evaluating the clinical, methodological, and statistical data of the included studies, followed by meta-regression to explore the source of heterogeneity and assess the homogeneity and transferability.

For the consistency test, a node-split test was performed to explore local inconsistency (van Valkenhoef et al., 2016). The deviance information criterion (DIC) values, calculated by comparing the consistency and inconsistency models under the random-effect model, were compared, with smaller DIC values or a smaller difference in DIC values between 2 models indicating good consistency (Spiegelhalter et al., 2002). A *p* < 0.05 was considered to indicate a statistically significant difference.

### Small-Sample Effect Analysis and Meta-Regression Analysis

Sensitivity analysis was applied to explore whether there were small-sample effects for different outcome measures, by using funnel plots of mean differences between the endpoint and baseline. Statistical analyses were performed to explore the treatment effect of transplanted cells by excluding articles that led to bias, as well as to assess the robustness of the statistical results.

A network meta-regression analysis was performed with selected covariates, such as publication year, baseline, and transplanted cell type. The BETA value and its 95% CI were obtained from the regression analysis. If the 95% CI contained 0, it indicated that the difference was not statistically significant; that is, the covariate had no significant effect on the results.

Each step of NMA (e.g., retrieval, screening, data extraction, data analysis, etc.) was separately performed by two investigators with experience in this step to ensure the highest quality of results as possible. In addition, the investigators all had clinical backgrounds and had previously participated in a meta-analysis.

OpenBugs (version 3.2.3; https://openbugs.net/w/OpenBUGS_3_2_3?action=AttachFile-do=get-target=OpenBUGS-3.2.3.tar.gz) and Rstudio (version 4.1.1 gemtc package; https://www.rstudio.com/) software were used for Bayesian NMA.

For pairwise meta-analysis, we used Rstudio (version 4.1.1 meta and metafor package) to obtain results of direct comparisons, and to compare these with the results of NMA, to explore the robustness of the NMA results.

## Results

**Figure 1:**
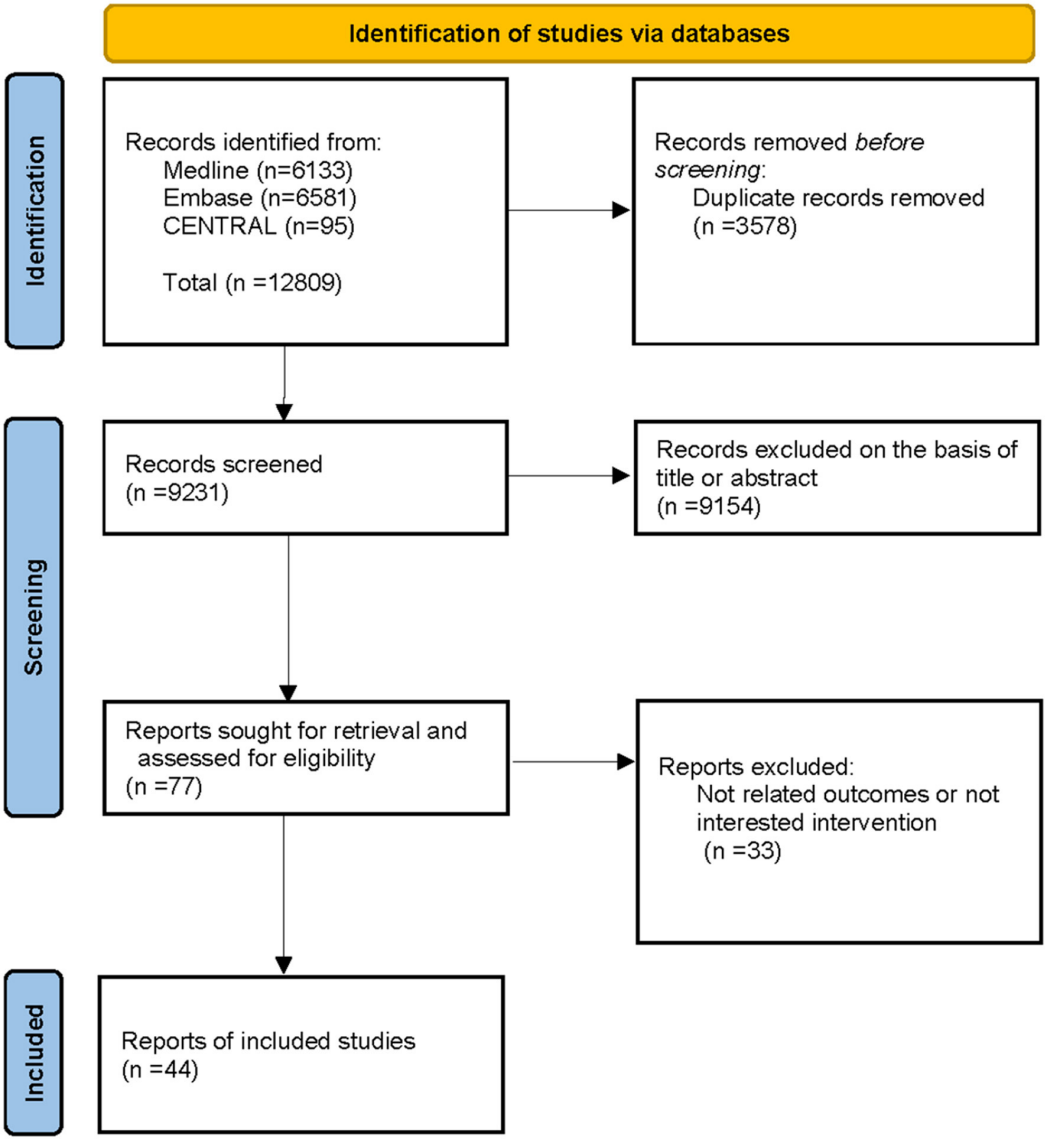
Flowchart of the study selection.

We retrieved 12,809 studies through database searches. After first removing duplicate articles and then conducting initial screening of titles and abstracts and performing full-text screening based on inclusion and exclusion criteria, we identified 43 trials reported in 44 articles and involving 1,266 patients for inclusion in this study (Figure 1). No article on patients with SCI received embryonic stem cells and induced pluripotent stem cells to meet the inclusion and exclusion criteria.

### Study Characteristics and Risk-of-Bias Assessment

Supplementary Table S4 shows the specific information of the 44 included articles. The duration of SCI ranged from <7 days to 360 months. The mean age of patients ranged from 6 to 66 years. Cells were extracted from autologous tissue in 29 articles and were allogeneic in 14 articles. Of the included articles, 5 were RCTs, 8 were case–control, and 30 were case series, with single-arm studies accounting for 70% of the included studies.

Supplementary Table S5 shows the risk-of-bias for all included studies. For case series studies, since most of the studies did not include sample size requirements in the text or on Clinicaltrial.gov, it is unclear whether these were full case numbers.

### Network Meta-Analysis

**Figure 2:**
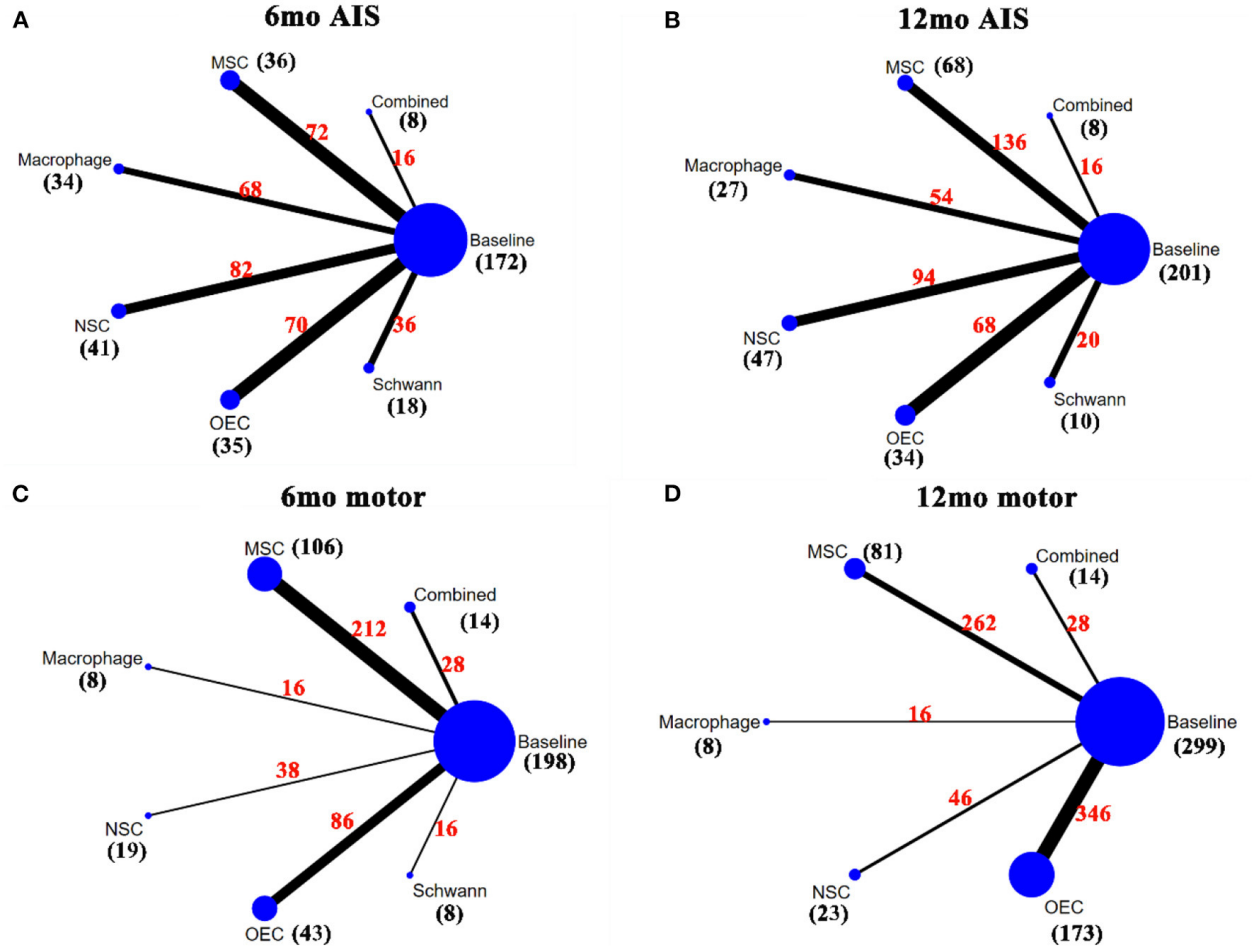
Network plots for AIS grade and motor. **(A)** A network plot for 6mo AIS grade; **(B)** A network plot for 12mo AIS grade; **(C)** A network plot for 6mo ASIA motor score; **(D)** A network plot for 12mo ASIA motor score. The nodes represent different interventions, the size of the nodes represents the sample size of that intervention, and the thickness of the lines connecting the nodes represents the sample size involving both interventions. The black number represents the sample size of the intervention, and the red number represents the total sample size of the two connected interventions. AIS grade, American Spinal Cord Injury Association (ASIA) Impairment Scale; mo, month; MSC, mesenchymal stem cell; NSC, neural stem cell/neural progenitor cell; OEC, olfactory ensheathing cell.

**Figure 3:**
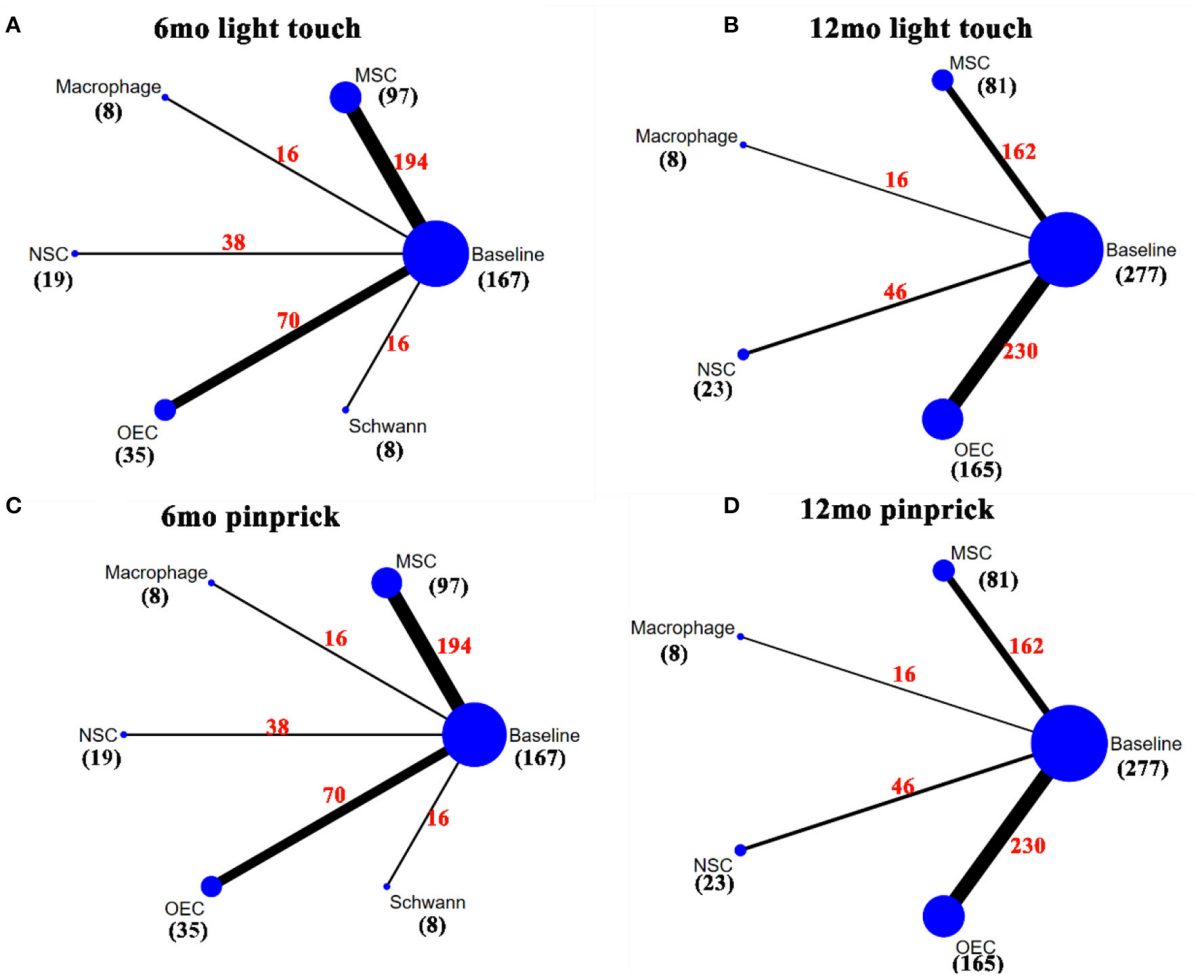
Network plot for light touch and pinprick. **(A)** A network plot for 6mo ASIA light touch score; **(B)** A network plot for 12mo ASIA light touch score; **(C)** A network plot for 6mo ASIA pinprick score; **(D)** A network plot for 12mo ASIA pinprick score. The nodes represent different interventions, the size of the nodes represents the sample size of that intervention, and the thickness of the lines connecting the nodes represents the sample size involving both interventions. The black number represents the sample size of the intervention, and the red number represents the total sample size of the two connected interventions. ASIA, American Spinal Cord Injury Association; mo, month; MSC, mesenchymal stem cell; NSC, neural stem cell/neural progenitor cell; OEC, olfactory ensheathing cell.

**Figure 4:**
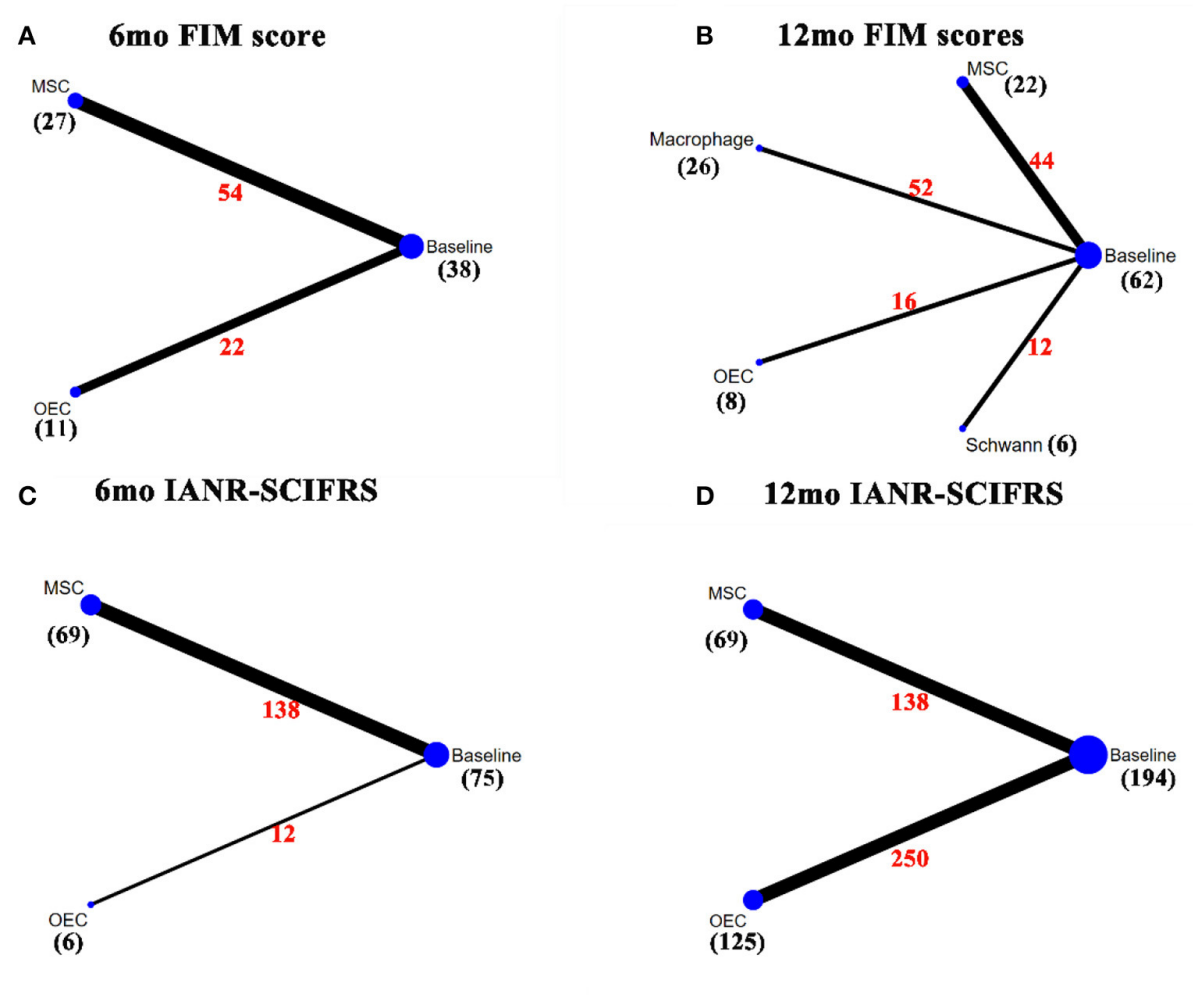
Network plot for FIM score and IANR-SCIFRS. **(A)** A network plot for 6mo FIM score; **(B)** A network plot for 12mo FIM score; **(C)** A network plot for 6mo IANR-SCIFRS; **(D)** A network plot for 12mo IANAR-SCIFRS. The nodes represent different interventions, the size of the nodes represents the sample size of that intervention, and the thickness of the lines connecting the nodes represents the sample size involving both interventions. The black number represents the sample size of the intervention, and the red number represents the total sample size of the two connected interventions. FIM, Functional independence measure; IANR-SCIFRS, International Association of Neurorestoratology Spinal Cord Injury Functional Rating Scale; mo, month; MSC, mesenchymal stem cell; NSC, neural stem cell/neural progenitor cell; OEC, olfactory ensheathing cell.

Figures 2–4 show the network plots for the AIS grade, ASIA motor score, ASIA light touch, ASIA pinprick, FIM score, and IANR-SCIFRS at 6 and 12 months, respectively.

### Primary Outcome: AIS Grade

**Figure 5:**
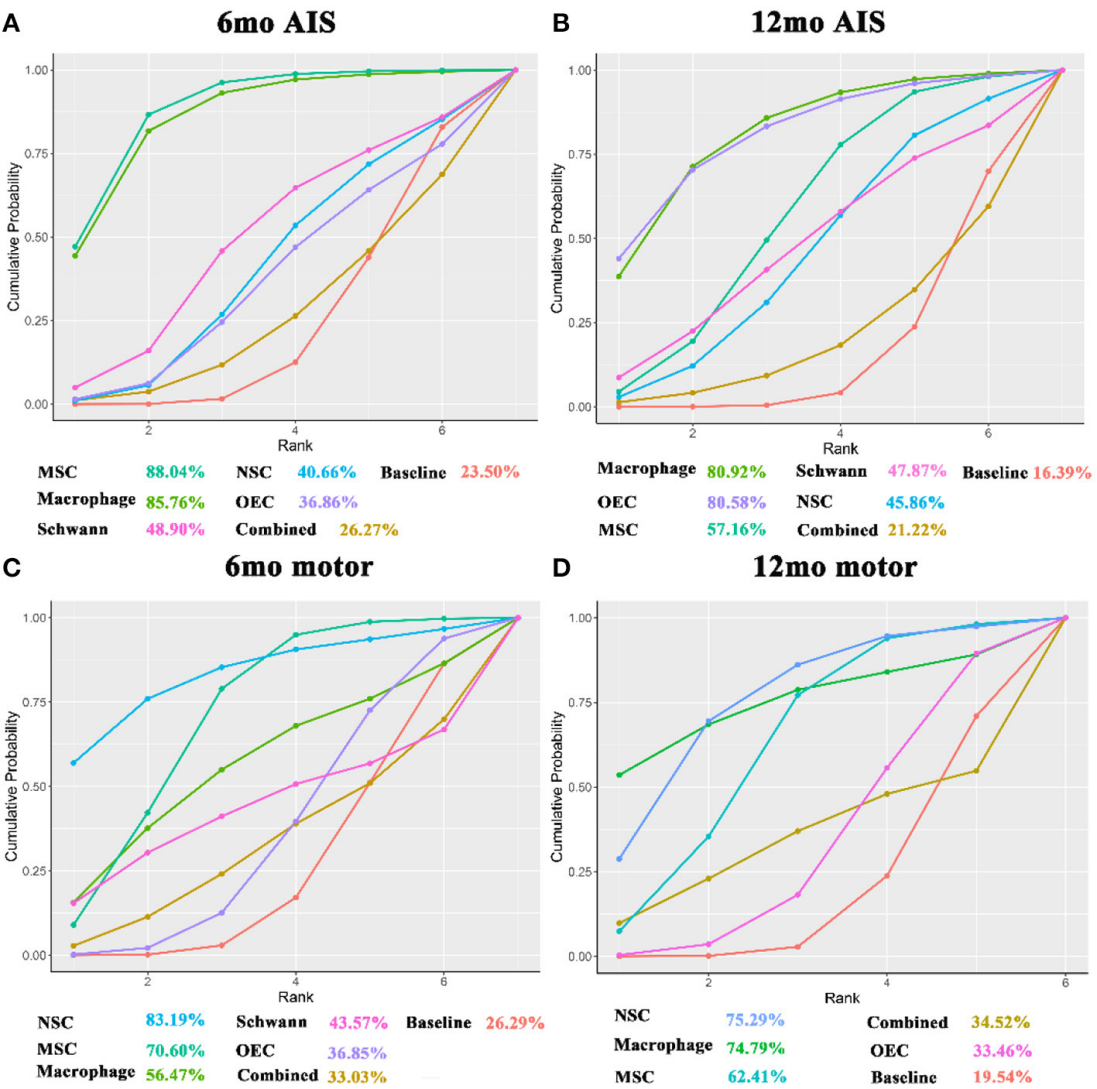
SUCRA for AIS grade and ASIA motor score. **(A)** SUCRA for 6mo AIS grade; **(B)** SUCRA for 12mo AIS grade; **(C)** SUCRA for 6mo ASIA motor score; **(D)** A network plot for 12mo ASIA motor score. The x-axis represents the ranking and the y-axis represents the cumulative probability. The cumulative ranking and cumulative probability are constructed as a curve. The larger the area under the curve, the greater the benefit of the intervention. AIS grade, American Spinal Cord Injury Association (ASIA) Impairment Scale; mo, month; SUCRA, the surface under the cumulative ranking; MSC, mesenchymal stem cell; NSC, neural stem cell/neural progenitor cell; OEC, olfactory ensheathing cell.

For the outcome measure of AIS grade at 6 months (6mo AIS grade), there were 17 articles (Knoller et al., 2005; Saberi et al., 2008; Chhabra et al., 2009; Lima et al., 2010; Lammertse et al., 2012; Saito et al., 2012; Rao et al., 2013a; Tabakow et al., 2013; Yazdani et al., 2013; Mendonça et al., 2014; Shin et al., 2015; Vaquero et al., 2016; Anderson et al., 2017; Larocca et al., 2017; Levi et al., 2019; Curt et al., 2020; Gant et al., 2021), which included 172 patients. After sensitivity analysis, NMA results showed that macrophages (mean 0.41, 95% CI 0.06– 0.8) and MSCs (0.42, 0.15–0.73) significantly improved the 6mo AIS grade after transplantation, as compared to baseline. The remaining transplanted cell types tended to produce results better than baseline, but the differences did not reach statistical significance. No statistically significant differences were observed between the 2 cell types (Table 2A). Figure 5A showed the SUCRA for the different transplanted cell types as compared to baseline according to the 6mo AIS grade. MSCs (88.04%) ranked first, followed by macrophages (85.76%), Schwann cells (48.90%), NSCs (40.66%), and OECs (36.86%), while combined transplantation (26.27%) ranked last.

**Table 2:** Pooled estimates of the network meta8-analysis for the outcome “6mo and 12mo AIS grade” (A), “6mo and 12mo ASIA motor score” (B), “6mo and 12mo ASIA light touch score” (C), “6mo and 12mo ASIA pinprick score” (D), “6mo and 12mo FIM score” (E), and “6mo and 12mo IANR-SCIFRS” (F).

**(A)**	**6mo AIS grade**						
**12mo AIS grade**	Baseline	0 (−0.39, 0.38)	**0.41 (0.06, 0.8)**	**0.42 (0.15, 0.73)**	0.08 (−0.22, 0.38)	0.06 (−0.25, 0.41)	0.13 (−0.29, 0.54)
	0 (−0.49, 0.49)	Combined	0.41 (−0.09, 0.96)	0.42 (−0.03, 0.91)	0.08 (−0.4, 0.56)	0.06 (−0.41, 0.59)	0.13 (−0.43, 0.67)
	**0.42 (0, 0.91)**	0.42 (−0.2, 1.13)	Macrophage	0.01 (−0.46, 0.47)	−0.34 (−0.82, 0.12)	−0.36 (−0.84, 0.14)	−0.28 (−0.87, 0.25)
	0.18 (−0.15, 0.56)	0.18 (−0.4, 0.81)	−0.24 (−0.82, 0.32)	MSC	−0.35 (−0.77, 0.05)	−0.37 (−0.79, 0.07)	−0.29 (−0.81, 0.2)
	0.15 (−0.17, 0.49)	0.15 (−0.43, 0.75)	−0.27 (−0.85, 0.27)	−0.03 (−0.52, 0.43)	NSC	−0.02 (−0.45, 0.44)	0.06 (−0.46, 0.56)
	0.44 (−0.04, 1.01)	0.44 (−0.21, 1.22)	0.02 (−0.64, 0.72)	0.26 (−0.33, 0.91)	0.29 (−0.29, 0.95)	OEC	0.08 (−0.48, 0.58)
	0.18 (−0.35, 0.72)	0.18 (−0.55, 0.9)	−0.25 (−0.98, 0.43)	−0.01 (−0.66, 0.62)	0.02 (−0.61, 0.66)	−0.27 (−1.05, 0.44)	Schwann
**(B)**	**6mo motor**						
**12mo motor**	Baseline	0.09 (−8.1, 8.92)	3.16 (−7.29, 13.53)	**4.43 (0.91, 7.78)**	9.12 (−2.61, 20.91)	0.58 (−1.53, 3.3)	1.13 (−13.87, 16.17)
	0.16 (−8.11, 8.05)	Combined	3.07 (−11.26, 16.6)	4.34 (−5.27, 13.25)	9.04 (−5.9, 23.53)	0.49 (−8.63, 9.12)	1.05 (−16.36, 18.34)
	6.02 (−5.09, 16.57)	5.86 (−7.9, 19.89)	Macrophage	1.27 (−9.77, 12.21)	5.97 (−9.78, 21.42)	−2.58 (−13.11, 8.24)	−2.03 (−20.05, 16.53)
	2.37 (−0.7, 5.25)	2.21 (−6.19, 11.07)	−3.65 (−14.49, 7.97)	MSC	4.69 (−7.58, 16.81)	−3.85 (−7.7, 0.74)	−3.3 (−18.74, 12.16)
	4.17 (−0.68, 9.46)	4.01 (−5.54, 14.22)	−1.85 (−13.3, 10.81)	1.8 (−3.78, 7.82)	NSC	−8.54 (−20.57, 3.55)	−7.99 (−26.48, 10.77)
	0.62 (−0.48, 2.74)	0.46 (−7.48, 9.24)	−5.4 (−15.92, 6.08)	−1.75 (−4.83, 1.95)	−3.55 (−8.8, 1.73)	OEC	0.55 (−14.61, 15.76)
	NA	NA	NA	NA	NA	NA	Schwann
**(C)**	**6mo light touch**						
**12mo light touch**	Baseline	**13.07 (1.03, 25.25)**	**10.01 (5.81, 13.88)**	5.51 (−7.53, 18.8)	2.03 (−4.83, 8.96)	0.11 (−24.88, 24.09)	
	**14.35 (1.02, 27.98)**	Macrophage	−3.06 (−15.88, 9.54)	−7.56 (−25.26, 10.47)	−11.04 (−25.1, 2.86)	−12.97 (−40.6, 14.32)	
	**11.48 (6.31, 16.64)**	−2.87 (−17.4, 11.45)	MSC	−4.51 (−17.98, 9.45)	−7.98 (−15.79, 0.12)	−9.91 (−34.93, 14.35)	
	7.77 (−6.31, 21.79)	−6.58 (−26.24, 12.63)	−3.71 (−18.84, 11.29)	NSC	−3.47 (−18.69, 10.94)	−5.4 (−33.84, 22.47)	
	3.71 (−0.4, 8.24)	−10.64 (−24.76, 3.62)	**−7.77 (−14.27**, **−0.86)**	−4.06 (−18.72, 10.79)	OEC	−1.93 (−27.12, 22.49)	
	NA	NA	NA	NA	NA	Schwann	
**(D)**	**6mo pinprick**						
**12mo pinprick**	Baseline	**11.68 (0.68, 22.92)**	**14.54 (9.76, 19.46)**	3.07 (−4.58, 10.72)	3.13 (−4.16, 10.54)	1.66 (−23.73, 25.95)	
	**16.1 (2.33, 30.17)**	Macrophage	2.87 (−9.36, 15.01)	−8.6 (−22.06, 4.74)	−8.55 (−21.81, 4.98)	−10.02 (−37.96, 16.24)	
	**12.48 (7.09, 18.12)**	−3.62 (−18.94, 11.38)	MSC	**−11.47 (−20.44**, **−2.35)**	**−11.41 (−20.2**, **−2.62)**	−12.89 (−38.66, 11.93)	
	7.2 (−1.22, 15.89)	−8.9 (−25.16, 7.33)	−5.28 (−15.59, 5.06)	NSC	0.06 (−10.5, 10.58)	−1.42 (−27.36, 24.11)	
	3.01 (−0.45, 7.03)	−13.09 (−27.54, 1.21)	**−9.47 (−15.98**, **−2.75)**	−4.19 (−13.38, 5.33)	OEC	−1.47 (−27.23, 23.96)	
	NA	NA	NA	NA	NA	Schwann	
**(E)**	**6mo FIM score**						
**12mo FIM score**	Baseline	NA	2.81 (−2.89, 8.66)	**9.35 (1.71, 17)**	NA		
	**42.83 (36.33, 49.18)**	Macrophage	NA	NA	NA		
	1.26 (**–**4.77, 7.48)	**−41.57 (−50.31**, **−32.54)**	MSC	6.54 (−3.08, 16)	NA		
	**21.02 (9.75, 32.2)**	**−21.81 (−34.54**, **−9.04)**	**19.76 (7.17, 32.22)**	OEC	NA		
	**34.52 (14.89, 54.23)**	−8.31 (−28.74, 12.28)	**33.26 (12.95, 55.86)**	13.5 (−8.76, 36.23)	Schwann		
**(F)**	**6mo IANR-SCIFRS**						
**12mo IANR-SCIFRS**	Baseline	**3.96 (0.62, 6.97)**	6.28 (−5.26, 17.67)				
	**5.54 (2.45, 8.42)**	MSC	2.33 (−9.51, 14.13)				
	2.16 (−0.24, 5.22)	−3.38 (−7.03, 1.12)	OEC				

*The pooled effect sizes are expressed as mean differences and 95% CIs. An area of upper or lower triangles indicates a particular outcome (e.g., in **(A)**, the upper triangle indicates 6mo AIS grade; the lower triangle indicates 12mo AIS grade). Comparisons between interventions should be read from left to right, with the cells sandwiched between the interventions defined in rows and those defined in columns being the effect values for both. In the upper triangle, mean differences higher than 0 favor the interventions defined by columns, and in the lower triangle, mean differences higher than 0 favor interventions defined by rows*.

*AIS grade, American Spinal Cord Injury Association (ASIA) Impairment Scale; CIs, credible intervals; FIM, Functional independence measure; IANR-SCIFRS, International Association of Neurorestoratology Spinal Cord Injury Functional Rating Scale; mo, month; MSC, mesenchymal stem cell; NA, not applicable, no articles for comparison; NSC, neural stem cell/neural progenitor cell; OEC, olfactory ensheathing cell. The different colors are used to better distinguish the effect values of the different outcome indicators. The blue areas represent the different interventions. In Table 2A, pink represents the 6mo AIS grade and gray represents the 12mo AIS grade. the same representation also appears in Tables 2B–F*.

For the AIS grade at 12 months (12mo AIS grade), 16 articles (Knoller et al., 2005; Saberi et al., 2008; Chhabra et al., 2009; Kishk et al., 2010; Lima et al., 2010; Bhanot et al., 2011; Lammertse et al., 2012; Rao et al., 2013a; Tabakow et al., 2013; Yazdani et al., 2013; Shin et al., 2015; Vaquero et al., 2016; Anderson et al., 2017; Ghobrial et al., 2017; Levi et al., 2019; Curt et al., 2020), including 194 patients, were analyzed. After sensitivity analysis, results showed that the transplanted cell types tended to produce results better than at baseline (p > 0.05), except for macrophages, which yielded a statistically significantly different effect as compared to baseline (0.42, 0–0.91). No statistically significant differences were observed between the 2 cell types (Table 2A). Figure 5B showed SUCRA for the 12mo AIS grade: macrophages (80.92%) ranked first, followed by OECs (80.58%), MSCs (57.16%), Schwann cells (47.87%), and NSCs (45.86%), while combined transplantation (21.22%) ranked last.

### ASIA Motor Score

In terms of the ASIA motor score at 6 months (6mo motor score), there were 17 articles (Knoller et al., 2005; Lima et al., 2006, 2010; Mackay-Sim et al., 2008; Chhabra et al., 2009; Saito et al., 2012; Yazdani et al., 2013; Cheng et al., 2014; Mendonça et al., 2014; Shin et al., 2015; Hur et al., 2016; Iwatsuki et al., 2016; Oraee-Yazdani et al., 2016; Vaquero et al., 2016, 2017; Gant et al., 2021; Yang et al., 2021) including 198 patients. Eighteen articles (Knoller et al., 2005; Lima et al., 2006, 2010; Mackay-Sim et al., 2008; Chhabra et al., 2009; Kishk et al., 2010; Huang et al., 2012; Wu et al., 2012; Yazdani et al., 2013; Chen et al., 2014; Shin et al., 2015; Iwatsuki et al., 2016; Oraee-Yazdani et al., 2016; Vaquero et al., 2016, 2017; Wang et al., 2016; Ghobrial et al., 2017; Yang et al., 2021), reflecting 299 patients, contained the ASIA motor score at 12 months (12mo motor score). Combined with motor scores at 6 and 12 months, MSCs significantly increased motor scores at 6 months after transplantation (4.43, 0.91–7.78 after sensitivity analysis). The remaining transplanted cell types had a trend toward superiority over baseline, but differences were not statistically significant. No statistically significant differences were observed between the 2 cell types (Table 2B). According to the SUCRA results (Figures 5C,D), NSCs ranked first (6 months: 83.19%; 12 months: 75.29%), while combined transplantation (6 months: 33.03%) and OECs (12 months: 33.46%) ranked last.

### ASIA Light Touch Score

**Figure 6:**
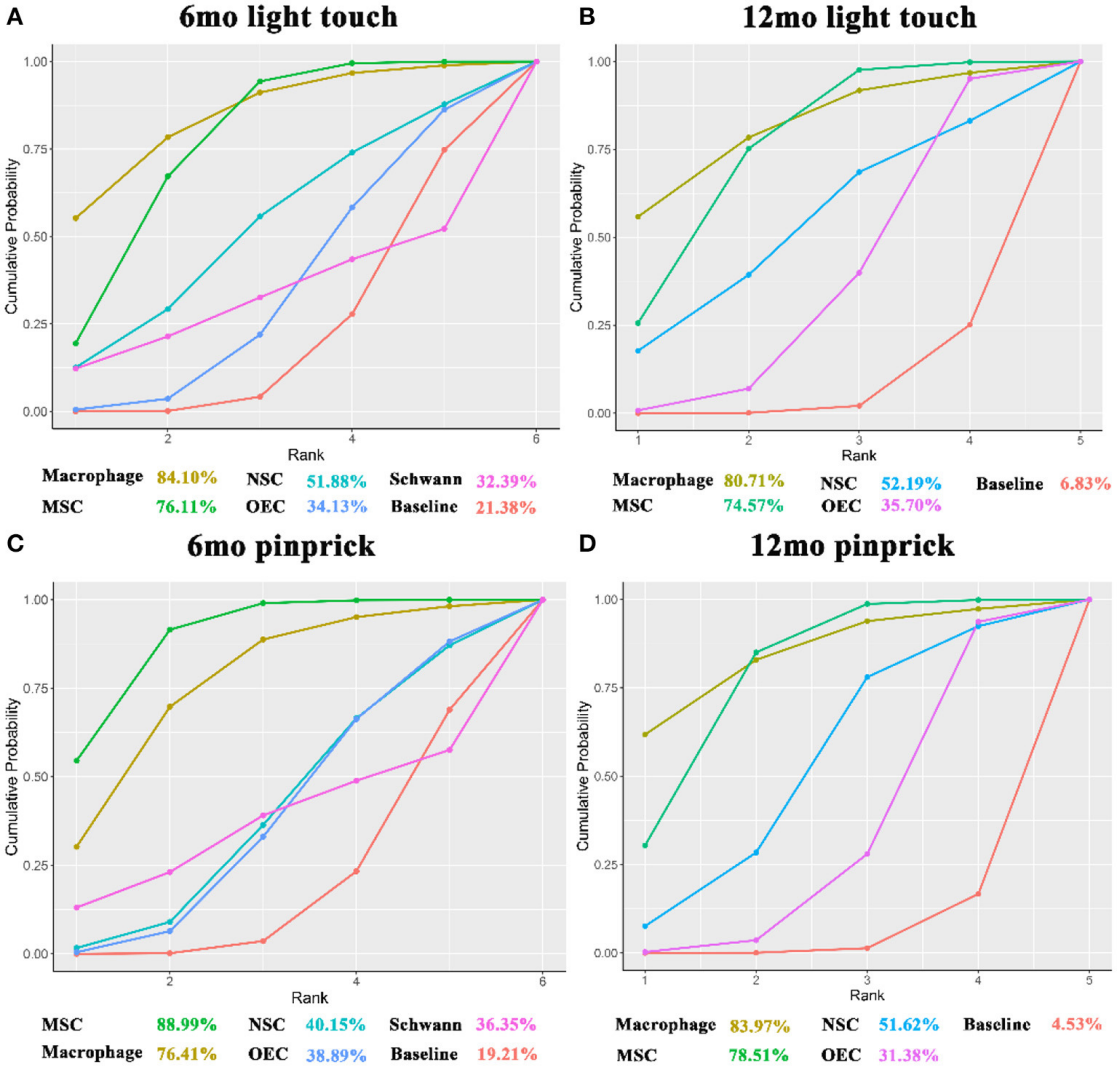
SUCRA for ASIA light touch and pinprick. **(A)** SUCRA for 6mo ASIA light touch score; **(B)** SUCRA for 12mo ASIA light touch score; **(C)** SUCRA for 6mo ASIA pinprick score; **(D)** SUCRA for 12mo ASIA pinprick score. The x-axis represents the ranking and the y-axis represents the cumulative probability. The cumulative ranking and cumulative probability are constructed as a curve. The larger the area under the curve, the greater the benefit of the intervention. mo, month; SUCRA, the surface under the cumulative ranking; MSC, mesenchymal stem cell; NSC, neural stem cell/neural progenitor cell; OEC, olfactory ensheathing cell.

Thirteen articles (Knoller et al., 2005; Lima et al., 2006, 2010; Mackay-Sim et al., 2008; Chhabra et al., 2009; Mendonça et al., 2014; Shin et al., 2015; Hur et al., 2016; Vaquero et al., 2016, 2017, 2018; Gant et al., 2021), involving 167 patients, used the ASIA light touch score at 6 months (6mo light touch score) as outcome measure. Macrophages (13.07, 1.03–25.25) and MSCs (10.01, 5.81–13.88) significantly improved light touch scores after transplantation as compared with baseline (Table 2C). According to SUCRA (Figure 6A), macrophages ranked first (84.10%), followed by MSCs (76.11%), NSCs (51.88%), OECs (34.13%), and Schwann cells (32.39%).

Fifteen articles (Knoller et al., 2005; Lima et al., 2006, 2010; Mackay-Sim et al., 2008; Chhabra et al., 2009; Kishk et al., 2010; Huang et al., 2012; Wu et al., 2012; Chen et al., 2014; Shin et al., 2015; Vaquero et al., 2016, 2017; Wang et al., 2016; Ghobrial et al., 2017; Yang et al., 2021), involving 277 patients, included the ASIA light touch score at 12 months (12mo light touch score) as outcome measure. Transplantation of macrophages (14.35, 1.02–27.98) and MSCs (11.48, 6.31–16.64) significantly improved light touch scores. Compared with OECs, MSCs (−7.77, −14.27 to −0.86) significantly increased the 12mo light touch score (Table 2C). According to SUCRA (Figure 6B), macrophages ranked first (80.71%), followed by MSCs (74.57%), NSCs (52.19%), and OECs (35.70%).

### ASIA Pinprick Score

For analysis of the ASIA pinprick score at 6 months (6mo pinprick score), 13 articles (Knoller et al., 2005; Lima et al., 2006, 2010; Mackay-Sim et al., 2008; Chhabra et al., 2009; Mendonça et al., 2014; Shin et al., 2015; Hur et al., 2016; Vaquero et al., 2016, 2017, 2018; Gant et al., 2021; Yang et al., 2021), involving 167 patients, were included. After sensitivity analysis, macrophages (11.68, 0.68–22.92) and MSCs (14.54, 9.76–19.46) were found to increase the 6mo pinprick score significantly after transplantation. Compared with NSCs (−11.47, −20.44 to −2.35) and OECs (−11.41, −20.2 to −2.62), MSCs significantly increased the 6mo pinprick score (Table 2D). By SUCRA (Figure 6C), MSCs (88.99%) ranked first, followed by macrophages (76.41%), NSCs (40.15%), OECs (38.89%), and Schwann cells (36.35%).

For the ASIA pinprick score at 12 months (12mo pinprick score), 15 articles (Knoller et al., 2005; Lima et al., 2006, 2010; Mackay-Sim et al., 2008; Chhabra et al., 2009; Kishk et al., 2010; Huang et al., 2012; Wu et al., 2012; Chen et al., 2014; Shin et al., 2015; Vaquero et al., 2016, 2017; Wang et al., 2016; Ghobrial et al., 2017; Yang et al., 2021), involving 277 patients, were included. Macrophages (16.1, 2.33–30.17) and MSCs (12.48, 7.09–18.12) significantly improved 12mo pinprick score. MSCs (−9.47, −15.98 to −2.75) significantly improved this score as compared with OECs (Table 2D). According to SUCRA (Figure 6D), macrophages (83.97%) ranked first, followed by MSCs (78.51%), NSCs (51.62%), and OECs (31.38%).

### FIM Score

In the FIM score [including 6 months (Rao et al., 2013a; Tabakow et al., 2013; Vaquero et al., 2016, 2017; Larocca et al., 2017) and 12 months (Lammertse et al., 2012; Rao et al., 2013a; Vaquero et al., 2016, 2017; Anderson et al., 2017)], OECs (6 months: 9.35, 1.71–17.00; 12 months: 21.02, 9.75–32.2), macrophages (12 months: 42.83, 36.33–49.18), and Schwann cells (12 months: 34.52, 14.89–54.23) significantly improved the FIM score after transplantation, as compared with before transplantation. Macrophages, as compared with OECs (−21.81, −34.54 to −9.04); OECs, as compared with MSCs (19.76, 7.17–32.22); macrophages, as compared with MSCs (−41.57, −50.31 to −32.54); and Schwann cells, as compared with MSCs (33.26, 12.95–55.86), significantly improved FIM scores at 12 months (Table 2E).

**Figure 7:**
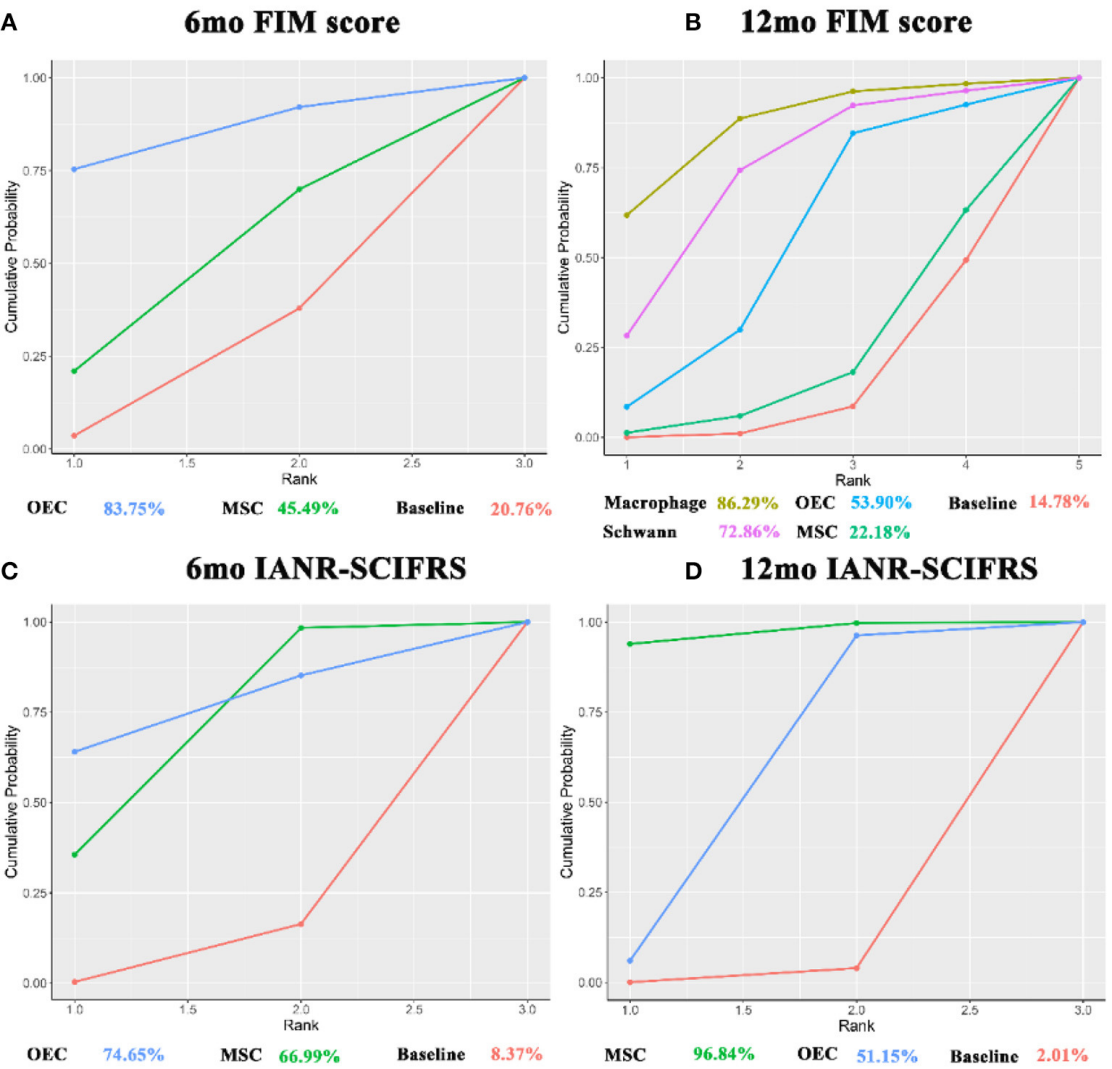
SUCRA for FIM score and IANR-SCIFRS. **(A)** SUCRA for 6mo FIM score; **(B)** SUCRA for 12mo FIM score; **(C)** SUCRA for 6mo IANR-SCIFRS; **(D)** SUCRA for 12mo IANR-SCIFRS. The x-axis represents the ranking and the y-axis represents the cumulative probability. The cumulative ranking and cumulative probability are constructed as a curve. The larger the area under the curve, the greater the benefit of the intervention. FIM, Functional independence measure; IANR-SCIFRS, International Association of Neurorestoratology Spinal Cord Injury Functional Rating Scale; mo, month; SUCRA, the surface under the cumulative ranking; MSC, mesenchymal stem cell; NSC, neural stem cell/neural progenitor cell; OEC, olfactory ensheathing cell.

By SUCRA (Figures 7A,B), in terms of the 6mo FIM score, OECs (83.75%) ranked first, followed by MSCs (45.49%); while for the 12mo FIM score, macrophages ranked first (86.29%), followed by Schwann cells (72.86%), and OECs (53.90%), while MSC (22.18%) ranked last.

### IANR-SCIFRS

For the IANR-SCIFRS at 6 months (6mo IANR-SCIFRS) (Rao et al., 2013b; Vaquero et al., 2016, 2017, 2018; Yang et al., 2021) and at 12 months (12mo IANR-SCIFRS) (Huang et al., 2012; Rao et al., 2013b; Chen et al., 2014; Vaquero et al., 2016, 2017, 2018; Wang et al., 2016; Yang et al., 2021), MSCs (6 months: 3.96, 0.62–6.97; 12 months: 5.54, 2.45–8.42) significantly increased the IANR-SCIFRS score after transplantation. There was no statistically significant difference between OECs and MSCs (Table 2F). SUCRA (Figures 7C,D) showed that OECs and MSCs ranked first at 6 months (74.65%) and at 12 months (96.84%), respectively.

The SUCRA value of each outcome and the ranking order is detailed in Table 3 and Supplementary Tables S6. GRADE analysis showed that the certainty of the evidence for all outcomes was relatively low for most outcomes (Supplementary Tables S7–S12).

**Table 3:** The ranking result of SUCRA.

**Rank**	**1**	**2**	**3**	**4**	**5**	**6**	**7**
6mo AIS	MSC	**Macrophage**	Schwann	NSC	OEC	Combined	Baseline
12mo AIS	**Macrophage**	OEC	MSC	Schwann	NSC	Combined	Baseline
6mo motor	NSC	MSC	Macrophage	Schwann	OEC	Combined	Baseline
12mo motor	NSC	**Macrophage**	MSC	Combined	OEC	Baseline	–
6mo light touch	**Macrophage**	MSC	NSC	OEC	Schwann	Baseline	–
12mo light touch	**Macrophage**	MSC	NSC	OEC	Baseline	–	–
6mo pinprick	MSC	**Macrophage**	NSC	OEC	Schwann	Baseline	–
12mo pinprick	**Macrophage**	MSC	NSC	OEC	Baseline	–	–
6mo FIM score	OEC	MSC	Baseline	–	–	–	–
12mo FIM score	**Macrophage**	Schwann	OEC	MSC	Baseline	–	–
6mo IANR-SCIFRS	OEC	MSC	Baseline	–	–	–	–
12mo IANR-SCIFRS	MSC	OEC	Baseline	–	–	–	–

*AIS, American Spinal Cord Injury Association (ASIA) Impairment Scale; FIM, functional independence measure; IANR-SCIFRS, International association of Neurorestoratology Spinal Cord Injury Functional Rating Scale; mo, month (s); MSC, mesenchymal stem cells; NSC, neural stem cell/neural progenitor cell; OEC, olfactory ensheathing cells; SUCRA, the surface under the cumulative ranking*.

### Adverse Events

In this study, 44 articles, involving 1,266 patients, described adverse events. Most were single-arm studies, lacking a control group. Therefore, adverse events in this study are presented as descriptive statistics only. Adverse events occurred 1,144 times in total. Table 4 shows that of the total adverse events, the number of AE per capita was <1 for both the OEC and MSC. Only two SAE occurred in patients with MSC and no deaths were reported. The most common SAE was cerebrospinal fluid leakage (44 cases), which was 46.81% of the SAE accumulated for all transplanted cells (Supplementary Table S13). SAE associated with cell transplantation may include pseudomeningocele (3 times), autonomic dysreflexia (5 times), and meningitis (3 times) in addition to cerebrospinal fluid leakage. Three deaths occurred in the perioperative period: 2 patients who received OECs (Huang et al., 2009) and 1 patient who received macrophages (Lammertse et al., 2012). Temporary anosmia commonly occurred within 7 days after autologous OEC transplantation, but gradually recovered at 7–14 days without special treatment. No article reported permanent anosmia.

**Table 4:** Adverse events of different transplanted cells.

**Cell**	**Sample size**	**No. of AE**	**AE_per capita**	**No. of mild-moderate AE**	**No. of SAE**	**SAE_per capita**	**No. of very SAE**
Combined	14	17	1.21	17	0	0	0
Macrophage	41	414	10.10	401	12	0.03	1
MSC	355	262	0.74	260	2	0.01	0
NSC	63	271	4.30	232	39	0.14	0
OEC	775	80	0.10	38	40	0.50	2
Schwann	18	100	5.56	99	1	0.01	0
Total	1,266	1,144	22.01	1,047	94	0.08	3

*AE, adverse event; MSC, mesenchymal stem cell; No, number; NSC, neural stem cell/neural progenitor cell; OEC, olfactory ensheathing cell; SAE: severe adverse event*.

### Pairwise Meta-Analysis

The results of the pairwise meta-analysis of various outcomes are shown in Supplementary Table S14; all comparisons were made relative to their baseline.

MSCs significantly improved the AIS grade at 6 and 12 months, and macrophages significantly improved the grade at 6 months. Only OECs and NSCs significantly improved the motor score at 12 months after transplantation. Macrophages significantly improved the light touch scores at 6 and 12 months, MSCs improved these scores at 6 months, and OECs improved these scores at 12 months. Macrophages significantly improved the pinprick scores at 6 and 12 months, MSCs improved the score at 6 months, and NSCs improved the score at 12 months. Macrophages, OECs, and Schwann cells significantly improved the FIM score at 12 months. For IANR-SCIFRS, only OEC significantly improved post-transplantation scores.

### Small-Sample Effect Test and Hypothesis Test of NMA

In the analyses of the 6mo AIS grade, 12mo AIS grade, motor score, and pinprick score, we found some evidence of small-sample effects (Supplementary Figures S1–S4) (Chhabra et al., 2009; Saito et al., 2012; Tabakow et al., 2013; Vaquero et al., 2016, 2018), and therefore performed a sensitivity analysis for these outcomes. There was no change in the ranking order of the different transplanted cells. Therefore, the study was therefore robust overall.

To demonstrate the hypothesis of homogeneity and transferability of NMA, in addition to confirming that there were no significant differences in interventions and statistical methods, we also compared the mean age of SCI patients in all articles (Supplementary Figure S5) and performed a meta-regression analysis (Supplementary Table S15) of baseline data, publication year, and cell types. All results showed that the hypothesis of homogeneity and transitivity of NMA was established. In the consistency test of NMA, the difference in DIC values calculated under the 2 different models was <1 (Supplementary Table S16), suggesting that the consistency hypothesis was established. The NMA fit was analyzed, and all outcome measures converged well and stabilized (Supplementary Figures S6–S17).

## Discussion

### Principal Findings

In this paper, the efficacy and safety of 6 different sources of transplanted cells, including MSCs, macrophages, NSC, OEC, Schwann cells, and combined (MSC combined with Schwann cell transplantation), for the treatment of SCI patients were comprehensively summarized by systematic review and NMA.

The combined quality of evidence evaluation and NMA showed that SCI patients who received macrophage, MSC, NSC, or OEC transplantation showed significant improvement in neurological function within 6–12 months. In the comparison of transplanted cells from different sources, SCI patients who received macrophages faired significantly better in FIM score improvement at 12 months than those who received MSCs or OECs. SCI patients who received OEC or Schwann cell transplantation faired significantly better in improving the FIM score at 12 months than SCI patients who received MSCs. However, MSCs were significantly superior to OECs in improving light touch and pinprick sensation at 12 months. SUCRA showed that MSCs and macrophages ranked in the top 2 for most outcome measures.

Macrophages are key effector cells of the inflammatory response after SCI. After SCI, due to ischemia and hypoxia of neural tissue, macrophages promote the formation of new blood vessels by up-regulating the release of vascular endothelial growth factor (VEGF-A) (Cattin et al., 2015), thereby providing oxygen to the damaged area. The newly formed blood vessels may provide migration channels for other cells to migrate to the SCI site (Huang et al., 2020b). In the trials included in this study, macrophages for transplantation were obtained by incubating autologous venous blood (200 ml) collected with a full thickness of skin harvested from the medial aspect of the patient's arm (Knoller et al., 2005; Lammertse et al., 2012). These macrophages co-incubated with skin can secrete brain-derived neurotrophic factor (BDNF), IL-1beta, and IL-6 (Knoller et al., 2005). BDNF is a polypeptide growth factor (Song et al., 2017) that has neuroprotective, cell survival, and synaptic plasticity effects in the injured spinal cord after binding to the tropomyosin-associated kinase (TrkB). FL receptor (Philippe et al., 1987; Alcántara et al., 1997; Mantilla et al., 2013, 2014). Macrophages can be polarized to M1 (classical activation) or M2 (alternative activation) (Mantovani et al., 2004; Beck et al., 2010; Gordon and Martinez, 2010) in different inflammatory environments. M1 macrophages promote the secondary inflammatory response after SCI, while M2 macrophages can promote axonal regeneration (David and Kroner, 2011). Macrophages co-incubated with skin tissue reduce the secretion of proinflammatory cytokines, such as TNF-α (Bomstein et al., 2003), and thus have the potential to differentiate into M2 macrophages. Li et al. (2019) proposed that BDNF can promote polarization of M1 macrophages into M2 macrophages, possibly *via* BDNF regulation of TrkB/PI3K/serine–threonine kinase 1 (AKT1). Additionally, disease duration was <14 days in patients who received macrophage transplantation as compared with patients who received other transplanted cells for SCI, suggesting that early macrophage transplantation in SCI patients may reduce the severity of the disease in the early stage post-injury, highlighting the effect of neurological recovery in the late stage of injury. This may therefore be a reason for the neurological recovery of patients after receiving macrophage transplantation. The possible mechanism is that, in the acute or subacute phase of SCI, cell death and inflammatory response are the heaviest, and after early transplantation, macrophages can play a role as early as possible and protect neural tissue (Shinozaki et al., 2021). However, there have been no relevant studies to support this idea. In summary, early transplantation of macrophages after SCI can have a positive impact on the recovery of neurological function in patients. Notably, however, TGF-beta secreted by M2 macrophages can activate astrocytes to promote glial scar formation (Song et al., 2019). Thus, modulating the balance of M1: M2 may be critical for the efficacy of macrophage transplantation in SCI patients (Hu et al., 2015).

Since Caplan (1991) first reported the application of MSCs in 1991, MSCs have been studied intensively (Bartlett et al., 2020). In response to VEGF and hepatocyte growth factor released at the site of injury (Zachar et al., 2016; Qu and Zhang, 2017) and/or regulated by the SDF-1α/CXCR4 (stromal cell-derived factor-1α/ C–X–C chemokine receptor 4) axis (Pelagalli et al., 2018), MSCs show “homing” properties and could migrate to the injury site of SCI. Existing studies have shown that (An et al., 2021; Suzuki and Sakai, 2021) the therapeutic effect of MSC is mainly exerted through paracrine activity, thereby protecting surviving neurons and oligodendrocytes (Rehman et al., 2004; Uccelli et al., 2008; Sorrell et al., 2009; Lim et al., 2019), promoting neovascularization (Hofer and Tuan, 2016), and inhibiting the inflammatory response at the injury site (Boido et al., 2014). In addition, MSCs can also assist in functional recovery by affecting macrophage polarization and controlling the effect of secondary injury after SCI (An et al., 2021). MSCs can promote the conversion of polarized macrophages/microglia from M1 to M2, improve the inflammatory environment, and promote recovery after SCI (Nakajima et al., 2012; Dooley et al., 2016; Zhou et al., 2016). Meta-analyses on animal models (Oliveri et al., 2014) or on clinical trials (Xu and Yang, 2019; Chen et al., 2021; Muthu et al., 2021) revealed the potential of MSCs to improve neurological function, which was generally consistent with this study. MSC can be extracted from autologous bone marrow, umbilical cord, or adipose tissue (Mahmoudian-Sani et al., 2017; Jayaram et al., 2019), which is a convenient source and may reduce potential ethical concerns. Our analysis showed that MSCs were significant in increasing AIS grade and improving both ASIA motor and sensory scores. Also, MSCs ranked in the top 2 for most outcome measures. Hence, MSCs may be one of the primary recommended transplanted cell types currently used to improve neurological function in patients with SCI. However, due to the lack of strong evidence, there is an urgent need for high-quality research evidence, particularly randomized controlled studies. Notably, there are still some controversies about the nomenclature of MSCs (Sipp et al., 2018) and cell identification (Viswanathan et al., 2019). As such, to avoid interference from confounding factors, we included articles that met the identification criteria according to the minimum identification criteria for MSC (Dominici et al., 2006). Compared with the results of a previously published meta-analysis of MSCs (Xu and Yang, 2019; Chen et al., 2021; Muthu et al., 2021), MSCs were shown to be significant in improving light touch and pinprick sensation at 6 and 12 months, AIS, and motor scores at 6 months.

Schwann cells are glial cells present in peripheral nerves (Anderson et al., 2017). OECs are sheath cells with both astrocyte and Schwann cell characteristics (Fairless and Barnett, 2005; Rao et al., 2013b). Although Schwann cells and OECs have different sources of acquisition, they play similar roles in SCI, such as the secretion of extracellular matrix molecules and neurotrophic factors (GDNF) and nerve growth factor (NGF) (Woodhall et al., 2001; Ekberg et al., 2012; Jessen and Arthur-Farraj, 2019), thereby exerting neuroprotective effects, and promoting myelination (Wu et al., 2003; Deng et al., 2015) and axonal regeneration (Graziadei and Graziadei, 1979; Takami et al., 2002; Su and He, 2010). Additionally, these cell types also have unique functions in SCI (Bartlett et al., 2020). Therefore, there is good scientific evidence for OECs or Schwann cells in the neurological recovery of SCI. Their ability to promote myelination and axonal regeneration may be why OECs or Schwann cells are superior to MSCs in improving the FIM score; however, in this index, due to the paucity of articles using this outcome index, more sufficient clinical evidence is needed to prove the difference in efficacy among the 3 cell types.

Notably, in cell culture, Schwann cell and OEC expression share similar phenotypic features, such as p75 (Barnett et al., 1993; Kocsis et al., 2009). The olfactory mucosa receives trigeminal innervation in addition to olfactory fibers, so that the influence of Schwann cells from trigeminal nerve fibers may not be completely excluded when preparing OEC (Wang et al., 2016). The potential interference of Schwann cells and the similarity of OEC and Schwann cells themselves may exist when OEC transplantation is performed, coupled with the difficulty in harvesting sufficient olfactory cells (Wang et al., 2016), which further increases the uncertainty of efficacy. Because OECs and Schwann cells play an important role in their corresponding position, acquisition of the two cells can affect the function of this site, which may influence olfactory abnormalities or lower limb paresthesia. In addition, compared with other transplanted cells, obtaining sufficient Schwann cells that meet clinical requirements requires not only complex infrastructure but also much manpower and time, making it difficult to popularize. In addition to this, the isolation of primary cells may be compromised by low yield or poor survival and may be contaminated with fibroblasts (Monje et al., 2021).

For the description of adverse events, due to the different definitions of SAE in different articles, we performed statistical description of the 44 included articles according to the classification of adverse reactions provided by NCICTC (2017) and the description of SAE in included studies (Bhanot et al., 2011; Lammertse et al., 2012; Satti et al., 2016; Curt et al., 2020). In terms of SAE, common complications related to cell transplantation are pseudomeningocele, autonomic dysreflexia, meningitis, and cerebrospinal fluid leakage. Among them, cerebrospinal fluid leakage occurred in patients who underwent resection of the vertebral plate, and opening of the dura mater followed by intramedullary injection as the route of transplantation. Cerebrospinal fluid leaks, pseudomeningocele, and meningitis tend to appear 1–2 weeks after receiving the transplant (Lima et al., 2010; Lammertse et al., 2012; Levi et al., 2018; Curt et al., 2020). In addition, Kishk et al. (2010) reported a case of a 27-year-old female patient with previous post-infection myelitis who developed acute disseminated encephalomyelitis 6 h after receiving her third MSC transplant and improved with treatment. This suggests that MSC may be contraindicated in patients with a history of myelitis. Two patients who underwent OEC transplantation in Huang et al. (2009) had fatal events due to hypertensive intracerebral hemorrhage and pulmonary infection. Additionally, the death in 1 patient who died after receiving macrophages (Lammertse et al., 2012) was due to quadriplegia caused by cervical SCI. The authors explained that the cause of death may have been related to obesity, and suggested that further studies should be conducted to explore the effect of obesity on macrophage transplantation.

The route of transplantation may be an important factor affecting prognosis. The intraspinal injection was the most commonly used transplantation route in the included studies (Supplementary Table S4). In terms of MSC, Mendonça et al. (2014), Park et al. (2012) used intraspinal injection and all reported well effects on the recovery of neurological function. Park et al. (2012) suggested that the “homing” properties of MSC may have disappeared in patients with chronic SCI, which is different from the acute phase. In addition, an animal model study comparing different transplantation routes concluded that intraspinal injection may be a safe and reliable method, and the number of transplanted cells is the largest (Shin et al., 2013). Chen et al. (2021), however, synthesized articles on MSC transplantation by different transplantation routes for the treatment of SCI for a meta-analysis and concluded that intrathecal injection may be the best transplantation route. However, due to the limitation of the included articles, we failed to analyze the differences in efficacy among different transplantation routes. In the future, a comparative study comparing the effects of different transplantation routes on efficacy is highly desirable.

### Future Research

Although our comprehensive analysis identified differences in the efficacy and safety of different transplanted cells, we concluded that cell transplantation has some efficacy for SCI treatment. However, due to the limitations of the original studies, the majority of the evidence in this paper is of low certainty. High quality randomized controlled studies are lacking and no definitive conclusions have been reached regarding the route of transplantation, the number of cells transplanted, time to transplantation after injury, preparation and preservation of transplanted cells, monitoring of cell survival after transplantation, and complications associated with transplantation. There is still a gap in standardized protocols for cell transplantation for SCI. Although not reported in the studies included here, according to Jeong et al. (2011), chromosomal abnormalities appear at the early passage after MSC transplantation and lead to the occurrence of tumors, which requires further in-depth analysis. Therefore, for a clearer comparison of the efficacy and safety of transplanted cells from different sources, it remains necessary to explore the above-mentioned still unclear conclusions. Recently, the study of cell transplantation combined with scaffold in the treatment of SCI is gradually carried out (Xiao et al., 2018; Chen et al., 2020; Deng et al., 2020; Kim et al., 2021; Tang et al., 2021). An observational study (Tang et al., 2021) with a follow-up of 2–5 years found varying degrees of recovery of sensory levels and increased defecation reflexes after cell transplantation combined with scaffold therapy in patients with acute or chronic SCI. This allows patients and physicians afflicted with spinal cord injury to have more expectations.

### Limitations

This study had the following limitations. Most included studies were phase I or II clinical studies, and some studies had small sample sizes. We included case–control studies and case series studies, in addition to randomized controlled trials. Due to the limitation of inclusion in the study, it is difficult for us to perform analysis, including disease duration, transplantation methods, and the total number of transplanted cells. In the safety analysis, due to the inability to construct a network plot, this study only performed descriptive statistics on adverse events and lacked comparative evidence.

## Conclusion

SCI patients who receive macrophages, MSCs, NSCs, or OECs showed benefits in terms of recovery of motor function, sensory function, and life independence. Among all cell types transplanted for SCI, MSCs are recommended. However, it remains necessary to explore the exact mechanism by which cell transplantation improves SCI, the effect of the choice of transplantation time window on the efficacy, the effect of the transplantation methods and the number of transplanted cells on the efficacy, and the monitoring of transplanted cell survival.

## Data Availability Statement

The original contributions presented in the study are included in the article/Supplementary Materials, further inquiries can be directed to the corresponding author.

## Author Contributions

XX, ZY, RW, and CM designed and wrote the study protocol. XX and ZY searched and selected the articles. XX and JR extracted the data. XX, YL, and FL analyzed the data. XX and ZY wrote the manuscript. All authors read and agree with the results and conclusions of this article. All authors contributed to the article and approved the submitted version.

## Conflict of Interest

The authors declare that the research was conducted in the absence of any commercial or financial relationships that could be construed as a potential conflict of interest.

## Publisher's Note

All claims expressed in this article are solely those of the authors and do not necessarily represent those of their affiliated organizations, or those of the publisher, the editors and the reviewers. Any product that may be evaluated in this article, or claim that may be made by its manufacturer, is not guaranteed or endorsed by the publisher.

## Abbreviations

AIS grade: American Spinal Cord Injury Association (ASIA) Impairment Scale
CI: credible interval
FIM: Functional independence measure
IANR-SCIFRS: International Association of Neurorestoratology Spinal Cord Injury Functional Rating Scale
MSC: mesenchymal stem cell
NMA: network meta-analysis
NSC: neural stem cell/neural progenitor cell
OEC: olfactory ensheathing cell
RCT: randomized controlled trial
SAE: severe adverse events
SCI: spinal cord injury.

## References

[B1] Ades A. E. Sculpher M. Sutton A. Abrams K. Cooper N. Welton N. . (2006). Bayesian methods for evidence synthesis in cost-effectiveness analysis. Pharmacoeconomics. 24, 1–19. 10.2165/00019053-200624010-00001 16445299

[B2] Ahuja C. S. Wilson J. R. Nori S. Kotter M. R. N. Druschel C. Curt A. . (2017). Traumatic spinal cord injury. Nat. Rev. Dis. Primers 3, 17018. 10.1038/nrdp.2017.18 28447605

[B3] Alcántara S. Frisén J. del Río J. A. Soriano E. Barbacid M. Silos-Santiago I. (1997). TrkB signaling is required for postnatal survival of CNS neurons and protects hippocampal and motor neurons from axotomy-induced cell death. J. Neurosci. 17, 3623–3633. 10.1523/JNEUROSCI.17-10-03623.1997 9133385 PMC6573670

[B4] An N. Yang J. Wang H. Sun S. Wu H. Li L. . (2021). Mechanism of mesenchymal stem cells in spinal cord injury repair through macrophage polarization. Cell Biosci. 11, 41. 10.1186/s13578-021-00554-z PMC7903655 33622388

[B5] Anderson K. D. Guest J. D. Dietrich W. D. Bartlett Bunge M. Curiel R. Dididze M. . (2017). Safety of autologous human schwann cell transplantation in subacute thoracic spinal cord injury. J. Neurotrauma 34, 2950–2963. 10.1089/neu.2016.4895 28225648

[B6] Assinck P. Duncan G. J. Hilton B. J. Plemel J. R. Tetzlaff W. (2017). Cell transplantation therapy for spinal cord injury. Nat. Neurosci. 20, 637–647. 10.1038/nn.4541 28440805

[B7] Badhiwala J. H. Wilson J. R. Fehlings M. G. (2019). Global burden of traumatic brain and spinal cord injury. Lancet Neurol. 18, 24–25. 10.1016/S1474-4422(18)30444-7 30497967

[B8] Badhiwala J. H. Wilson J. R. Witiw C. D. Harrop J. S. Vaccaro A. R. Aarabi B. . (2021). The influence of timing of surgical decompression for acute spinal cord injury: a pooled analysis of individual patient data. Lancet Neurol. 20, 117–126. 10.1016/S1474-4422(20)30406-3 33357514

[B9] Barnett S. C. Hutchins A. M. Noble M. (1993). Purification of olfactory nerve ensheathing cells from the olfactory bulb. Dev. Biol. 155, 337–350. 10.1006/dbio.1993.1033 7679359

[B10] Bartlett R. D. Burley S. Ip M. Phillips J. B. Choi D. (2020). Cell therapies for spinal cord injury: trends and challenges of current clinical trials. Neurosurgery 87, E456–E472. 10.1093/neuros/nyaa149 32497197

[B11] Beck K. D. Nguyen H. X. Galvan M. D. Salazar D. L. Woodruff T. M. Anderson A. J. (2010). Quantitative analysis of cellular inflammation after traumatic spinal cord injury: evidence for a multiphasic inflammatory response in the acute to chronic environment. Brain 133, 433–447. 10.1093/brain/awp322 20085927 PMC2858013

[B12] Bhanot Y. Rao S. Ghosh D. Balaraju S. Radhika C. R. Satish Kumar K. V. (2011). Autologous mesenchymal stem cells in chronic spinal cord injury. Br. J. Neurosurg. 25, 516–522. 10.3109/02688697.2010.550658 21749185

[B13] Boido M. Piras A. Valsecchi V. Spigolon G. Mareschi K. Ferrero I. . (2014). Human mesenchymal stromal cell transplantation modulates neuroinflammatory milieu in a mouse model of amyotrophic lateral sclerosis. Cytotherapy 16, 1059–1072. 10.1016/j.jcyt.2014.02.003 24794182

[B14] Bomstein Y. Marder J. B. Vitner K. Smirnov I. Lisaey G. Butovsky O. . (2003). Features of skin-coincubated macrophages that promote recovery from spinal cord injury. J. Neuroimmunol. 142, 10–16. 10.1016/S0165-5728(03)00260-1 14512160

[B15] Brignardello-Petersen R. Bonner A. Alexander P. E. Siemieniuk R. A. Furukawa T. A. Rochwerg B. . (2018). Advances in the GRADE approach to rate the certainty in estimates from a network meta-analysis. J. Clin. Epidemiol. 93, 36–44. 10.1016/j.jclinepi.2017.10.005 29051107

[B16] Brignardello-Petersen R. Florez I. D. Izcovich A. Santesso N. Hazlewood G. Alhazanni W. . (2020a). GRADE approach to drawing conclusions from a network meta-analysis using a minimally contextualised framework. BMJ 371, m3900. 10.1136/bmj.m3900 33177059

[B17] Brignardello-Petersen R. Izcovich A. Rochwerg B. Florez I. D. Hazlewood G. Alhazanni W. . (2020b). GRADE approach to drawing conclusions from a network meta-analysis using a partially contextualised framework. BMJ 371, m3907. 10.1136/bmj.m3907 33172877

[B18] Caplan A. I. (1991). Mesenchymal stem cells. J. Orthop. Res. 9, 641–650. 10.1002/jor.1100090504 1870029

[B19] Cattin A. L. Burden J. J. Van Emmenis L. Mackenzie F. E. Hoving J. J. Garcia Calavia N. . (2015). Macrophage-induced blood vessels guide Schwann cell-mediated regeneration of peripheral nerves. Cell 162, 1127–1139. 10.1016/j.cell.2015.07.021 26279190 PMC4553238

[B20] Chen L. Huang H. Xi H. Zhang F. Liu Y. Chen D. . (2014). A prospective randomized double-blind clinical trial using a combination of olfactory ensheathing cells and Schwann cells for the treatment of chronic complete spinal cord injuries. Cell Transplant. 23, (Suppl. 1), S35–S44. 10.3727/096368914X685014 25333925

[B21] Chen W. Zhang Y. Yang S. Sun J. Qiu H. Hu X. . (2020). NeuroRegen scaffolds combined with autologous bone marrow mononuclear cells for the repair of acute complete spinal cord injury: a 3-year clinical study. Cell Transplant. 29, 963689720950637. 10.1177/0963689720950637 32862715 PMC7784506

[B22] Chen W. C. Liu W. F. Bai Y. Y. Zhou Y. Y. Zhang Y. Wang C. M. . (2021). Transplantation of mesenchymal stem cells for spinal cord injury: a systematic review and network meta-analysis. J. Transl. Med. 19, 178. 10.1186/s12967-021-02843-0 33910588 PMC8082850

[B23] Cheng H. Liu X. Hua R. Dai G. Wang X. Gao J. . (2014). Clinical observation of umbilical cord mesenchymal stem cell transplantation in treatment for sequelae of thoracolumbar spinal cord injury. J. Transl. Med. 12, 253. 10.1186/s12967-014-0253-7 25209445 PMC4172930

[B24] Chhabra H. S. Nigam V. Khan T. A. H. Lima C. Sachdeva S. Mittal A. . (2009). Autologous mucosal transplant in chronic spinal cord injury: an Indian Pilot Study. Spinal Cord. 47, 887–895. 10.1038/sc.2009.54 19488051

[B25] Collaborators GTBIaSCI (2019). Global, regional, and national burden of traumatic brain injury and spinal cord injury, 1990-2016: a systematic analysis for the Global Burden of Disease Study 2016. Lancet Neurol. 18, 56–87. 10.1016/S1474-4422(18)30415-0 30497965 PMC6291456

[B26] Committee AaIIS (2019). The 2019 revision of the International Standards for neurological classification of spinal cord injury (ISNCSCI)-what's new? Spinal Cord 57, 815–817. 10.1038/s41393-019-0350-9 31530900

[B27] Curt A. Hsieh J. Schubert M. Hupp M. Friedl S. Freund P. . (2020). The damaged spinal cord is a suitable target for stem cell transplantation. Neurorehabil. Neural Repair. 34, 758–768. 10.1177/1545968320935815 32698674

[B28] Curtis E. Martin J. R. Gabel B. Sidhu N. Rzesiewicz T. K. Mandeville R. . (2018). A first-in-human, phase I study of neural stem cell transplantation for chronic spinal cord injury. Cell Stem Cell 22, 941–950.e6. 10.1016/j.stem.2018.05.014 29859175

[B29] David S. Kroner A. (2011). Repertoire of microglial and macrophage responses after spinal cord injury. Nat. Rev. Neurosci. 12, 388–399. 10.1038/nrn3053 21673720

[B30] Deng L. X. Walker C. Xu X. M. (2015). Schwann cell transplantation and descending propriospinal regeneration after spinal cord injury. Brain Res. 1619, 104–114. 10.1016/j.brainres.2014.09.038 25257034 PMC4375094

[B31] Deng W. S. Ma K. Liang B. Liu X. Y. Xu H. Y. Zhang J. . (2020). Collagen scaffold combined with human umbilical cord-mesenchymal stem cells transplantation for acute complete spinal cord injury. Neural Regen Res. 15, 1686–1700. 10.4103/1673-5374.276340 32209773 PMC7437585

[B32] Dickson H. G. Köhler F. (1995). Interrater reliability of the 7-level functional independence measure (FIM). Scand. J. Rehabil. Med. 27, 253–256. 8650510

[B33] Dominici M. Le Blanc K. Mueller I. Slaper-Cortenbach I. Marini F. Krause D. . (2006). Minimal criteria for defining multipotent mesenchymal stromal cells. The International Society for Cellular Therapy position statement. Cytotherapy 8, 315–317. 10.1080/14653240600855905 16923606

[B34] Dooley D. Lemmens E. Vangansewinkel T. Le Blon D. Hoornaert C. Ponsaerts P. . (2016). Cell-based delivery of interleukin-13 directs alternative activation of macrophages resulting in improved functional outcome after spinal cord injury. Stem Cell Rep. 7, 1099–1115. 10.1016/j.stemcr.2016.11.005 27974221 PMC5161742

[B35] Ekberg J. A. Amaya D. Mackay-Sim A. St John J. A. (2012). The migration of olfactory ensheathing cells during development and regeneration. Neurosignals 20, 147–158. 10.1159/000330895 22456085

[B36] Fairless R. Barnett S. C. (2005). Olfactory ensheathing cells: their role in central nervous system repair. Int. J. Biochem. Cell Biol. 37, 693–699. 10.1016/j.biocel.2004.10.010 15694828

[B37] Gant K. L. Guest J. D. Palermo A. E. Vedantam A. Jimsheleishvili G. Bunge M. B. . (2021). Phase 1 safety trial of autologous human schwann cell transplantation in chronic spinal cord injury. J. Neurotrauma. 39, 285–299. 10.1089/neu.2020.7590 33757304 PMC9360180

[B38] Ghobrial G. M. Anderson K. D. Dididze M. Martinez-Barrizonte J. Sunn G. H. Gant K. L. . (2017). Human neural stem cell transplantation in chronic cervical spinal cord injury: functional outcomes at 12 months in a phase II clinical trial. Neurosurgery 64, 87–91. 10.1093/neuros/nyx242 28899046

[B39] Gordon S. Martinez F. O. (2010). Alternative activation of macrophages: mechanism and functions. Immunity 32, 593–604. 10.1016/j.immuni.2010.05.007 20510870

[B40] Graziadei G. A. Graziadei P. P. (1979). Neurogenesis and neuron regeneration in the olfactory system of mammals. II Degeneration and reconstitution of the olfactory sensory neurons after axotomy. J. Neurocytol. 8, 197–213. 10.1007/BF01175561 469573

[B41] Hao D. Du J. Yan L. He B. Qi X. Yu S. . (2021). Trends of epidemiological characteristics of traumatic spinal cord injury in China, 2009-2018. Euro. Spine J. 30, 3115–3127. 10.1007/s00586-021-06957-3 34392419

[B42] Hejrati N. Fehlings M. G. (2021). A review of emerging neuroprotective and neuroregenerative therapies in traumatic spinal cord injury. Curr. Opin. Pharmacol. 60, 331–340. 10.1016/j.coph.2021.08.009 34520943

[B43] Hofer H. R. Tuan R. S. (2016). Secreted trophic factors of mesenchymal stem cells support neurovascular and musculoskeletal therapies. Stem Cell Res. Ther. 7, 131. 10.1186/s13287-016-0394-0 27612948 PMC5016979

[B44] Hu X. Leak R. K. Shi Y. Suenaga J. Gao Y. Zheng P. . (2015). Microglial and macrophage polarization-new prospects for brain repair. Nat. Rev. Neurol. 11, 56–64. 10.1038/nrneurol.2014.207 25385337 PMC4395497

[B45] Huang H. Chen L. Xi H. Wang Q. Zhang J. Liu Y. . (2009). Olfactory ensheathing cells transplantation for central nervous system diseases in 1,255 patients. Zhongguo Xiu Fu Chong Jian Wai Ke Za Zhi 23, 14–20. 19192871

[B46] Huang H. Xi H. Chen L. Zhang F. Liu Y. (2012). Long-term outcome of olfactory ensheathing cell therapy for patients with complete chronic spinal cord injury. Cell Transplant. 21(Suppl. 1), S23–S31. 10.3727/096368912X633734 22507677

[B47] Huang H. Young W. Skaper S. Chen L. Moviglia G. Saberi H. . (2020a). Clinical neurorestorative therapeutic guidelines for spinal cord injury (IANR/CANR version 2019). J. Orthopaedic Transl. 20, 14–24. 10.1016/j.jot.2019.10.006 31908929 PMC6939117

[B48] Huang Z. Powell R. Phillips J. B. Haastert-Talini K. (2020b). Perspective on schwann cells derived from induced pluripotent stem cells in peripheral nerve tissue engineering. Cells 9, 2497. 10.3390/cells9112497 33213068 PMC7698557

[B49] Hur J. W. Cho T. H. Park D. H. Lee J. B. Park J. Y. Chung Y. G. (2016). Intrathecal transplantation of autologous adipose-derived mesenchymal stem cells for treating spinal cord injury: a human trial. J. Spinal Cord Med. 39, 655–664. 10.1179/2045772315Y.0000000048 26208177 PMC5137573

[B50] Hutton B. Salanti G. Caldwell D. M. Chaimani A. Schmid C. H. Cameron C. . (2015). The PRISMA extension statement for reporting of systematic reviews incorporating network meta-analyses of health care interventions: checklist and explanations. Ann. Intern. Med. 162, 777–784. 10.7326/M14-2385 26030634

[B51] Iwatsuki K. Tajima F. Ohnishi Y. Nakamura T. Ishihara M. Hosomi K. . (2016). A pilot clinical study of olfactory mucosa autograft for chronic complete spinal cord injury. Neurol. Med. Chir. 56, 285–292. 10.2176/nmc.oa.2015-0320 27053327 PMC4908071

[B52] Jayaram P. Ikpeama U. Rothenberg J. B. Malanga G. A. (2019). Bone marrow-derived and adipose-derived mesenchymal stem cell therapy in primary knee osteoarthritis: a narrative review. PM R 11, 177–191. 10.1016/j.pmrj.2018.06.019 30010050

[B53] Jeong J. O. Han J. W. Kim J. M. Cho H. J. Park C. Lee N. . (2011). Malignant tumor formation after transplantation of short-term cultured bone marrow mesenchymal stem cells in experimental myocardial infarction and diabetic neuropathy. Circ. Res. 108, 1340–1347. 10.1161/CIRCRESAHA.110.239848 21493893 PMC3109741

[B54] Jessen K. R. Arthur-Farraj P. (2019). Repair Schwann cell update: adaptive reprogramming, EMT, and stemness in regenerating nerves. Glia 67, 421–437. 10.1002/glia.23532 30632639

[B55] Kim K. D. Lee K. S. Coric D. Chang J. J. Harrop J. S. Theodore N. . (2021). A study of probable benefit of a bioresorbable polymer scaffold for safety and neurological recovery in patients with complete thoracic spinal cord injury: 6-month results from the INSPIRE study. J. Neurosurg. Spine 34, 808–817. 10.3171/2020.8.SPINE191507 33545674

[B56] Kirshblum S. C. Burns S. P. Biering-Sorensen F. Donovan W. Graves D. E. Jha A. . (2011). International standards for neurological classification of spinal cord injury (revised 2011). J. Spinal Cord Med. 34, 535–546. 10.1179/204577211X13207446293695 22330108 PMC3232636

[B57] Kishk N. A. Gabr H. Hamdy S. Afifi L. Abokresha N. Mahmoud H. . (2010). Case control series of intrathecal autologous bone marrow mesenchymal stem cell therapy for chronic spinal cord injury. Neurorehabil. Neural Repair. 24, 702–708. 10.1177/1545968310369801 20660620

[B58] Knoller N. Auerbach G. Fulga V. Zelig G. Attias J. Bakimer R. . (2005). Clinical experience using incubated autologous macrophages as a treatment for complete spinal cord injury: phase I study results. J. Neurosurg. Spine 3, 173–181. 10.3171/spi.2005.3.3.0173 16235699

[B59] Kocsis J. D. Lankford K. L. Sasaki M. Radtke C. (2009). Unique *in vivo* properties of olfactory ensheathing cells that may contribute to neural repair and protection following spinal cord injury. Neurosci. Lett. 456, 137–142. 10.1016/j.neulet.2008.08.093 19429149 PMC2713444

[B60] Lammertse D. P. Jones L. A. Charlifue S. B. Kirshblum S. C. Apple D. F. Ragnarsson K. T. . (2012). Autologous incubated macrophage therapy in acute, complete spinal cord injury: results of the phase 2 randomized controlled multicenter trial. Spinal Cord. 50, 661–671. 10.1038/sc.2012.39 22525310

[B61] Larocca T. F. Macêdo C. T. Souza B. S. F. Andrade-Souza Y. M. Villarreal C. F. Matos A. C. . (2017). Dos Santos, Image-guided percutaneous intralesional administration of mesenchymal stromal cells in subjects with chronic complete spinal cord injury: a pilot study. Cytotherapy 19, 1189–1196. 10.1016/j.jcyt.2017.06.006 28760352

[B62] Levi A. D. Anderson K. D. Okonkwo D. O. Park P. Bryce T. N. Kurpad S. N. . (2019). Clinical outcomes from a multi-center study of human neural stem cell transplantation in chronic cervical spinal cord injury. J. Neurotrauma 36, 891–902. 10.1089/neu.2018.5843 30180779

[B63] Levi A. D. Okonkwo D. O. Park P. Jenkins A. L. 3rd Kurpad S. N. Parr A. M. Ganju A. . (2018). Emerging safety of intramedullary transplantation of human neural stem cells in chronic cervical and thoracic spinal cord injury. Neurosurgery 82, 562–575. 10.1093/neuros/nyx250 28541431

[B64] Li L. Adnan H. Xu B. Wang J. Wang C. Li F. . (2015). Effects of transplantation of olfactory ensheathing cells in chronic spinal cord injury: a systematic review and meta-analysis. Euro. Spine J. 24, 919–930. 10.1007/s00586-014-3416-6 25001890

[B65] Li M. Xu J. Mei X. Chi G. Li L. Song Y. . (2019). Regulatory effects of dermal papillary pluripotent stem cells on polarization of macrophages from M1 to M2 phenotype in vitro. Transpl. Immunol. 52, 57–67. 10.1016/j.trim.2018.11.003 30458295

[B66] Lim W. L. Liau L. L. Ng M. H. Chowdhury S. R. Law J. X. (2019). Current progress in tendon and ligament tissue engineering. Tissue Eng. Regener. Med. 16, 549–571. 10.1007/s13770-019-00196-w 31824819 PMC6879704

[B67] Lima C. Escada P. Pratas-Vital J. Branco C. Arcangeli C. A. Lazzeri G. . (2010). Olfactory mucosal autografts and rehabilitation for chronic traumatic spinal cord injury. Neurorehabil. Neural Repair. 24, 10–22. 10.1177/1545968309347685 19794133

[B68] Lima C. Pratas-Vital J. Escada P. Hasse-Ferreira A. Capucho C. Peduzzi J. D. (2006). Olfactory mucosa autografts in human spinal cord injury: a pilot clinical study. J. Spinal Cord Med. 29, 191–203; discussion: 204–206. 10.1080/10790268.2006.11753874 16859223 PMC1864811

[B69] Lumley T. (2002). Network meta-analysis for indirect treatment comparisons. Stat. Med. 21, 2313–2324. 10.1002/sim.1201 12210616

[B70] Mackay-Sim A. Féron F. Cochrane J. Bassingthwaighte L. Bayliss C. Davies W. . (2008). Autologous olfactory ensheathing cell transplantation in human paraplegia: a 3-year clinical trial. Brain 131, 2376–2386. 10.1093/brain/awn173 18689435 PMC2525447

[B71] Mahmoudian-Sani M. R. Mehri-Ghahfarrokhi A. Hashemzadeh-Chaleshtori M. Saidijam M. Jami M. S. (2017). Comparison of three types of mesenchymal stem cells (bone marrow, adipose tissue, and umbilical cord-derived) as potential sources for inner ear regeneration. Int. Tinnitus J. 21, 122–127. 10.5935/0946-5448.20170023 29336130

[B72] Mantilla C. B. Gransee H. M. Zhan W. Z. Sieck G. C. (2013). Motoneuron BDNF/TrkB signaling enhances functional recovery after cervical spinal cord injury. Exp. Neurol. 247, 101–109. 10.1016/j.expneurol.2013.04.002 23583688 PMC3742616

[B73] Mantilla C. B. Greising S. M. Stowe J. M. Zhan W. Z. Sieck G. C. (2014). TrkB kinase activity is critical for recovery of respiratory function after cervical spinal cord hemisection. Exp. Neurol. 261, 190–195. 10.1016/j.expneurol.2014.05.027 24910201 PMC4194245

[B74] Mantovani A. Sica A. Sozzani S. Allavena P. Vecchi A. Locati M. (2004). The chemokine system in diverse forms of macrophage activation and polarization. Trends Immunol. 25, 677–686. 10.1016/j.it.2004.09.015 15530839

[B75] Mendonça M. V. Larocca T. F. de Freitas Souza B. S. Villarreal C. F. Silva L. F. Matos A. C. . (2014). Safety and neurological assessments after autologous transplantation of bone marrow mesenchymal stem cells in subjects with chronic spinal cord injury. Stem Cell Res. Ther. 5, 126. 10.1186/scrt516 25406723 PMC4445989

[B76] Mills E. J. Thorlund K. Ioannidis J. P. (2013). Demystifying trial networks and network meta-analysis. BMJ 346, f2914. 10.1136/bmj.f2914 23674332

[B77] Monje P. V. Deng L. Xu X.-M. (2021). Human Schwann cell transplantation for spinal cord injury: prospects and challenges in translational medicine. Front. Cell. Neurosci. 15, 690894. 10.3389/fncel.2021.690894 34220455 PMC8249939

[B78] Moola S. Munn Z. Tufanaru C. Aromataris E. Sears K. Sfetcu R. . (2020). Chapter 7: systematic reviews of etiology and risk, in JBI Manual for Evidence Synthesis, eds Aromataris E. Munn Z. (JBI) 258–264.

[B79] Muthu S. Jeyaraman M. Gulati A. Arora A. (2021). Current evidence on mesenchymal stem cell therapy for traumatic spinal cord injury: systematic review and meta-analysis. Cytotherapy 23, 186–197. 10.1016/j.jcyt.2020.09.007 33183980

[B80] Nakajima H. Uchida K. Guerrero A. R. Watanabe S. Sugita D. Takeura N. . (2012). Transplantation of mesenchymal stem cells promotes an alternative pathway of macrophage activation and functional recovery after spinal cord injury. J. Neurotrauma 29, 1614–1625. 10.1089/neu.2011.2109 22233298 PMC3353766

[B81] NCICTC (2017). Common Terminology Criteria for Adverse Events (CTCAE) v5.0. U.S. Department of Health and Human Services: National cancer institute Division of Cancer Treatment and Diagnosis. Avaialble online at: https://ctep.cancer.gov/protocolDevelopment/electronic_applications/ctc.htm#ctc_50 (accessed May 15, 2020).

[B82] Oliveri R. S. Bello S. Biering-Sørensen F. (2014). Mesenchymal stem cells improve locomotor recovery in traumatic spinal cord injury: systematic review with meta-analyses of rat models. Neurobiol. Dis. 62, 338–353. 10.1016/j.nbd.2013.10.014 24148857

[B83] Oraee-Yazdani S. Hafizi M. Atashi A. Ashrafi F. Seddighi A. S. Hashemi S. M. . (2016). Co-transplantation of autologous bone marrow mesenchymal stem cells and Schwann cells through cerebral spinal fluid for the treatment of patients with chronic spinal cord injury: safety and possible outcome. Spinal Cord. 54, 102–109. 10.1038/sc.2015.142 26526896

[B84] Park J. H. Kim D. Y. Sung I. Y. Choi G. H. Jeon M. H. Kim K. K. . (2012). Long-term results of spinal cord injury therapy using mesenchymal stem cells derived from bone marrow in humans. Neurosurgery 70, 1238–1247; discussion: 1247. 10.1227/NEU.0b013e31824387f9 22127044

[B85] Pelagalli A. Nardelli A. Lucarelli E. Zannetti A. Brunetti A. (2018). Autocrine signals increase ovine mesenchymal stem cells migration through Aquaporin-1 and CXCR4 overexpression. J. Cell. Physiol. 233, 6241–6249. 10.1002/jcp.26493 29345324

[B86] Philippe J. Chick W. L. Habener J. F. (1987). Multipotential phenotypic expression of genes encoding peptide hormones in rat insulinoma cell lines. J. Clin. Invest. 79, 351–358. 10.1172/JCI112819 2879852 PMC424070

[B87] Pukos N. Goodus M. T. Sahinkaya F. R. McTigue D. M. (2019). Myelin status and oligodendrocyte lineage cells over time after spinal cord injury: what do we know and what still needs to be unwrapped? Glia 67, 2178–2202. 10.1002/glia.23702 31444938 PMC7217327

[B88] Qu J. Zhang H. (2017). Roles of mesenchymal stem cells in spinal cord injury. Stem Cells Int. 2017, 5251313. 10.1155/2017/5251313 28630630 PMC5467343

[B89] Rao Y. Zhu W. Guo Y. Jia C. Qi R. Qiao R. . (2013b). Long-term outcome of olfactory ensheathing cell transplantation in six patients with chronic complete spinal cord injury. Cell Transplant. 22(Suppl. 1), S21–S25. 10.3727/096368913X672127 23992752

[B90] Rao Y. Zhu W. Liu H. Jia C. Zhao Q. Wang Y. (2013a). Clinical application of olfactory ensheathing cells in the treatment of spinal cord injury. J. Int. Med. Res. 41, 473–481. 10.1177/0300060513476426 23569013

[B91] Rehman J. Traktuev D. Li J. Merfeld-Clauss S. Temm-Grove C. J. Bovenkerk J. E. . (2004). Secretion of angiogenic and antiapoptotic factors by human adipose stromal cells. Circulation 109, 1292–1298. 10.1161/01.CIR.0000121425.42966.F1 14993122

[B92] Saberi H. Moshayedi P. Aghayan H. R. Arjmand B. Hosseini S. K. Emami-Razavi S. H. . (2008). Treatment of chronic thoracic spinal cord injury patients with autologous Schwann cell transplantation: an interim report on safety considerations and possible outcomes. Neurosci. Lett. 443, 46–50. 10.1016/j.neulet.2008.07.041 18662744

[B93] Saito F. Nakatani T. Iwase M. Maeda Y. Murao Y. Suzuki Y. . (2012). Administration of cultured autologous bone marrow stromal cells into cerebrospinal fluid in spinal injury patients: a pilot study. Restor. Neurol. Neurosci. 30, 127–136. 10.3233/RNN-2011-0629 22232031

[B94] Salanti G. Ades A. E. Ioannidis J. P. (2011). Graphical methods and numerical summaries for presenting results from multiple-treatment meta-analysis: an overview and tutorial. J. Clin. Epidemiol. 64, 163–171. 10.1016/j.jclinepi.2010.03.016 20688472

[B95] Satti H. S. Waheed A. Ahmed P. Ahmed K. Akram Z. Aziz T. . (2016). Autologous mesenchymal stromal cell transplantation for spinal cord injury: a phase I pilot study. Cytotherapy 18, 518–522. 10.1016/j.jcyt.2016.01.004 26971680

[B96] Shin D. A. Kim J. M. Kim H. I. Yi S. Ha Y. Yoon D. H. . (2013). Comparison of functional and histological outcomes after intralesional, intracisternal, and intravenous transplantation of human bone marrow-derived mesenchymal stromal cells in a rat model of spinal cord injury. Acta Neurochir. 155, 1943–1950. 10.1007/s00701-013-1799-5 23821338

[B97] Shin J. C. Kim K. N. Yoo J. Kim I. S. Yun S. Lee H. . (2015). Clinical trial of human fetal brain-derived neural stem/progenitor cell transplantation in patients with traumatic cervical spinal cord injury. Neural Plast. 2015, 630932. 10.1155/2015/630932 26568892 PMC4619963

[B98] Shinozaki M. Nagoshi N. Nakamura M. Okano H. (2021). Mechanisms of stem cell therapy in spinal cord injuries. Cells 10, 2676. 10.3390/cells10102676 34685655 PMC8534136

[B99] Sipp D. Robey P. G. Turner L. (2018). Clear up this stem-cell mess. Nature 561, 455–457. 10.1038/d41586-018-06756-9 30258150

[B100] Song G. Yang R. Zhang Q. Chen L. Huang D. Zeng J. . (2019). TGF-β secretion by M2 macrophages induces glial scar formation by activating astrocytes *in vitro*. J. Mol. Neurosci. 69, 324–332. 10.1007/s12031-019-01361-5 31327154

[B101] Song M. Martinowich K. Lee F. S. (2017). BDNF at the synapse: why location matters. Mol. Psychiatry 22, 1370–1375. 10.1038/mp.2017.144 28937692 PMC5646361

[B102] Sorrell J. M. Baber M. A. Caplan A. I. (2009). Influence of adult mesenchymal stem cells on in vitro vascular formation. Tissue Eng. Part A 15, 1751–1761. 10.1089/ten.tea.2008.0254 19196139 PMC2792097

[B103] Spiegelhalter D. J. Best N. G. Carlin B. P. Van Der Linde A. (2002). Bayesian measures of model complexity and fit. J. R. Stat. Soc. Ser. B 64, 583–639. 10.1111/1467-9868.00353

[B104] Su Z. He C. (2010). Olfactory ensheathing cells: biology in neural development and regeneration. Prog. Neurobiol. 92, 517–532. 10.1016/j.pneurobio.2010.08.008 20837090

[B105] Suzuki H. Sakai T. (2021). Current concepts of stem cell therapy for chronic spinal cord injury. Int. J. Mol. Sci. 22, 7435. 10.3390/ijms22147435 34299053 PMC8308009

[B106] Tabakow P. Jarmundowicz W. Czapiga B. Fortuna W. Miedzybrodzki R. Czyz M. . (2013). Transplantation of autologous olfactory ensheathing cells in complete human spinal cord injury. Cell Transplant. 22, 1591–1612. 10.3727/096368912X663532 24007776

[B107] Takami T. Oudega M. Bates M. L. Wood P. M. Kleitman N. Bunge M. B. (2002). Schwann cell but not olfactory ensheathing glia transplants improve hindlimb locomotor performance in the moderately contused adult rat thoracic spinal cord. J. Neurosci. 22, 6670–6681. 10.1523/JNEUROSCI.22-15-06670.2002 12151546 PMC6758124

[B108] Tang F. Tang J. Zhao Y. Zhang J. Xiao Z. Chen B. . (2021). Long-term clinical observation of patients with acute and chronic complete spinal cord injury after transplantation of NeuroRegen scaffold. Sci. China Life Sci. 10.1007/s11427-021-1985-5 34406569

[B109] Tufanaru C. Munn Z. Aromataris E. Campbell J. Hopp L. (2020). Chapter 3: systematic reviews of effectiveness, in JBI Manual for Evidence Synthesis, eds Aromataris E. Munn Z. (JBI) 127–130.

[B110] Uccelli A. Moretta L. Pistoia V. (2008). Mesenchymal stem cells in health and disease. Nat. Rev. Immunol. 8, 726–736. 10.1038/nri2395 19172693

[B111] van Valkenhoef G. Dias S. Ades A. E. Welton N. J. (2016). Automated generation of node-splitting models for assessment of inconsistency in network meta-analysis. Res. Synth. Methods 7, 80–93. 10.1002/jrsm.1167 26461181 PMC5057346

[B112] Vaquero J. Zurita M. Rico M. A. Aguayo C. Fernandez C. Rodriguez-Boto G. . (2018). Cell therapy with autologous mesenchymal stromal cells in post-traumatic syringomyelia. Cytotherapy 20, 796–805. 10.1016/j.jcyt.2018.04.006 29784434

[B113] Vaquero J. Zurita M. Rico M. A. Bonilla C. Aguayo C. Fernández C. . (2017). Repeated subarachnoid administrations of autologous mesenchymal stromal cells supported in autologous plasma improve quality of life in patients suffering incomplete spinal cord injury. Cytotherapy 19, 349–359. 10.1016/j.jcyt.2016.12.002 28089079

[B114] Vaquero J. Zurita M. Rico M. A. Bonilla C. Aguayo C. Montilla J. . (2016). An approach to personalized cell therapy in chronic complete paraplegia: the Puerta de Hierro phase I/II clinical trial. Cytotherapy 18, 1025–1036. 10.1016/j.jcyt.2016.05.003 27311799

[B115] Viswanathan S. Shi Y. Galipeau J. Krampera M. Leblanc K. Martin I. . (2019). Mesenchymal stem versus stromal cells: International Society for Cell & Gene Therapy (ISCT®) Mesenchymal Stromal Cell committee position statement on nomenclature. Cytotherapy 21, 1019–1024. 10.1016/j.jcyt.2019.08.002 31526643

[B116] Wang S. Lu J. Li Y. A. Zhou H. Ni W. F. Zhang X. L. . (2016). Autologous olfactory lamina propria transplantation for chronic spinal cord injury: three-year follow-up outcomes from a prospective double-blinded clinical trial. Cell Transplant. 25, 141–157. 10.3727/096368915X688065 25924918

[B117] Wilson J. R. Forgione N. Fehlings M. G. (2013). Emerging therapies for acute traumatic spinal cord injury. CMAJ 185, 485–492. 10.1503/cmaj.121206 23228995 PMC3612151

[B118] Woodhall E. West A. K. Chuah M. I. (2001). Cultured olfactory ensheathing cells express nerve growth factor, brain-derived neurotrophic factor, glia cell line-derived neurotrophic factor and their receptors. Brain Res. Mol. Brain Res. 88, 203–213. 10.1016/S0169-328X(01)00044-4 11295250

[B119] Wu J. Sun T. Ye C. Yao J. Zhu B. He H. (2012). Clinical observation of fetal olfactory ensheathing glia transplantation (OEGT) in patients with complete chronic spinal cord injury. Cell Transplant. 21(Suppl. 1), S33–S37. 10.3727/096368912X633743 22507678

[B120] Wu S. Suzuki Y. Ejiri Y. Noda T. Bai H. Kitada M. . (2003). Bone marrow stromal cells enhance differentiation of cocultured neurosphere cells and promote regeneration of injured spinal cord. J. Neurosci. Res. 72, 343–351. 10.1002/jnr.10587 12692901

[B121] Xiao Z. Tang F. Zhao Y. Han G. Yin N. Li X. . (2018). Significant improvement of acute complete spinal cord injury patients diagnosed by a combined criteria implanted with neuroregen scaffolds and mesenchymal stem cells. Cell Transplant. 27, 907–915. 10.1177/0963689718766279 29871514 PMC6050906

[B122] Xu P. Yang X. (2019). The efficacy and safety of mesenchymal stem cell transplantation for spinal cord injury patients: a meta-analysis and systematic review. Cell Transplant. 28, 36–46. 10.1177/0963689718808471 30362373 PMC6322141

[B123] Yang Y. Pang M. Du C. Liu Z. Y. Chen Z. H. Wang N. X. . (2021). Repeated subarachnoid administrations of allogeneic human umbilical cord mesenchymal stem cells for spinal cord injury: a phase 1/2 pilot study. Cytotherapy 23, 57–64. 10.1016/j.jcyt.2020.09.012 33218835

[B124] Yazdani S. O. Hafizi M. Zali A. R. Atashi A. Ashrafi F. Seddighi A. S. . (2013). Safety and possible outcome assessment of autologous Schwann cell and bone marrow mesenchymal stromal cell co-transplantation for treatment of patients with chronic spinal cord injury. Cytotherapy 15, 782–791. 10.1016/j.jcyt.2013.03.012 23731761

[B125] Zachar L. Bačenková D. Rosocha J. (2016). Activation, homing, and role of the mesenchymal stem cells in the inflammatory environment. J. Inflamm. Res. 9, 231–240. 10.2147/JIR.S121994 28008279 PMC5170601

[B126] Zhou H. L. Zhang X. J. Zhang M. Y. Yan Z. J. Xu Z. M. Xu R. X. (2016). Transplantation of human amniotic mesenchymal stem cells promotes functional recovery in a rat model of traumatic spinal cord injury. Neurochem. Res. 41, 2708–2718. 10.1007/s11064-016-1987-9 27351200

