# Supplementary material for: Comparing the Efficacy and Safety of Cell Transplantation for Spinal Cord Injury: A Systematic Review and Bayesian Network Meta-Analysis
Source: Front Cell Neurosci. 2022 Apr 4;16:860131. doi: 10.3389/fncel.2022.860131 (PMC9013778; doi:10.3389/fncel.2022.860131)

Supplementary materials

**Contents.**

[Supplementary Figures and Tables 2](#_Toc98523543)

[Supplementary Tables 2](#_Toc98523544)

[Table S1. Checklist of the PRISMA extension for network meta-analysis. 2](#_Toc98523545)

[Table S2. Search strategy (PubMed) 6](#_Toc98523546)

[Table S3. ASIA IMPAIRMENT SCALE (AIS) SCORE 7](#_Toc98523547)

[Table S4. Characteristic of included studies. 8](#_Toc98523548)

[Table S5. Risk of bias within included studies 14](#_Toc98523549)

[Table S6. The value of surface under the cumulative ranking (SUCRA) for all outcome measures. 18](#_Toc98523550)

[Table S7. GRADE assessment for AIS grade at 6 months and 12 months 19](#_Toc98523551)

[Table S8. GRADE assessment for ASIA motor score at 6 months and 12 months 20](#_Toc98523552)

[Table S9. GRADE assessment for ASIA light touch score at 6 months and 12 months 21](#_Toc98523553)

[Table S10. GRADE assessment for ASIA pinprick score at 6 months and 12 months 22](#_Toc98523554)

[Table S11. GRADE assessment for FIM score at 6 months and 12 months 23](#_Toc98523555)

[Table S12. GRADE assessment for IANR-SCIFRS at 6 months and 12 months 24](#_Toc98523556)

[Table S13. Severe adverse event. 25](#_Toc98523557)

[Table S14. Pairwise result of different outcomes (comparing with baseline). 26](#_Toc98523558)

[Table S15. Network meta-regression (assessment of the transitivity). 28](#_Toc98523559)

[Table S16. Comparisons of the fit of consistency and inconsistency models. 30](#_Toc98523560)

[Supplementary Figures 31](#_Toc98523561)

[Figure S1. The funnel plot for small sample effect test for AIS grade 31](#_Toc98523562)

[Figure S2. The funnel plot for small sample effect test for ASIA motor score. 32](#_Toc98523563)

[Figure S3. The funnel plot for small sample effect test for ASIA light touch score 33](#_Toc98523564)

[Figure S4. The funnel plot for small sample effect test for ASIA pinprick score. 34](#_Toc98523565)

[Figure S5. Bean plot for mean age of included studies (assessment of the transitivity). 35](#_Toc98523566)

[Figure S6. Assessment of the aggregation for AIS grade at 6 months. 36](#_Toc98523567)

[Figure S7. Assessment of the aggregation for AIS grade at 12 months. 38](#_Toc98523568)

[Figure S8. Assessment of the aggregation for ASIA motor score at 6 months. 40](#_Toc98523569)

[Figure S9. Assessment of the aggregation for ASIA motor score at 12 months. 42](#_Toc98523570)

[Figure S10. Assessment of the aggregation for ASIA light touch score at 6 months. 44](#_Toc98523571)

[Figure S11. Assessment of the aggregation for ASIA light touch score at 12 months. 46](#_Toc98523572)

[Figure S12. Assessment of the aggregation for ASIA pinprick score at 6 months. 48](#_Toc98523573)

[Figure S13. Assessment of the aggregation for ASIA pinprick score at 12 months. 50](#_Toc98523574)

[Figure S14. Assessment of the aggregation for FIM score at 6 months. 52](#_Toc98523575)

[Figure S15. Assessment of the aggregation for FIM score at 12 months. 54](#_Toc98523576)

[Figure S16. Assessment of the aggregation for IANR-SCIFRS at 6 months. 56](#_Toc98523577)

[Figure S17. Assessment of the aggregation for IANR-SCIFRS at 12 months. 58](#_Toc98523578)

# Supplementary Figures and Tables

## Supplementary Tables

Table S1. Checklist of the PRISMA extension for network meta-analysis.

| **Section and Topic** | **Item #** | **Checklist item** | **Location where item is reported** |  |  |
| --- | --- | --- | --- | --- | --- |
| **TITLE** | | |  |  |  |
| Title | 1 | Identify the report as a systematic review. | 1 |  |  |
| **ABSTRACT** | | |  |  |  |
| Abstract | 2 | See the PRISMA 2020 for Abstracts checklist. | 1-2 |  |  |
| **INTRODUCTION** | | |  |  |  |
| Rationale | 3 | Describe the rationale for the review in the context of existing knowledge. | 2-4 |  |  |
| Objectives | 4 | Provide an explicit statement of the objective(s) or question(s) the review addresses. | 4 |  |  |
| **METHODS** | | |  |  |  |
| Eligibility criteria | 5 | Specify the inclusion and exclusion criteria for the review and how studies were grouped for the syntheses. | 4-5 |  |  |
| Information sources | 6 | Specify all databases, registers, websites, organisations, reference lists and other sources searched or consulted to identify studies. Specify the date when each source was last searched or consulted. | 4 |  |  |
| Search strategy | 7 | Present the full search strategies for all databases, registers and websites, including any filters and limits used. | Supplementary Table S2 |  |  |
| Selection process | 8 | Specify the methods used to decide whether a study met the inclusion criteria of the review, including how many reviewers screened each record and each report retrieved, whether they worked independently, and if applicable, details of automation tools used in the process. | 4-5 |  |  |
| Data collection process | 9 | Specify the methods used to collect data from reports, including how many reviewers collected data from each report, whether they worked independently, any processes for obtaining or confirming data from study investigators, and if applicable, details of automation tools used in the process. | 5 |  |  |
| Data items | 10a | List and define all outcomes for which data were sought. Specify whether all results that were compatible with each outcome domain in each study were sought (e.g. for all measures, time points, analyses), and if not, the methods used to decide which results to collect. | 5 |  |  |
|  | 10b | List and define all other variables for which data were sought (e.g. participant and intervention characteristics, funding sources). Describe any assumptions made about any missing or unclear information. | 4-7, 14-15 |  |  |
| Study risk of bias assessment | 11 | Specify the methods used to assess risk of bias in the included studies, including details of the tool(s) used, how many reviewers assessed each study and whether they worked independently, and if applicable, details of automation tools used in the process. | 5 |  |  |
| Effect measures | 12 | Specify for each outcome the effect measure(s) (e.g. risk ratio, mean difference) used in the synthesis or presentation of results. | 6 |  |  |
| Synthesis methods | 13a | Describe the processes used to decide which studies were eligible for each synthesis (e.g. tabulating the study intervention characteristics and comparing against the planned groups for each synthesis (item #5)). | 6 |  |  |
|  | 13b | Describe any methods required to prepare the data for presentation or synthesis, such as handling of missing summary statistics, or data conversions. | 6 |  |  |
|  | 13c | Describe any methods used to tabulate or visually display results of individual studies and syntheses. | 6 |  |  |
|  | 13d | Describe any methods used to synthesize results and provide a rationale for the choice(s). If meta-analysis was performed, describe the model(s), method(s) to identify the presence and extent of statistical heterogeneity, and software package(s) used. | 6 |  |  |
|  | 13e | Describe any methods used to explore possible causes of heterogeneity among study results (e.g. subgroup analysis, meta-regression). | 6 |  |  |
|  | 13f | Describe any sensitivity analyses conducted to assess robustness of the synthesized results. | 6 |  |  |
| Reporting bias assessment | 14 | Describe any methods used to assess risk of bias due to missing results in a synthesis (arising from reporting biases). | 6-7 |  |  |
| Certainty assessment | 15 | Describe any methods used to assess certainty (or confidence) in the body of evidence for an outcome. | 6-7 |  |  |
| **RESULTS** | | |  |  |  |
| Study selection | 16a | Describe the results of the search and selection process, from the number of records identified in the search to the number of studies included in the review, ideally using a flow diagram. | 7-8 |  |  |
|  | 16b | Cite studies that might appear to meet the inclusion criteria, but which were excluded, and explain why they were excluded. | 7-8 |  |  |
| Study characteristics | 17 | Cite each included study and present its characteristics. | 8, Supplementary Table S4 |  |  |
| Risk of bias in studies | 18 | Present assessments of risk of bias for each included study. | 8, Supplementary Table S5. |  |  |
| Results of individual studies | 19 | For all outcomes, present, for each study: (a) summary statistics for each group (where appropriate) and (b) an effect estimate and its precision (e.g. confidence/credible interval), ideally using structured tables or plots. | 10-11, Supplementary Table S14. |  |  |
| Results of syntheses | 20a | For each synthesis, briefly summarise the characteristics and risk of bias among contributing studies. | 8-10 |  |  |
|  | 20b | Present results of all statistical syntheses conducted. If meta-analysis was done, present for each the summary estimate and its precision (e.g. confidence/credible interval) and measures of statistical heterogeneity. If comparing groups, describe the direction of the effect. | 8-10,  Table 2 |  |  |
|  | 20c | Present results of all investigations of possible causes of heterogeneity among study results. | 11 |  |  |
|  | 20d | Present results of all sensitivity analyses conducted to assess the robustness of the synthesized results. | 11, Supplementary Figure S1-4, Table S15 S16. |  |  |
| Reporting biases | 21 | Present assessments of risk of bias due to missing results (arising from reporting biases) for each synthesis assessed. | 11, Supplementary Figure S1-4. |  |  |
| Certainty of evidence | 22 | Present assessments of certainty (or confidence) in the body of evidence for each outcome assessed. | 10, Supplementary Table S7-S12 |  |  |
| **DISCUSSION** | | |  |  |  |
| Discussion | 23a | Provide a general interpretation of the results in the context of other evidence. | 11-14 |  |  |
|  | 23b | Discuss any limitations of the evidence included in the review. | 14 |  |  |
|  | 23c | Discuss any limitations of the review processes used. | 14 |  |  |
|  | 23d | Discuss implications of the results for practice, policy, and future research. | 14 |  |  |
| **OTHER INFORMATION** | | |  |  |  |
| Registration and protocol | 24a | Provide registration information for the review, including register name and registration number, or state that the review was not registered. | 2, 4 |  |  |
|  | 24b | Indicate where the review protocol can be accessed, or state that a protocol was not prepared. | 2, 4 |  |  |
|  | 24c | Describe and explain any amendments to information provided at registration or in the protocol. | 2, 4 |  |  |
| Support | 25 | Describe sources of financial or non-financial support for the review, and the role of the funders or sponsors in the review. | 14-15 |  |  |
| Competing interests | 26 | Declare any competing interests of review authors. | 14 |  |  |
| Availability of data, code and other materials | 27 | Report which of the following are publicly available and where they can be found: template data collection forms; data extracted from included studies; data used for all analyses; analytic code; any other materials used in the review. | 14-15 |  |  |

*From:*  Page MJ, McKenzie JE, Bossuyt PM, Boutron I, Hoffmann TC, Mulrow CD, et al. The PRISMA 2020 statement: an updated guideline for reporting systematic reviews. BMJ 2021;372:n71. doi: 10.1136/bmj.n71

For more information, visit: <http://www.prisma-statement.org/>

Table S2. Search strategy (PubMed)

(((((((((((((neural stem cells[MeSH Terms]) OR (neural stem cells[Title/Abstract])) OR (neural stem cell[Title/Abstract])) OR (neural progenitor cell[Title/Abstract])) OR (neural progenitor cells[Title/Abstract])) OR (((oligodendrocyte precursor cells[MeSH Terms]) OR (oligodendrocyte precursor cells[Title/Abstract])) OR (oligodendrocyte precursor cell[Title/Abstract]))) OR (((schwann cells[MeSH Terms]) OR (schwann cells[Title/Abstract])) OR (schwann cell[Title/Abstract]))) OR ((olfactory ensheathing cells[Title/Abstract]) OR (olfactory ensheathing cell[Title/Abstract]))) OR (((mesenchymal stem cells[MeSH Terms]) OR (mesenchymal stem cells[Title/Abstract])) OR (mesenchymal stem cell[Title/Abstract]))) OR ((((((Cell- and Tissue-Based Therapy[MeSH Terms]) OR (cell therapy[Title/Abstract])) OR (cell transplantation[Title/Abstract])) OR (transplantation[Title/Abstract])) OR (graft*[Title/Abstract])) OR (transplant*[Title/Abstract]))) OR (((embryonic stem cells[MeSH Terms]) OR (embryonic stem cells[Title/Abstract])) OR (embryonic stem cell[Title/Abstract]))) OR (perinatal stem cell*[Title/Abstract])) OR (((induced pluripotent stem cells[MeSH Terms]) OR (induced pluripotent stem cells[Title/Abstract])) OR (induced pluripotent stem cell[Title/Abstract]))) AND ((((((spinal cord injuries[MeSH Terms]) OR (spinal cord ischemia[MeSH Terms])) OR (spinal cord injuries[Title/Abstract])) OR (spinal cord injury[Title/Abstract])) OR (spinal cord ischemia[Title/Abstract])) OR (spinal cord ischemias[Title/Abstract]))

Table S3. ASIA IMPAIRMENT SCALE (AIS) SCORE

**Grade A:** The impairment is complete. There is no motor or sensory function left below the level of injury.
**Grade B:** The impairment is incomplete. Sensory function, but not motor function, is preserved below the neurologic level (the first normal level above the level of injury) and some sensation is preserved in the sacral segments S4 and S5.
**Grade C:**The impairment is incomplete. Motor function is preserved below the neurologic level, but more than half of the key muscles below the neurologic level have a muscle grade less than 3 (i.e., they are not strong enough to move against gravity).
**Grade D:** The impairment is incomplete. Motor function is preserved below the neurologic level, and at least half of the key muscles below the neurologic level have a muscle grade of 3 or more (i.e., the joints can be moved against gravity).
**Grade E:**The patient's functions are normal. All motor and sensory functions are unhindered

Table S4. Characteristic of included studies.

| Authors | Year | Country | NCT | Study type | cell | Sample size | Injury level (C/T/L) | M/F | Age (years) | ASIA(A/B/C/D) | Duration | Cell source | Way of transplantation | Follow up (months) | Outcomes | Results |
| --- | --- | --- | --- | --- | --- | --- | --- | --- | --- | --- | --- | --- | --- | --- | --- | --- |
| Anderson | 2017 | the Unite States | NCT01739023 | case series | Schwann | 6 | T | 6/0 | 25-41 | A | 29-59d | auto | intraspinal injection | 12 | ①②③④⑤⑦ | FIM score improved at 12 months; no significant improved in  AIS grade, motor or sensory function; 1 SAE happened. |
| Saberi | 2008 | Iran | - | case series | Schwann | 4 | T | 3/1 | 22-43 | 2/0/2 | 26-80m | auto | intraspinal injection | 12 | ①⑦ | AIS grade improved at 12 months; no SAE happened. |
| Gant | 2021 | the Unite States | NCT02354625 | case series | Schwann | 8 | 4/4 | 5/3 | 20-52 | 4/2/2 | 1-15y | auto | intraspinal injection | 6 | ①②③④⑦ | Motor and sensory function improved at 6 months; AIS grade didn't improve; no SAE happened. |
| Chen | 2014 | China | - | RCT | OEC | 3 | C | 2/1 | 22-36 | A | 69-165m | allo | intraspinal injection | 6 | ②③④⑥⑦ | Motor and sensory function improved at 6 months; no SAE happened. |
| Chhabra | 2009 | India | - | case series | OEC | 5 | 1/4 | 5/0 | 21-38 | 4/1 | 29-99m | auto | intraspinal injection | 28 | ②③④ | A patient with AIS grade B decresed sensory function. |
| Feron | 2005 | Australia | - | case control | OEC | 3 | T | 3/0 | 23-46 | A | 18-32m | auto | intraspinal injection | 12 | ⑦ | No AE happened. |
| Huang | 2006 | China | - | case serious | OEC | 16 | 10/6/0 | 14/2 | 22-55 | - | 18-96m | allo | intraspinal injection | 38 | ⑦ | No AE happened. |
| Huang | 2009 | China | ‘- | case  control | OEC | 566 | - | 463/103 | 1-73 | - | 4-372m | allo | intraspinal injection | 12-63 | ②③④⑦ | Motor and sensory function improved at 6 months; 38 SAE happened; 2 patients died. |
| Huang | 2012 | China | - | case control | OEC | 108 | 51/42/15(T12-L1) | 84/24 | 6-58 | - | 6-360m | allo | intraspinal injection | 12-64 | ②③④⑥⑦ | All outcome measures improved at 12 months; no AE happened. |
| IWATSUKI | 2016 | Japan | - | case serious | OEC | 8 | T | 6/2 | 19-40 | 7/1 | 17-297m | auto | intraspinal injection | 24 | ①②③④⑦ | AIS grade and motor function improved; sensory didn't improved at 12 months; no AE happened. |
| Lima | 2010 | Portugal | - | case serious | OEC | 20 | C4-T12 | 17/3 | 19-37 | 15/5 | 18-189m | auto | intraspinal injection | 12-45 | ①②③④⑤⑦ | AIS grade, motor and sensory function improved at 12 months; 1 SAE happened. |
| Lima | 2006 | Portugal | - | case serious | OEC | 7 |  | 4/3 | 18-32 | A | 6-78m | auto | intraspinal injection | 18 | ①②③④⑦ | AIS grade, motor and sensory function improved at 12 months; no SAE happened. |
| Mackay-Sim | 2008 | Australia | - | case control | OEC | 3 | T | 3/0 | 23-46 | A | 18-32m | auto | intraspinal injection | 36 | ①②③④⑤ | Sensory function improved at 12 months; no AE happened. |
| Rao | 2013 | China | - | case serious | OEC | 6 |  | 4/2 | 30-56 | A | 18.83±5.78m | allo | intraspinal injection | 24 | ②③④⑥⑦ | Motor and sensory function improved at 12 months; no SAE happened. |
| Rao | 2013 | China | - | case serious | OEC | 8 |  | 5/3 | 18-50 | 3/5 | 8-15m | auto | intrathecal injection | 24 | ①⑥ | AIS grade improved at 12 months; no SAE happened. |
| Tabakow | 2013 | Poland | - | case control | OEC | 3 | T | 3/0 | 24-26 | A | 1.3-4 | auto | intraspinal injection | 12 | ①⑤⑦ | AIS grade improved at 12 months; no SAE happened. |
| Wang | 2016 | China | - | RCT | OEC | 8 | T | 7/1 | 35-45 | A | 6-60 | auto | intraspinal injection | 36 | ①②③④⑤⑥ | No improvement in motor and sensory function; 1 SAE happened. |
| Wu | 2012 | China | - | case serious | OEC | 11 | 5/6 | 9/2 | 25-55 | A | 24-66m | allo | intraspinal injection | 12-18 | ②③④⑥⑦ | Motor and sensory function improved at 12 months; no SAE happened. |
| Curt | 2020 | Switzerland | - | case series | NSC | 12 | T | 11/1 | 19-53 | 7/5 | 4-24m | allo | intraspinal injection | 72 | ①⑦ | AIS grade improved at 12 months; 4 SAE happened. |
| Curtis | 2018 | the United State | NCT 01772810 | case series | NSC | 4 | T | 3/1 | 24-35 | A | 175-553d | allo | intraspinal injection | 18-27 | ⑦ | No AE happened. |
| Levi | 2019 | the Unite States | NCT02163876 | RCT | NSC | 12 | C | 11/1 | 22-48 | 3/9 | 0.4-1.8y | allo | intraspinal injection | 12 | ①⑦ | AIS grade didn't improve; 9 SAE happened. |
| Levi | 2018 | the Unite States and Canada |  | case series | NSC | 12 | T | 11/1 | 19-53 | 8/4 | 25-57mo | allo | intraspinal injection | 25-57 | ⑦ | 26 SAE happened. |
| Shin | 2015 | Republic of Korea | KCT0000879;Clinical Research Information Service (CRIS) | case control | NSC | 19 | C | 16/3 | 18-57 | 17/2 | 16-213d | allo | intraspinal injection | 12 | ①②③④⑦ | AIS grade, motor and sensory function improved at 12 months; no SAE happened. |
| Ghobrial | 2017 | the Unite states | NCT01321333、NCT02163876 | case series | NSC | 4 | C | 5/0 | 24-49 | 1/4 | 7-12 | allo | intraspinal injection | 12 | ①②③④⑦ | Motor and sensory function improved at 12 months; no SAE happened. |
| Cheng | 2014 | China | NCT01393977 | case control | MSC | 10 | thoracolumbar | - | 35.30±8.23 | A | 12-50m | allo | intraspinal injection | 6 | ②⑦ | Motor function improved at 6 months; no SAE happened. |
| Liu | 2013 | China | - | case series | MSC | 22 | 6/9/7 | 17/5 | 18-51 | 6/16 | 2-204mo | allo | intrathecal injection | 36 | ⑦ | No SAE happened. |
| Yang | 2021 | China | NCT02481440 | case series | MSC | 102 | 24/17 | 33/8 | 18-65 | NA | 2-240m | allo | intrathecal injection | 12 | ②③④⑥⑦ | Motor and sensory function improved at 12 months; no SAE happened. |
| Bhanot | 2011 | India | - | case series | MSC | 13 | 5/8 | 10/3 | 18-52 | A | 3-132m | auto | intraspinal injection and intrathecal injection | 6-38(13.5) | ①⑦ | AIS grade imprived at 12 months; no SAE happened. |
| El-Kheir | 2014 | Egypt | NCT00816803 | RCT | MSC | 50 | 10/40 | 61/9(including two groups) | 16-45 | 15/35 | 12-36m | auto | intrathecal injection | 18 | ①②③④⑦ | AIS grade, motor and sensory function improved; no SAE happened. |
| Kishk | 2010 | Egypt | - | case control | MSC | 43 | 6/37 | 36/7 | 31.7±10.4 | 40/0/3 | 3.6+-2.5y | auto | intrathecal injection | 12 | ①②③④⑦ | AIS grade, motor and sensory function improved at 12 months; 1 SAE happened. |
| Larocca | 2017 | Brazil | NCT02152657 | case series | MSC | 5 | T | 5/0 | 36-52 | A | 25-111m | auto | intraspinal injection | 6 | ①⑤⑦ | AIS grade improved at 6 months; no SAE happened. |
| Mendonça | 2014 | Brazil | NCT01325103 | case series | MSC | 14 | 0/13/1 | 10/4 | 23-61 | A | 18-180m | auto | intraspinal injection | 6 | ①②③④⑦ | AIS grade, motor ande sensory function improved at 6 months; 1 SAE happened. |
| Pal | 2009 | India | - | case series | MSC | 30 | 7/23 | 27/3 | 17-55 | NA | >6mo | auto | intrathecal injection | 12 | ⑦ | No AE happened. |
| Park | 2012 | Republic of Korea |  | case series | MSC | 10 | C | 8/2 | 34-61 | 4/6 | 1-108m | auto | intraspinal injection | 6 | ⑦ | No SAE happened. |
| Saito | 2012 | Japan | NCT00695149 | case series | MSC | 5 | C | 5/0 | 23-59 | 3/1/1 | 8-17d | auto | intrathecal injection | 6 | ①②③④⑦ | AIS grade improved at 6 months; no SAE happened. |
| Satti | 2016 | Pakistan | NCT02482194 | case control | MSC | 9 | T | 9/0 | 26-38 | A | 10-55m | auto | intrathecal injection | 630-826d | ⑦ | No SAE happened. |
| Vaquero | 2017 | Spain | NCT02165904 | csee series | MSC | 10 | 5/2'3 | 8/2 | 34-59 | 0/4/5/1 | 2.43-34.59y | auto | intrathecal injection | 12 | ②③④⑤⑥⑦ | Motor and sensory function improved at 12 months; no SAE happened. |
| Vaquero | 2018 | Spain | NCT02807142 | case series | MSC | 6 | 0/5/1 | 6/0 | 30-50 | 3/2/0/1 | 5.75-27.68y | auto | intraspinal injection | 6 | ③④⑥⑦ | Sensory function improved at 6 months; no AE happened. |
| Vaquero | 2016 | Spain | NCT01909154 | case series | MSC | 12 | 1/11 | 9/3 | 25-58 | A | 3.17-26.75y | auto | intraspinal injection; intrathecal injection | 12 | ①②③④⑤⑥⑦ | AIS grade, motor and sensory function improved at 12 months; no SAE happened. |
| Hur | 2016 | Republic of Korea | Investigational New Drug Application no. 201005033(the Korean Food and Drug Administration) | case series | MSC | 14 | 6/7/1 | 12/2 | 20-66 | 12/1/0/1 | 3-28m | auto | intrathecal injection | 8 | ②③④⑦ | Motor and sensory function improved; no SAE happened. |
| Knoller | 2005 | Israel | - | case series | Macrophage | 8 | 1/7 | 7/1 | 19-41 | A | 9-14days | auto | intraspinal injection | 12 | ①②③④⑦ | AIS grade, motor and sensory function improved at 12 months; 3 SAE happened. |
| Lammertse | 2012 | the United States and Israel | NCT00073853 | RCT | Macrophage | 33 | 15/18 | 27/6 | 27.4±11.0 | A | <14days | auto | intraspinal injection | 12 | ①②③④⑤⑦ | AIS grade, motor and sensory function improved at 12 months; 9 SAE happened;1 patient died. |
| Oraee-Yazdani | 2016 | Iran | - | case series | Combined | 6 | 1/5 | 5/2 | 22-45 | A | 38.1+-15.28m | auto | intrathecal injection | 25-36 | ②⑦ | Motor function didn't improved; no SAE happened. |
| Yazdani | 2013 | Iran | - | case series | Combined | 8 | 1/7 | 4/4 | 15-45 | A | 13-63m | auto | intraspinal injection | 24 | ①②⑦ | AIS grade and motor function didn't improved; no SAE happened. |
|  |  |  |  |  |  |  |  |  |  |  |  |  |  |  |  |  |

1. American Spinal Cord Injury Association (ASIA) Impairment Scale (AIS grade)

② ASIA motor score

③ ASIA light touch score

④ ASIA pinprick score

⑤ FIM score: functional independence measure

⑥ IANR-SCIFRS: International association of Neurorestoratology Spinal Cord Injury Functional Rating Scale

⑦ adverse events

NCT: The identified number that studies registered in the ClinicalTrials.gov (<https://clinicaltrials.gov/ct2/home>).

SAE, severe adverse event; AE, adverse event; MSC, mesenchymal stem cells; NSC, neural stem cell/neural progenitor cell; OEC, olfactory ensheathing cells

Table S5. Risk of bias within included studies

1. **Risk of bias for case series**

| case series | 1 | 2 | 3 | 4 | 5 | 6 | 7 | 8 | 9 | 10 | Overall appraisal |
| --- | --- | --- | --- | --- | --- | --- | --- | --- | --- | --- | --- |
| Anderson 2017 | YES | N/A | YES | Unclear | YES | YES | YES | YES | YES | YES | Include |
| Saberi 2008 | YES | N/A | YES | Unclear | NO | YES | YES | YES | YES | YES | Include |
| Gant 2021 | YES | N/A | YES | YES | YES | YES | YES | YES | YES | YES | Include |
| Chhabra 2009 | YES | N/A | YES | Unclear | Unclear | YES | YES | YES | YES | YES | Include |
| Huang 2006 | Unclear | N/A | YES | Unclear | YES | YES | YES | YES | YES | YES | Include |
| IWATSUKI 2016 | YES | N/A | YES | Unclear | Unclear | YES | YES | YES | YES | YES | Include |
| Lima 2010 | YES | N/A | YES | Unclear | Unclear | YES | YES | YES | YES | YES | Include |
| Lima 2006 | YES | N/A | YES | Unclear | Unclear | YES | YES | YES | YES | YES | Include |
| Rao 2013 | YES | N/A | YES | Unclear | Unclear | YES | YES | YES | YES | YES | Include |
| Rao 2013 | YES | N/A | YES | Unclear | Unclear | YES | YES | YES | YES | YES | Include |
| Wu 2012 | YES | N/A | YES | Unclear | Unclear | YES | YES | YES | YES | YES | Include |
| Curt 2020 | Unclear | N/A | YES | Unclear | Unclear | YES | YES | YES | YES | YES | Include |
| Curtis 2018 | YES | N/A | YES | Unclear | NO | YES | YES | YES | YES | YES | Include |
| Levi 2018 | YES | N/A | YES | Unclear | YES | YES | YES | YES | YES | YES | Include |
| Liu 2013 | Unclear | N/A | YES | Unclear | Unclear | YES | YES | YES | YES | YES | Include |
| Ghobrial 2017 | YES | N/A | YES | Unclear | NO | YES | YES | YES | YES | YES | Include |
| Yang 2021 | YES | N/A | YES | YES | YES | YES | YES | YES | YES | YES | Include |
| Bhanot 2011 | YES | N/A | YES | Unclear | Unclear | YES | YES | YES | YES | YES | Include |
| Larocca 2017 | YES | N/A | YES | Unclear | YES | YES | YES | YES | YES | YES | Include |
| Mendonça 2014 | YES | N/A | YES | Unclear | YES | YES | YES | YES | YES | YES | Include |
| Pal 2009 | YES | N/A | YES | Unclear | Unclear | YES | YES | YES | YES | YES | Include |
| Park 2012 | Unclear | N/A | YES | Unclear | Unclear | YES | YES | YES | YES | YES | Include |
| Saito 2012 | YES | N/A | YES | Unclear | Unclear | YES | YES | YES | YES | YES | Include |
| Vaquero 2017 | YES | N/A | YES | Unclear | YES | YES | YES | YES | YES | YES | Include |
| Vaquero 2018 | YES | N/A | YES | Unclear | YES | YES | YES | YES | YES | YES | Include |
| Vaquero 2016 | YES | N/A | YES | Unclear | YES | YES | YES | YES | YES | YES | Include |
| Hur 2016 | YES | N/A | YES | Unclear | YES | YES | YES | YES | YES | YES | Include |
| Knoller 2005 | YES | N/A | YES | Unclear | Unclear | YES | YES | YES | YES | YES | Include |
| Oraee-Yazdani 2016 | YES | N/A | YES | Unclear | Unclear | YES | YES | YES | YES | YES | Include |
| Yazdani 2013 | YES | N/A | YES | Unclear | Unclear | YES | YES | YES | YES | YES | Include |

1. Were there clear criteria for inclusion in the case series?

2. Was the condition measured in a standard, reliable way for all participants included in the case series?

3. Were valid methods used for identification of the condition for all participants included in the case series?

4. Did the case series have consecutive inclusion of participants?

5. Did the case series have complete inclusion of participants?

6. Was there clear reporting of the demographics of the participants in the study?

7. Was there clear reporting of clinical information of the participants?

8. Were the outcomes or follow up results of cases clearly reported?

9. Was there clear reporting of the presenting site(s)/clinic(s) demographic information?

10. Was statistical analysis appropriate?

1. **Risk of bias for case-control trials**

| Case-control | 1 | 2 | 3 | 4 | 5 | 6 | 7 | 8 | 9 | 10 | Overall appraisal |
| --- | --- | --- | --- | --- | --- | --- | --- | --- | --- | --- | --- |
| Feron 2005 | Unclear | YES | YES | N/A | N/A | YES | YES | YES | YES | YES | Include |
| Mackay-Sim 2008 | Unclear | YES | YES | N/A | N/A | YES | YES | YES | YES | YES | Include |
| Huang 2012 | Unclear | YES | YES | N/A | N/A | YES | YES | YES | YES | YES | Include |
| Shin 2015 | YES | YES | YES | N/A | N/A | YES | YES | YES | YES | YES | Include |
| Kishk 2010 | YES | YES | YES | N/A | N/A | YES | YES | YES | YES | YES | Include |
| Satti 2016 | YES | YES | YES | N/A | N/A | YES | YES | YES | YES | YES | Include |
| Cheng 2014 | YES | YES | YES | N/A | N/A | YES | YES | YES | YES | YES | Include |

1. Were the groups comparable other than the presence of disease in cases or the absence of disease in controls?

2. Were cases and controls matched appropriately?

3. Were the same criteria used for identification of cases and controls?

4. Was exposure measured in a standard, valid and reliable way?

5. Was exposure measured in the same way for cases and controls?

6. Were confounding factors identified?

7. Were strategies to deal with confounding factors stated?

8. Were outcomes assessed in a standard, valid and reliable way for cases and controls?

9. Was the exposure period of interest long enough to be meaningful?

10. Was appropriate statistical analysis used

1. **Risk of bias for randomized controlled trials.**

| RCT | 1 | 2 | 3 | 4 | 5 | 6 | 7 | 8 | 9 | 10 | 11 | 12 | 13 | Overall appraisal |
| --- | --- | --- | --- | --- | --- | --- | --- | --- | --- | --- | --- | --- | --- | --- |
| Chen 2014 | Unclear | Unclear | YES | Unclear | Unclear | Unclear | YES | YES | YES | YES | YES | YES | YES | Include |
| Wang 2016 | YES | YES | YES | YES | YES | YES | YES | YES | YES | YES | YES | YES | YES | Include |
| Levi 2019 | YES | YES | YES | YES | YES | YES | YES | NO | YES | YES | YES | YES | YES | Include |
| El-Kheir 2014 | YES | YES | YES | YES | YES | YES | YES | YES | YES | YES | YES | YES | YES | Include |
| Lammertse 2012 | YES | YES | YES | YES | YES | YES | YES | YES | YES | YES | YES | YES | YES | Include |

1. Was true randomization used for assignment of participants to treatment groups?

2. Was allocation to treatment groups concealed?

3. Were treatment groups similar at the baseline?

4. Were participants blind to treatment assignment?

5. Were those delivering treatment blind to treatment assignment?

6. Were outcomes assessors blind to treatment assignment?

7. Were treatment groups treated identically other than the intervention of interest?

8. Was follow up complete and if not, were differences between groups in terms of their follow up adequately described and analyzed?

9. Were participants analyzed in the groups to which they were randomized?

10. Were outcomes measured in the same way for treatment groups?

11. Were outcomes measured in a reliable way?

12. Was appropriate statistical analysis used?

13. Was the trial design appropriate, and any deviations from the standard RCT design (individual randomization, parallel groups) accounted for in the conduct and analysis of the trial?

Table S6. The value of surface under the cumulative ranking (SUCRA) for all outcome measures.

| **Cell** | **Baseline** | **Combined** | **Macrophage** | **MSC** | **NSC** | **OEC** | **Schwann** |
| --- | --- | --- | --- | --- | --- | --- | --- |
| **6mo AIS** | **23.50%** | **26.27%** | **85.76%** | **88.04%** | **40.66%** | **36.86%** | **48.90%** |
| **12mo AIS** | **16.39%** | **21.22%** | **80.92%** | **57.16%** | **45.86%** | **80.58%** | **47.87%** |
| **6mo motor** | **26.29%** | **33.03%** | **56.47%** | **70.60%** | **83.19%** | **36.85%** | **43.57%** |
| **12mo motor** | **19.54%** | **34.52%** | **74.79%** | **62.41%** | **75.29%** | **33.46%** | **-** |
| **6mo light touch** | **21.38%** | **-** | **84.10%** | **76.11%** | **51.88%** | **34.13%** | **32.39%** |
| **12mo light touch** | **6.83%** | **-** | **80.71%** | **74.57%** | **52.19%** | **35.70%** | **-** |
| **6mo pinprick** | **19.21%** | **-** | **76.41%** | **88.99%** | **40.15%** | **38.89%** | **36.35%** |
| **12mo pinprick** | **4.53%** | **-** | **83.97%** | **78.51%** | **51.62%** | **31.38%** | **-** |
| **6mo FIM score** | **20.76%** | **-** | **-** | **45.49%** | **-** | **83.75%** | **-** |
| **12mo FIM score** | **14.78%** | **-** | **86.29%** | **22.18%** | **-** | **53.90%** | **72.86%** |
| **6mo IANR-SCIFRS** | **8.37%** | **-** | **-** | **66.99%** | **-** | **74.65%** | **-** |
| **12mo IANR-SCIFRS** | **2.01%** | **-** | **-** | **96.84%** | **-** | **51.15%** | **-** |

Table S7. GRADE assessment for AIS grade at 6 months and 12 months

1. **6mo AIS grade**

| Certainty of the evidence, and classification of intervention | Intervention | Intervention v Baseline (mean difference (95% credible interval)) | Surface under the cumulative ranking curve |
| --- | --- | --- | --- |
| High certainty (moderate to high certainty evidence) | | | |
| Category 2: among the most effective | - | - | - |
| Category 1: inferior to the most effective, or superior to the least effective | MSC (M) | **0.42(0.15, 0.73)** | 88.04% |
| Category 0: among the least effective | OEC (M) | 0.06(-0.25, 0.41) | 36.86% |
|  | NSC (M) | 0.08(-0.22, 0.38) | 40.66% |
| Low certainty (low to very low certainty evidence) | | | |
| Category 2: might be among the most effective | - | - | - |
| Category 1: might be inferior to the most effective or superior than the least effective | Macrophage (L) | **0.41(0.06, 0.8)** | 85.76% |
| Category 0: might be among the least effective | Combined (VL) | 0(-0.39, 0.38) | 26.27% |
|  | Schwann (L) | 0.13(-0.29, 0.54) | 48.90% |

**B. 12mo AIS grade**

| Certainty of the evidence, and classification of intervention | Intervention | Intervention v Baseline (mean difference (95% credible interval)) | Surface under the cumulative ranking curve |
| --- | --- | --- | --- |
| High certainty (moderate to high certainty evidence) | | | |
| Category 2: among the most effective | - | - | - |
| Category 1: inferior to the most effective, or superior to the least effective | - | - | - |
| Category 0: among the least effective | MSC (M) | 0.18(-0.15, 0.56) | 57.16% |
|  | OEC (M) | 0.44(-0.04, 1.01) | 80.58% |
|  | NSC (M) | 0.15(-0.17, 0.49) | 45.86% |
| Low certainty (low to very low certainty evidence) | | | |
| Category 2: might be among the most effective | - | - | - |
| Category 1: might be inferior to the most effective or superior than the least effective | Macrophage (L) | **0.42(0, 0.91)** | 80.92% |
| Category 0: might be among the least effective | Combined (VL) | 0(-0.49, 0.49) | 16.39% |
|  | Schwann (L) | 0.18(-0.35, 0.72) | 47.87% |

Table S8. GRADE assessment for ASIA motor score at 6 months and 12 months

1. **6mo motor score**

| Certainty of the evidence, and classification of intervention | Intervention | Intervention v Baseline (mean difference (95% credible interval)) | Surface under the cumulative ranking curve |
| --- | --- | --- | --- |
| High certainty (moderate to high certainty evidence) | | | |
| Category 2: among the most effective | - | - | - |
| Category 1: inferior to the most effective, or superior to the least effective | MSC (M) | **4.43(0.91, 7.78)** | 70.60% |
| Category 0: among the least effective | OEC (M) | 0.58(-1.53, 3.3) | 36.85% |
| Low certainty (low to very low certainty evidence) | | | |
| Category 2: might be among the most effective | - | - | - |
| Category 1: might be inferior to the most effective or superior than the least effective | - | - | - |
| Category 0: might be among the least effective | Combined (VL) | 0.09(-8.1, 8.92) | 33.03% |
|  | Macrophage (VL) | 3.16(-7.29, 13.53) | 56.47% |
|  | NSC (VL) | 9.12(-2.61, 20.91) | 83.19% |
|  | Schwann (VL) | 1.13(-13.87, 16.17) | 43.57% |

1. **12mo motor score**

| Certainty of the evidence, and classification of intervention | Intervention | Intervention v Baseline (mean difference (95% credible interval)) | Surface under the cumulative ranking curve |
| --- | --- | --- | --- |
| High certainty (moderate to high certainty evidence) | | | |
| Category 2: among the most effective | - | - | - |
| Category 1: inferior to the most effective, or superior to the least effective | - | - | - |
| Category 0: among the least effective | MSC (M) | 2.37(-0.7, 5.25) | 62.41% |
|  | OEC (M) | 0.62(-0.48, 2.74) | 33.46% |
| Low certainty (low to very low certainty evidence) | | | |
| Category 2: might be among the most effective | - | - | - |
| Category 1: might be inferior to the most effective or superior than the least effective | - | - | - |
| Category 0: might be among the least effective | Combined (L) | 0.16(-8.11, 8.05) | 34.52% |
|  | Macrophage (VL) | 6.02(-5.09, 16.57) | 74.79% |
|  | NSC (L) | 4.17(-0.68, 9.46) | 75.29% |

Table S9. GRADE assessment for ASIA light touch score at 6 months and 12 months

1. **6mo light touch score**

| Certainty of the evidence, and classification of intervention | Intervention | Intervention v Baseline (mean difference (95% credible interval)) | Surface under the cumulative ranking curve |
| --- | --- | --- | --- |
| High certainty (moderate to high certainty evidence) | | | |
| Category 2: among the most effective | - | - | - |
| Category 1: inferior to the most effective, or superior to the least effective | MSC (M) | **10.01(5.81, 13.88)** | 76.11% |
| Category 0: among the least effective | OEC (M) | 2.03(-4.83, 8.96) | 34.13% |
| Low certainty (low to very low certainty evidence) | | | |
| Category 2: might be among the most effective | - | - | - |
| Category 1: might be inferior to the most effective or superior than the least effective | Macrophage (VL) | **13.07(1.03, 25.25)** | 84.10% |
| Category 0: might be among the least effective | NSC (VL) | 5.51(-7.53, 18.8) | 51.88% |
|  | Schwann (VL) | 0.11(-24.88, 24.09) | 32.39% |

1. **12mo light touch score**

| Certainty of the evidence, and classification of intervention | Intervention | Intervention v Baseline (mean difference (95% credible interval)) | Surface under the cumulative ranking curve |
| --- | --- | --- | --- |
| High certainty (moderate to high certainty evidence) | | | |
| Category 2: among the most effective | MSC (M) | **11.48(6.31, 16.64)** | 74.57% |
| Category 1: inferior to the most effective, or superior to the least effective | - | - | - |
| Category 0: among the least effective | OEC (M) | 3.71(-0.4, 8.24) | 35.70% |
| Low certainty (low to very low certainty evidence) | | | |
| Category 2: might be among the most effective | - | - | - |
| Category 1: might be inferior to the most effective or superior than the least effective | Macrophage (VL) | **14.35(1.02, 27.98)** | 80.71% |
| Category 0: might be among the least effective | NSC (L) | 7.77(-6.31, 21.79) | 52.19% |

Table S10. GRADE assessment for ASIA pinprick score at 6 months and 12 months

1. **6mo pinprick score**

| Certainty of the evidence, and classification of intervention | Intervention | Intervention v Baseline (mean difference (95% credible interval)) | Surface under the cumulative ranking curve |
| --- | --- | --- | --- |
| High certainty (moderate to high certainty evidence) | | | |
| Category 2: among the most effective | MSC (M) | **14.54(9.76, 19.46)** | 88.99% |
| Category 1: inferior to the most effective, or superior to the least effective | - | - | - |
| Category 0: among the least effective | OEC (M) | 3.13(-4.16, 10.54) | 38.89% |
| Low certainty (low to very low certainty evidence) | | | |
| Category 2: might be among the most effective | - | - | - |
| Category 1: might be inferior to the most effective or superior than the least effective | Macrophage (VL) | **11.68(0.68, 22.92)** | 76.41% |
| Category 0: might be among the least effective | NSC (VL) | 3.07(-4.58, 10.72) | 40.15% |
|  | Schwann (VL) | 1.66(-23.73, 25.95) | 36.35% |

1. **12mo pinprick score**

| Certainty of the evidence, and classification of intervention | Intervention | Intervention v Baseline (mean difference (95% credible interval)) | Surface under the cumulative ranking curve |
| --- | --- | --- | --- |
| High certainty (moderate to high certainty evidence) | | | |
| Category 2: among the most effective | MSC (M) | **12.48(7.09, 18.12)** | 78.51% |
| Category 1: inferior to the most effective, or superior to the least effective | - | - | - |
| Category 0: among the least effective | OEC (M) | 3.01(-0.45, 7.03) | 31.38% |
| Low certainty (low to very low certainty evidence) | | | |
| Category 2: might be among the most effective | - | - | - |
| Category 1: might be inferior to the most effective or superior than the least effective | Macrophage (VL) | **16.1(2.33, 30.17)** | 83.97% |
| Category 0: might be among the least effective | NSC (L) | 7.2(-1.22, 15.89) | 51.62% |

Table S11. GRADE assessment for FIM score at 6 months and 12 months

1. **6mo FIM score**

| Certainty of the evidence, and classification of intervention | Intervention | Intervention v Baseline (mean difference (95% credible interval)) | Surface under the cumulative ranking curve |
| --- | --- | --- | --- |
| High certainty (moderate to high certainty evidence) | | | |
| Category 2: among the most effective | - | - | - |
| Category 1: inferior to the most effective, or superior to the least effective | OEC(M) | **9.35(1.71, 17)** | 83.75% |
| Category 0: among the least effective | MSC (M) | 2.81(-2.89, 8.66) | 45.49% |
| Low certainty (low to very low certainty evidence) | | | |
| Category 2: might be among the most effective | - | - | - |
| Category 1: might be inferior to the most effective or superior than the least effective | - | - | - |
| Category 0: might be among the least effective | - | - | - |

1. **12mo FIM score**

| Certainty of the evidence, and classification of intervention | Intervention | Intervention v Baseline (mean difference (95% credible interval)) | Surface under the cumulative ranking curve |
| --- | --- | --- | --- |
| High certainty (moderate to high certainty evidence) | | | |
| Category 2: among the most effective | - | - | - |
| Category 1: inferior to the most effective, or superior to the least effective | - | - | - |
| Category 0: among the least effective | - | - | - |
| Low certainty (low to very low certainty evidence) | | | |
| Category 2: might be among the most effective | Macrophage (VL) | **42.83(36.33, 49.18)** | 86.29% |
|  | OEC (VL) | **21.02(9.75, 32.2)** | 53.90% |
| Category 1: might be inferior to the most effective or superior than the least effective | Schwann (VL) | **34.52(14.89, 54.23)** | 72.86% |
| Category 0: might be among the least effective | MSC (L) | 1.26(-4.77, 7.48) | 22.18% |

Table S12. GRADE assessment for IANR-SCIFRS at 6 months and 12 months

1. **6mo IANR-SCIFRS**

| Certainty of the evidence, and classification of intervention | Intervention | Intervention v Baseline (mean difference (95% credible interval)) | Surface under the cumulative ranking curve |
| --- | --- | --- | --- |
| High certainty (moderate to high certainty evidence) | | | |
| Category 2: among the most effective | - | - | - |
| Category 1: inferior to the most effective, or superior to the least effective | - | - | - |
| Category 0: among the least effective | MSC (M) | **3.96(0.62, 6.97)** | 66.99% |
| Low certainty (low to very low certainty evidence) | | | |
| Category 2: might be among the most effective | - | - | - |
| Category 1: might be inferior to the most effective or superior than the least effective | - | - | - |
| Category 0: might be among the least effective | OEC (VL) | 6.28(-5.26, 17.67) | 74.65% |

1. **12mo IANR-SCIFRS**

| Certainty of the evidence, and classification of intervention | Intervention | Intervention v Baseline (mean difference (95% credible interval)) | Surface under the cumulative ranking curve |
| --- | --- | --- | --- |
| High certainty (moderate to high certainty evidence) | | | |
| Category 2: among the most effective | - | - | - |
| Category 1: inferior to the most effective, or superior to the least effective | MSC (M) | **5.54(2.45, 8.42)** | 96.84% |
| Category 0: among the least effective | OEC (M) | 2.16(-0.24, 5.22) | 51.15% |
| Low certainty (low to very low certainty evidence) | | | |
| Category 2: might be among the most effective | - | - | - |
| Category 1: might be inferior to the most effective or superior than the least effective | - | - | - |
| Category 0: might be among the least effective | - | - | - |

Table S13. Severe adverse event.

| Severe adverse event | Total |
| --- | --- |
| CSF | 44 |
| Others/unknown | 14 |
| NNSI | 12 |
| AD | 5 |
| Constipation | 4 |
| Pseudomeningocele | 3 |
| DVT or PE | 3 |
| Meningitis | 3 |
| Convulsions | 2 |
| DSI | 1 |
| Atelectasis | 1 |
| RES | 1 |
| Osteomyelitis | 1 |
| Total events | 94 |

CSF: Cerebrospinal fluid leakage; NNSI: Non-central nervous system infection; AD: Autonomic dysreflexia; DSI: Delayed spinal instability; RES: Reversible encephalopathy syndrome.

Table S14. Pairwise result of different outcomes (comparing with baseline).

| Outcomes | Treatments | mean difference | 95%CI | | I2 |
| --- | --- | --- | --- | --- | --- |
| 6mo AIS | Combined | 0 | -0.98 | 0.98 | - |
|  | Macrophage | **0.81** | **0.05** | **1.56** | 0 |
|  | MSC | **0.96** | **0.28** | **1.63** | 0 |
|  | NSC | 0.17 | -0.19 | 0.53 | 0 |
|  | OEC | 0.22 | -1.42 | 1.87 | 40% |
|  | Schwann | 0.17 | -0.59 | 0.94 | 0 |
| 12mo AIS | Combined | 0 | -0.98 | 0.98 | - |
|  | Macrophage | 0.81 | -0.22 | 1.85 | 0 |
|  | MSC | **0.54** | **0.08** | **0.99** | 0 |
|  | NSC | 0.31 | -0.15 | 0.77 | 0 |
|  | OEC | 0.61 | -3.43 | 4.72 | 19% |
|  | Schwann | 0.39 | -1.98 | 2.75 | 0 |
| 6mo  motor | Combined | 0 | 0 | 0 | 0 |
|  | Macrophage | 0.3 | -0.69 | 1.2 | - |
|  | MSC | 0.37 | -0.25 | 0.98 | 42% |
|  | NSC | 0.5 | -0.15 | 1.14 | - |
|  | OEC | 0.24 | -0.10 | 0.58 | 0 |
|  | Schwann | 0.05 | -0.93 | 1.03 |  |
| 12mo  motor | Combined | 0 | 0 | 0 | 0 |
|  | Macrophage | 0.48 | -0.52 | 1.47 | - |
|  | MSC | 0.25 | -0.20 | 0.69 | 0 |
|  | NSC | **0.62** | **0.1** | **1.13** | 0 |
|  | OEC | **0.21** | **0.03** | **0.38** | 0 |
| 6mo  light touch | Macrophage | **1.07** | **0** | **2.14** | - |
|  | MSC | **0.76** | **0.34** | **1.18** | 12% |
|  | NSC | 0.28 | -0.36 | 0.92 | - |
|  | OEC | 0.13 | -0.50 | 0.77 | 0 |
|  | Schwann | -0.02 | -1 | 0.96 |  |
| 12mo  light touch | Macrophage | **1.12** | **0.04** | **2.2** | - |
|  | MSC | 0.91 | -0.21 | 2.04 | 71% |
|  | NSC | 0.32 | -0.91 | 1.55 | 0 |
|  | OEC | **0.27** | **0.1** | **0.45** | 0 |
| 6mo  pinprick | Macrophage | **1.08** | **0.01** | **2.15** | - |
|  | MSC | **0.92** | **0.26** | **1.58** | 54% |
|  | NSC | 0.34 | -0.3 | 0.98 | - |
|  | OEC | 0.24 | -0.40 | 0.89 | 0 |
|  | Schwann | 0.05 | -0.93 | 1.03 |  |
| 12mo pinprick | Macrophage | **1.55** | **0.07** | **2.24** | - |
|  | MSC | 0.88 | -0.17 | 1.93 | 70% |
|  | NSC | **0.57** | **0.53** | **0.6** | 0 |
|  | OEC | 0.22 | -0.01 | 0.45 | 0 |
| 6mo FIM score | MSC | 0.5 | -1.21 | 2.22 | 40% |
|  | OEC | 1.21 | -12.01 | 14.44 | 74% |
| 12mo FIM score | Macrophage | **12.12** | **9.63** | **14.6** | - |
|  | MSC | 0.45 | -4.86 | 4.33 | 23% |
|  | OEC | **2.06** | **0.78** | **3.34** | - |
|  | Schwann | **1.89** | **0.43** | **3.35** | - |
| 6mo IANR-SCIFRS | MSC | 0.67 | -0.47 | 1.81 | 61% |
|  | OEC | 0.6 | -0.57 | 1.77 | - |
| 12mo IANR-SCIFRS | OEC | **0.4** | **0.24** | **0.56** | 0 |
|  | MSC | 0.95 | -0.46 | 2.37 | 72% |

Table S15. Network meta-regression (assessment of the transitivity).

| Outcomes | Covariate | Treatments | Beta | 95%CI | | Centering Value | I2 |
| --- | --- | --- | --- | --- | --- | --- | --- |
| 6mo AIS | baseline | d.Baseline.Combined | -0.76804 | -6.28267 | 2.3541 | 1.31 | 0 |
|  |  | d.Baseline.Macrophage | 0.04204 | -4.794102 | 5.249 |  |  |
|  |  | d.Baseline.MSC | 0.09646 | -1.33267 | 1.6272 |  |  |
|  |  | d.Baseline.NSC | -0.13345 | -0.869095 | 0.598 |  |  |
|  |  | d.Baseline.OEC | 0.71577 | -0.236351 | 1.7891 |  |  |
|  |  | d.Baseline.Schwann | -0.11393 | -0.870874 | 0.6303 |  |  |
|  | year | d.Baseline.Combined | -0.18046 | -3.774383 | 2.2427 | 2013.75 | 0 |
|  |  | d.Baseline.Macrophage | -0.25015 | -1.151972 | 0.6195 |  |  |
|  |  | d.Baseline.MSC | -0.6048 | -2.184205 | 0.7355 |  |  |
|  |  | d.Baseline.NSC | -0.15001 | -1.131709 | 0.7987 |  |  |
|  |  | d.Baseline.OEC | 1.0666 | -0.490525 | 2.5503 |  |  |
|  |  | d.Baseline.Schwann | -0.04069 | -1.030214 | 0.938 |  |  |
|  | cell | d.Baseline.Combined | -0.066602 | -2.788163 | 2.7881 | 3.6875 | 0 |
|  |  | d.Baseline.Macrophage | -0.448344 | -10.671418 | 4.9556 |  |  |
|  |  | d.Baseline.MSC | 0.266042 | -4.917095 | 8.8556 |  |  |
|  |  | d.Baseline.NSC | 1.463799 | -6.452206 | 20.3131 |  |  |
|  |  | d.Baseline.OEC | -0.614824 | -2.344619 | 2.7258 |  |  |
|  |  | d.Baseline.Schwann | 0.075354 | -8.932526 | 10.5738 |  |  |
| 12mo AIS | baseline | d.Baseline.Combined | -1.54037 | -6.210157 | 1.3529 | 1.302857 | 0 |
|  |  | d.Baseline.Macrophage | -0.32657 | -10.142578 | 5.9352 |  |  |
|  |  | d.Baseline.MSC | 0.31007 | -1.680365 | 2.3842 |  |  |
|  |  | d.Baseline.NSC | -0.43635 | -1.263143 | 0.3918 |  |  |
|  |  | d.Baseline.OEC | 0.71449 | -0.410348 | 1.9283 |  |  |
|  |  | d.Baseline.Schwann | 0.03828 | -1.148711 | 1.2215 |  |  |
|  | year | d.Baseline.Combined | -2.21597 | -26.907714 | 5.6041 | 2013.214 | 0.40% |
|  |  | d.Baseline.Macrophage | -0.37978 | -1.486259 | 0.6881 |  |  |
|  |  | d.Baseline.MSC | 0.23663 | -0.753393 | 1.1865 |  |  |
|  |  | d.Baseline.NSC | -0.40601 | -1.658759 | 0.7779 |  |  |
|  |  | d.Baseline.OEC | 1.12734 | -1.113079 | 4.1517 |  |  |
|  |  | d.Baseline.Schwann | -0.04222 | -1.604763 | 1.507 |  |  |
|  | cell | d.Baseline.Combined | 9.06641 | -7.43615 | 45.3946 | 3.857143 | 2% |
|  |  | d.Baseline.Macrophage | 1.61018 | -6.05623 | 33.1761 |  |  |
|  |  | d.Baseline.MSC | -0.43278 | -7.86245 | 3.6437 |  |  |
|  |  | d.Baseline.NSC | 0.20492 | -7.23767 | 8.2494 |  |  |
|  |  | d.Baseline.OEC | -0.71701 | -17.898 | 7.2267 |  |  |
|  |  | d.Baseline.Schwann | 0.57897 | -8.85962 | 17.0024 |  |  |
| 6mo motor | baseline | d.Baseline.Combined | 8.5351 | -95.4026 | 133.985 | 42.258 | 0 |
|  |  | d.Baseline.Macrophage | 8.5651 | -123.5779 | 197.47 |  |  |
|  |  | d.Baseline.MSC | 2.1975 | -17.7243 | 26.33 |  |  |
|  |  | d.Baseline.NSC | -0.4419 | -59.4734 | 58.183 |  |  |
|  |  | d.Baseline.OEC | -9.6554 | -38.926 | 10.155 |  |  |
|  |  | d.Baseline.Schwann | -10.9251 | -178.3481 | 73.287 |  |  |
|  | year | d.Baseline.Combined | 0.15534 | -31.502 | 32.835 | 2013.4 | 0 |
|  |  | d.Baseline.Macrophage | -0.34824 | -66.079 | 58.743 |  |  |
|  |  | d.Baseline.MSC | -1.05276 | -16.304 | 13.922 |  |  |
|  |  | d.Baseline.NSC | -0.71205 | -108.325 | 94.416 |  |  |
|  |  | d.Baseline.OEC | -11.06021 | -23.384 | 5.351 |  |  |
|  |  | d.Baseline.Schwann | 1.00044 | -60.223 | 66.445 |  |  |
|  | cell | d.Baseline.Combined | 0.907 | -53.28196 | 56.828 | 2.8 | 0 |
|  |  | d.Baseline.Macrophage | 1.4466 | -59.08938 | 72.534 |  |  |
|  |  | d.Baseline.MSC | 0.5619 | -81.87872 | 92.611 |  |  |
|  |  | d.Baseline.NSC | -2.8866 | -111.91934 | 75.999 |  |  |
|  |  | d.Baseline.OEC | -8.0575 | -43.26973 | 35.574 |  |  |
|  |  | d.Baseline.Schwann | -3.0575 | -78.60002 | 45.015 |  |  |
| 12mo motor | baseline | d.Baseline.Combined | 0.4797 | -106.96 | 109.39 | 36.89611 | 0.30% |
|  |  | d.Baseline.Macrophage | -5.7482 | -190.25 | 116.36 |  |  |
|  |  | d.Baseline.MSC | 0.3322 | -46.93 | 48.78 |  |  |
|  |  | d.Baseline.NSC | -4.309 | -49.53 | 30.47 |  |  |
|  |  | d.Baseline.OEC | -4.7478 | -62.82 | 41.63 |  |  |
|  | year | d.Baseline.Combined | 0.025808 | -34.782 | 34.83 | 2012.944 | 0 |
|  |  | d.Baseline.Macrophage | 1.058195 | -65.304 | 87.29 |  |  |
|  |  | d.Baseline.MSC | 1.58169 | -12.129 | 15.28 |  |  |
|  |  | d.Baseline.NSC | -8.153036 | -64.38 | 26.8 |  |  |
|  |  | d.Baseline.OEC | 6.087671 | -30.729 | 53.04 |  |  |
|  | cell | d.Baseline.Combined | -0.2326 | -8.39E+01 | 102.939 | 2.5 | 0 |
|  |  | d.Baseline.Macrophage | 1.231 | -1.30E+02 | 181.416 |  |  |
|  |  | d.Baseline.MSC | -2.8727 | -7.97E+01 | 60.184 |  |  |
|  |  | d.Baseline.NSC | -1.0316 | -4.95E+01 | 38.572 |  |  |
|  |  | d.Baseline.OEC | 10.6141 | -6.36E+00 | 48.422 |  |  |

Table S16. Comparisons of the fit of consistency and inconsistency models.

| Model | Consistency | | Inconsistency | |
| --- | --- | --- | --- | --- |
| Item | DIC | I2 | DIC | I2 |
| 6mo AIS | 55.05255 | 0 | 54.97613 | 0 |
| 12mo AIS | 50.71406 | 2% | 50.65873 | 2% |
| 6mo motor | 46.19081 | 0 | 46.0402 | 0 |
| 12mo motor | 58.55089 | 0 | 58.84026 | 0 |
| 6mo light touch | 42.1889 | 0 | 41.96948 | 0 |
| 12mo light touch | 53.37098 | 2% | 53.31493 | 2% |
| 6mo pinprick | 40.30426 | 0 | 40.29412 | 0.00% |
| 12mo pinprick | 55.69874 | 9% | 55.72921 | 9% |
| 6mo FIM score | 19.31 | 9% | 19.32 | 9% |
| 12mo FIM score | 19.1 | 6% | 19.02 | 5% |
| 6mo IANR-SCIFRS | 15.94 | 0 | 15.85 | 0 |
| 12mo IANR-SCIFRS | 26.35 | 0 | 26.44 | 0 |

## Supplementary Figures

Figure S1. The funnel plot for small sample effect test for AIS grade

1. **6mo AIS**


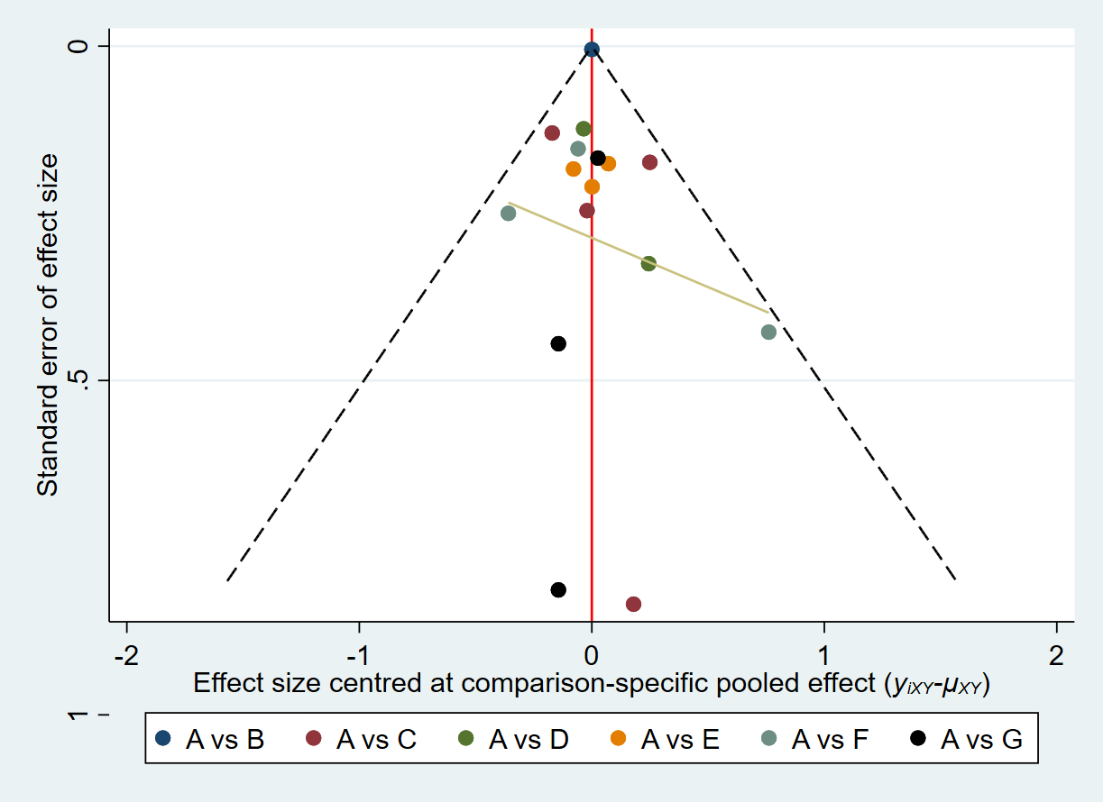


A: Baseline; B: Combined; C: MSC; D: Macrophage; E: NSC; F: OEC; G: Schwann

1. **12mo AIS**


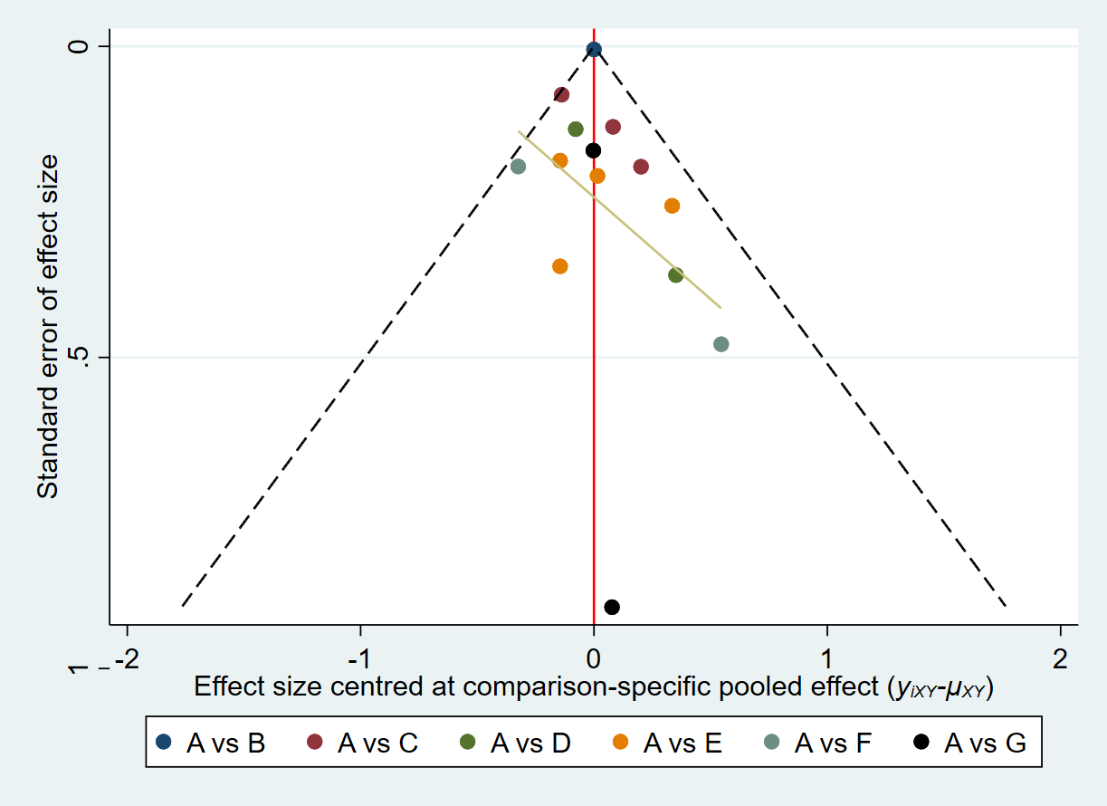


A: Baseline; B: Combined; C: MSC; D: Macrophage; E: NSC; F: OEC; G: Schwann

Figure S2. The funnel plot for small sample effect test for ASIA motor score.

1. **6mo motor score**


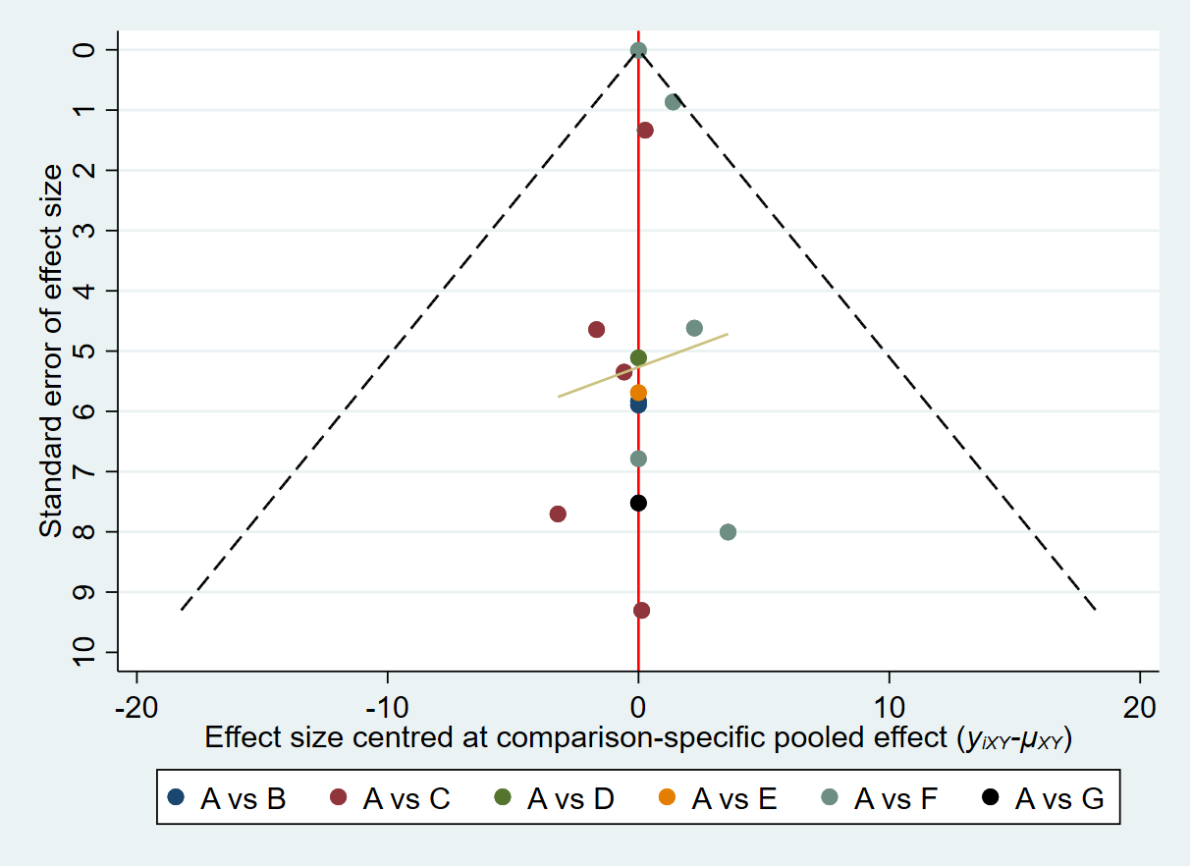


A: Baseline; B: Combined; C: MSC; D: Macrophage; E: NSC; F: OEC; G: Schwann

1. **12mo motor score**


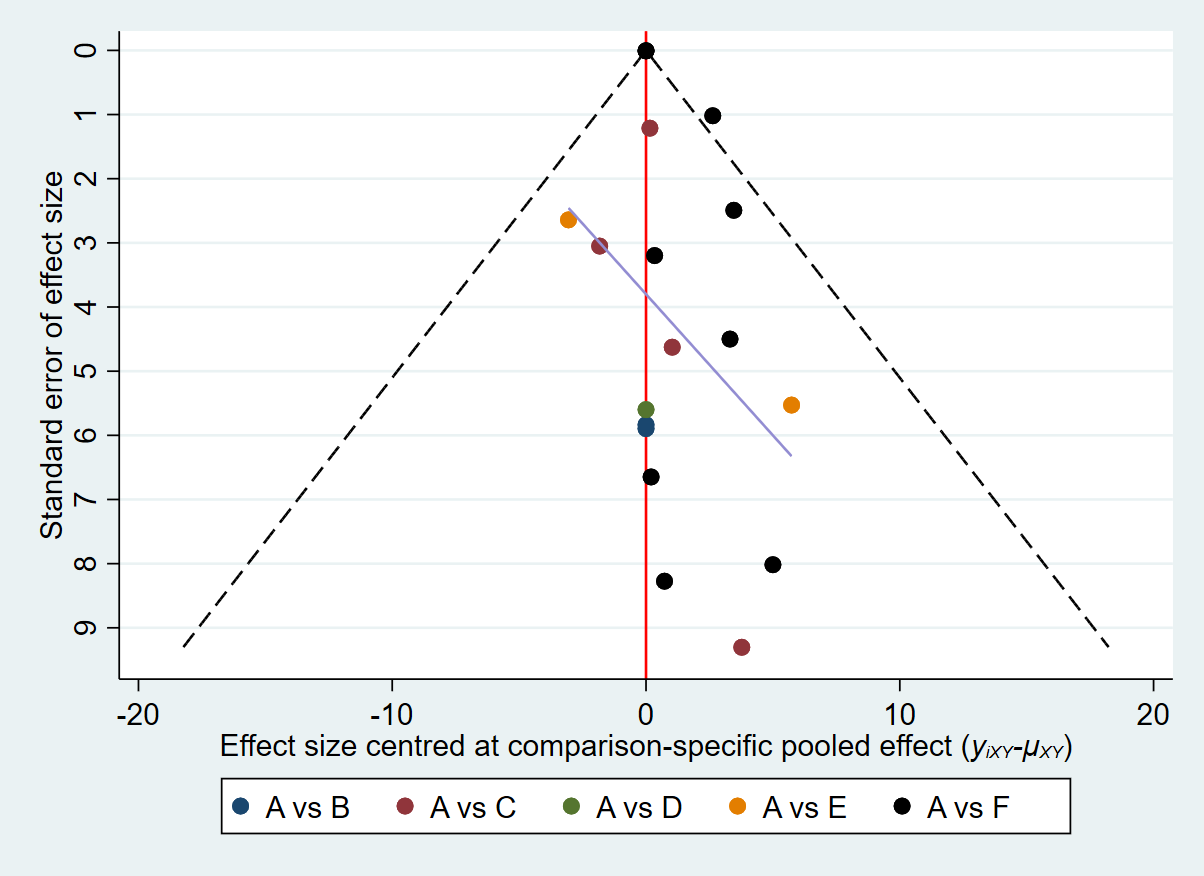


A: Baseline; B: Combined; C: MSC; D: Macrophage; E: NSC; F: OEC

Figure S3. The funnel plot for small sample effect test for ASIA light touch score

1. **6mo light touch score**


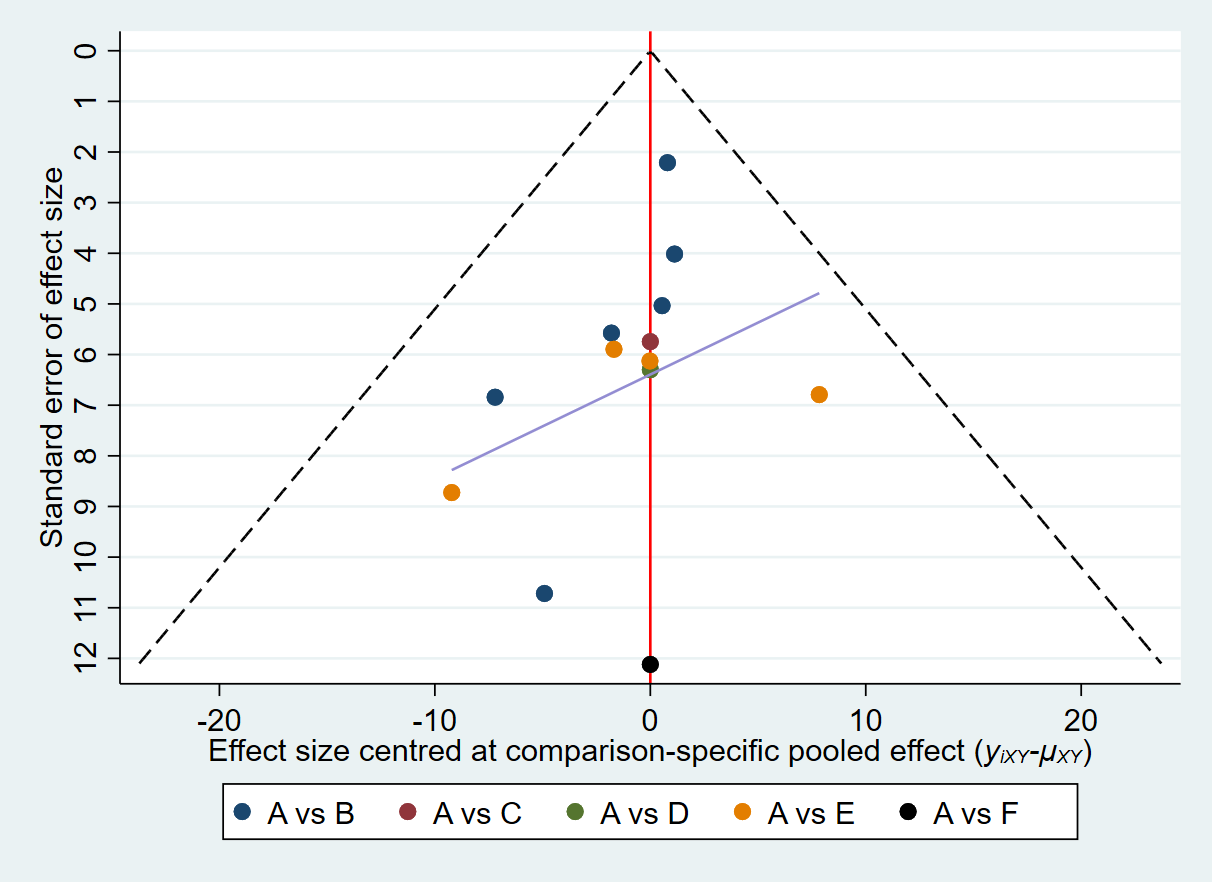


A: Baseline; B: MSC; C: Macrophage; D: NSC; E: OEC; F: Schwann

1. **12mo light touch score**


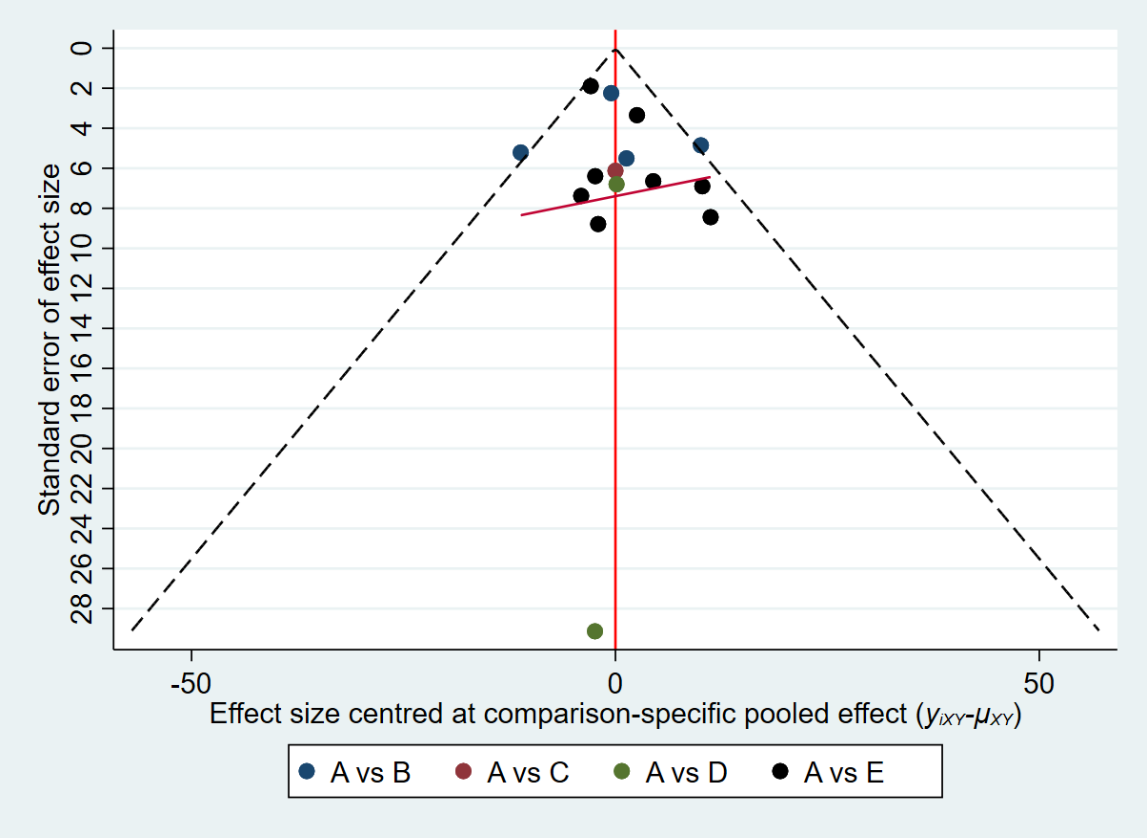


A: Baseline; B: MSC; C: Macrophage; D: NSC; E: OEC

Figure S4. The funnel plot for small sample effect test for ASIA pinprick score.

1. **6mo pinprick score**


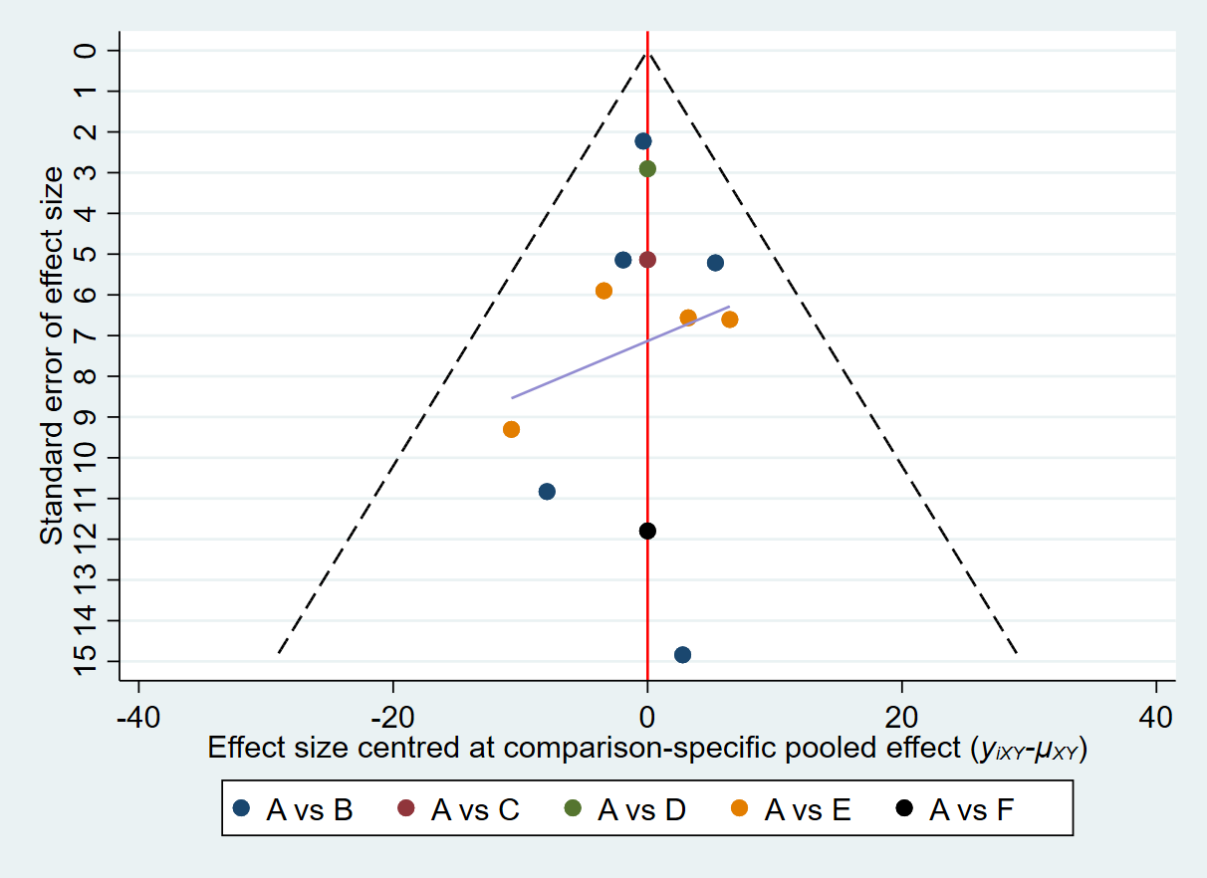


A: Baseline; B: MSC; C: Macrophage; D: NSC; E: OEC; F: Schwann

1. **12mo pinprick score**


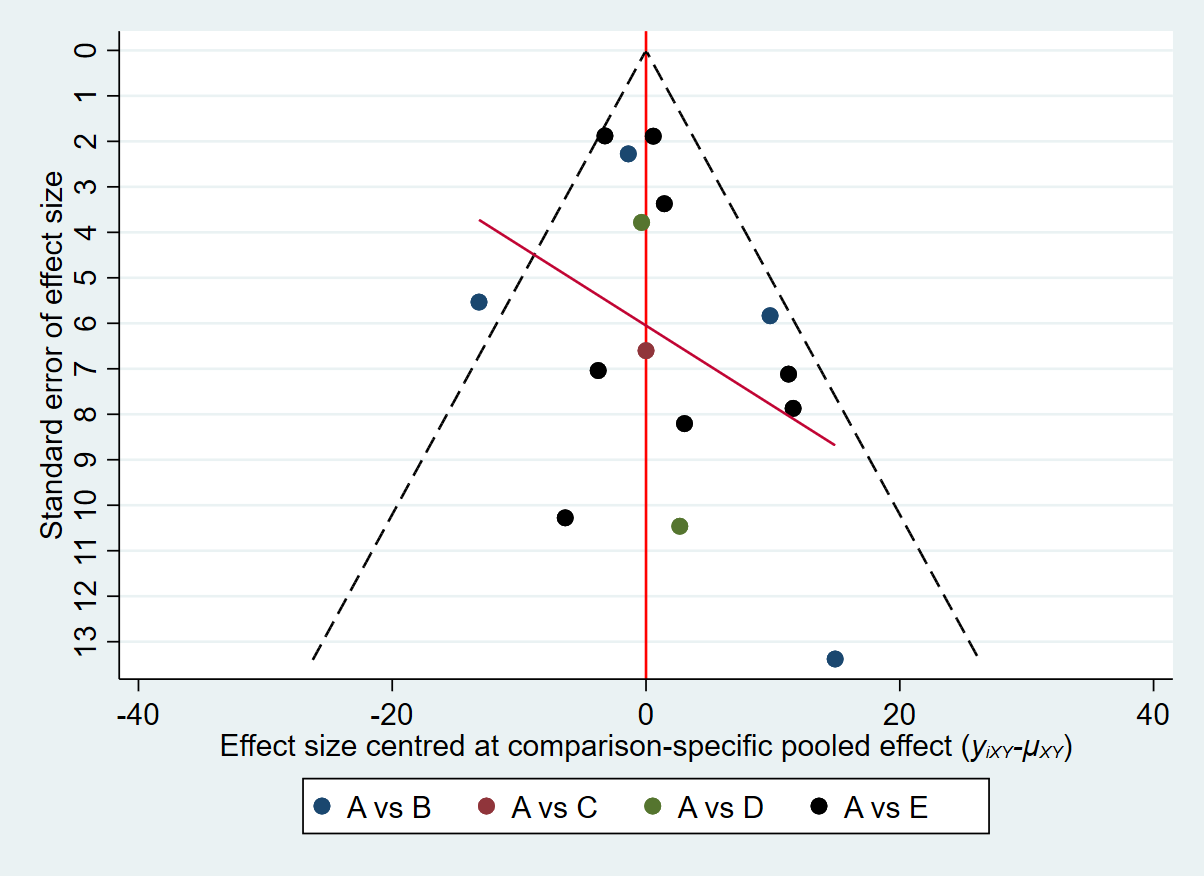


A: Baseline; B: MSC; C: Macrophage; D: NSC; E: OEC

Figure S5. Bean plot for mean age of included studies (assessment of the transitivity).


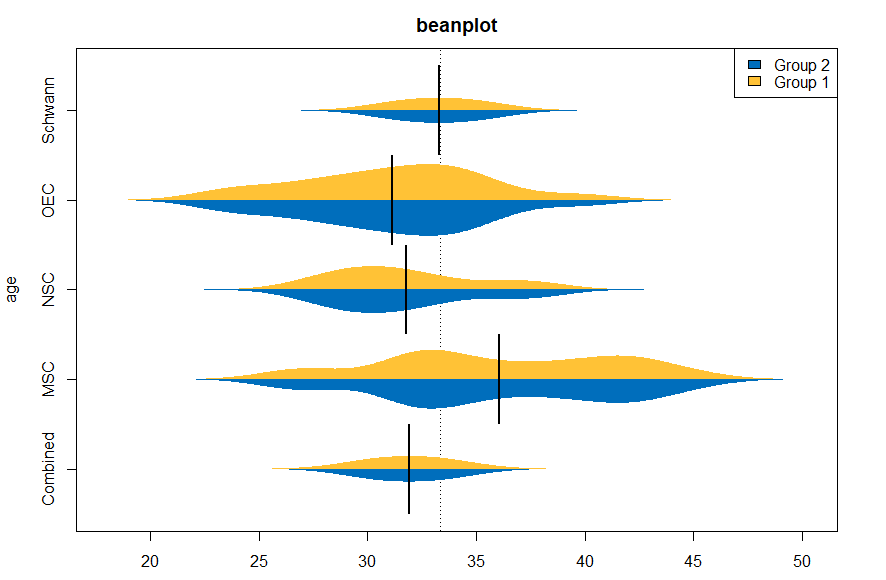


Figure S6. Assessment of the aggregation for AIS grade at 6 months.

**A. The trace plots and density plots.**


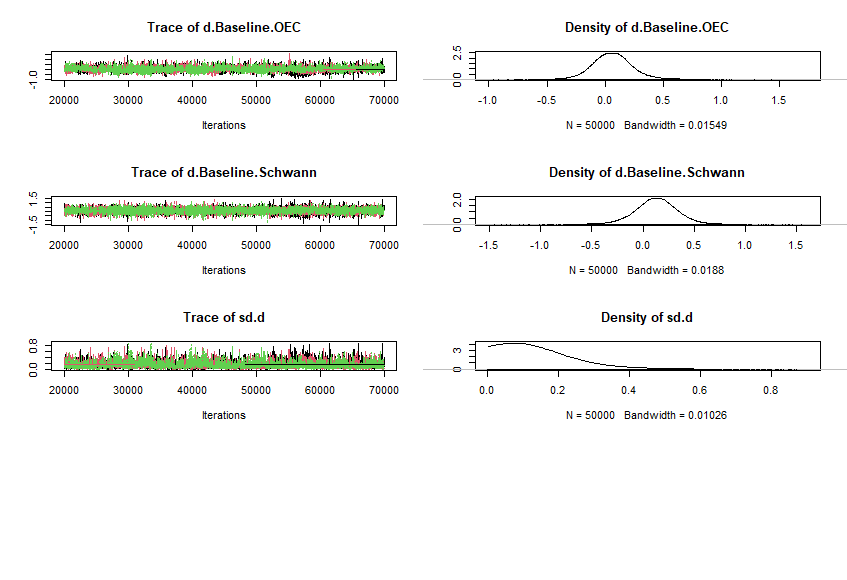


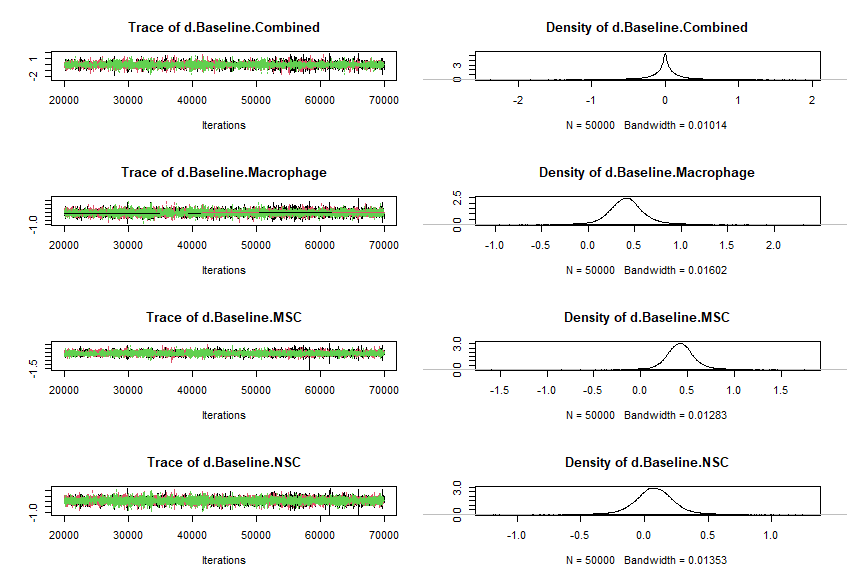


**B. The iterative plots.**


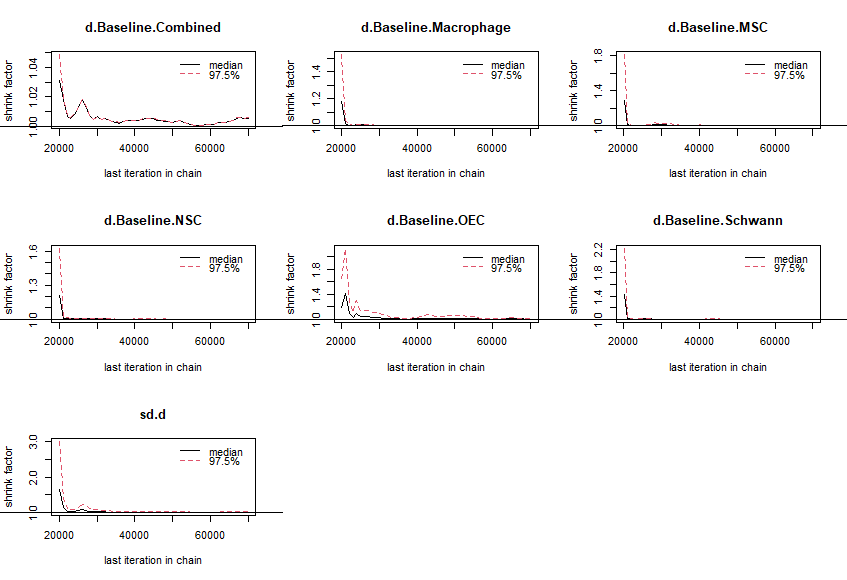


Figure S7. Assessment of the aggregation for AIS grade at 12 months.

**A. The trace plots and density plots.**


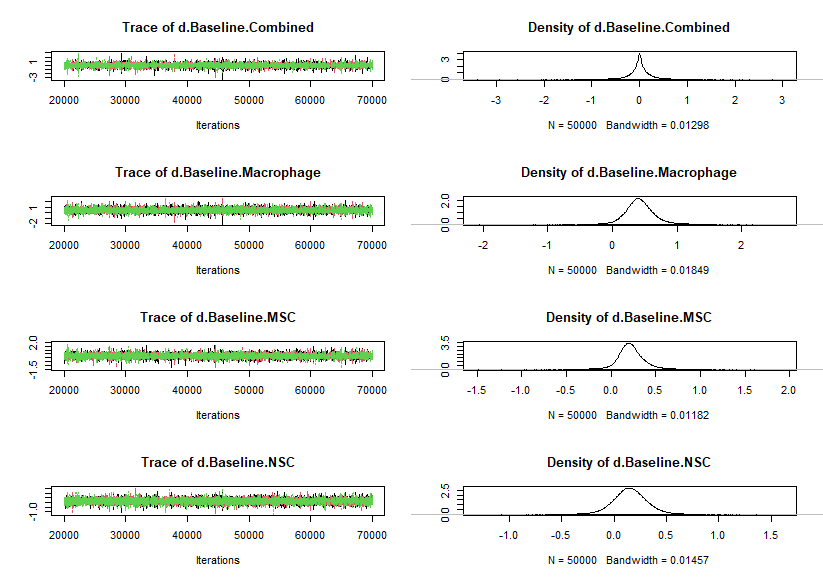

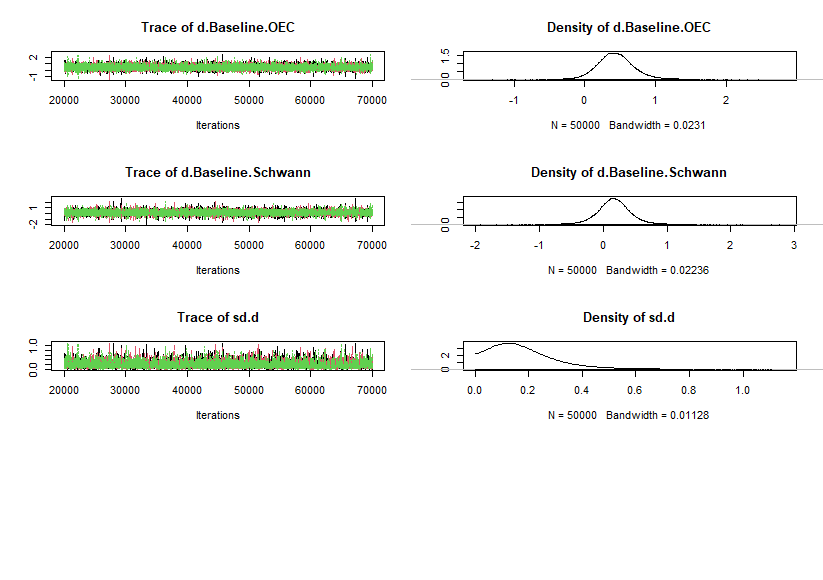


**B. The iterative plots.**


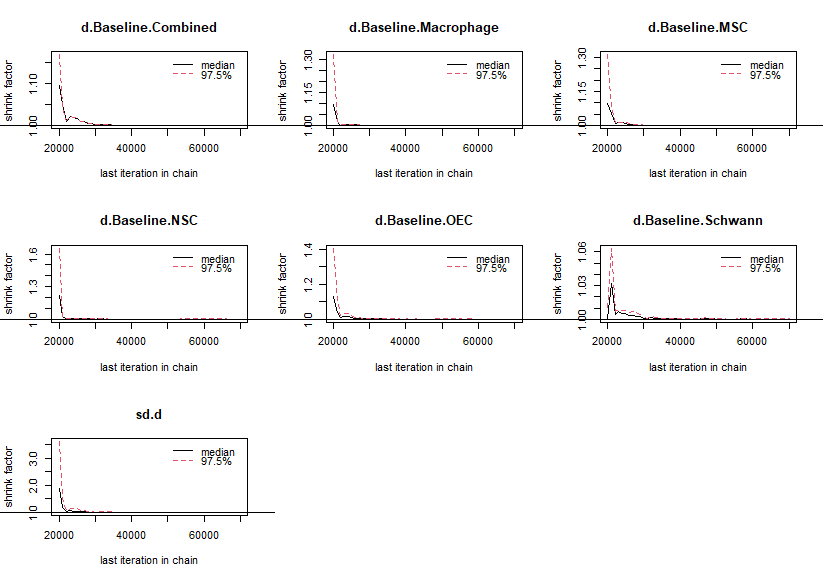


Figure S8. Assessment of the aggregation for ASIA motor score at 6 months.

**A. The trace plots and density plots.**


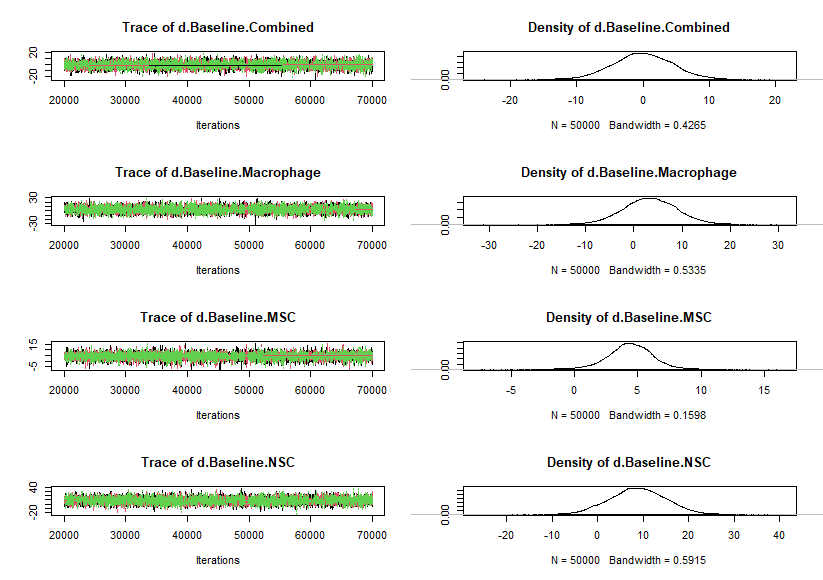

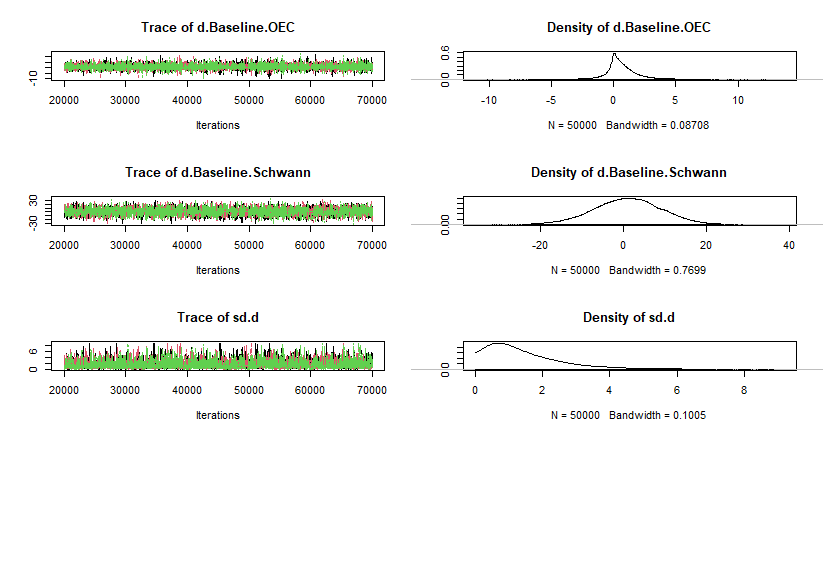


**B. The iterative plots.**


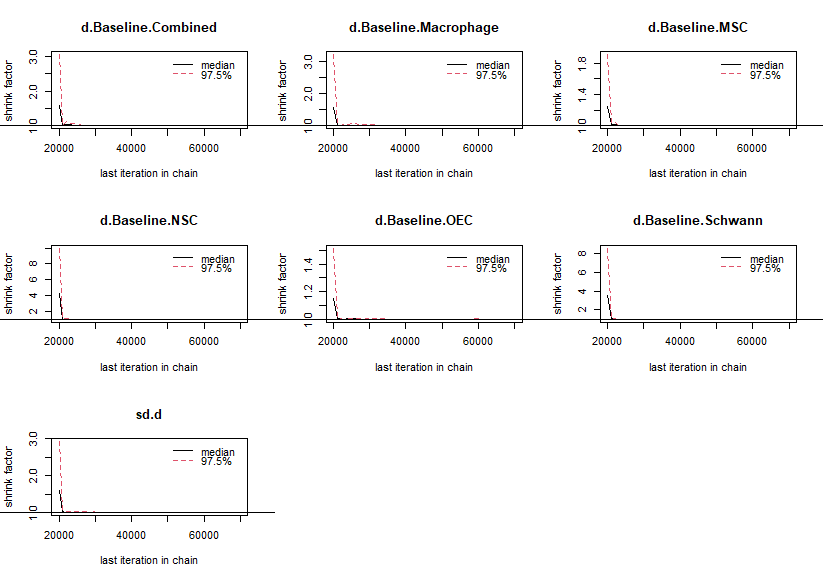


Figure S9. Assessment of the aggregation for ASIA motor score at 12 months.

**A. The trace plots and density plots.**


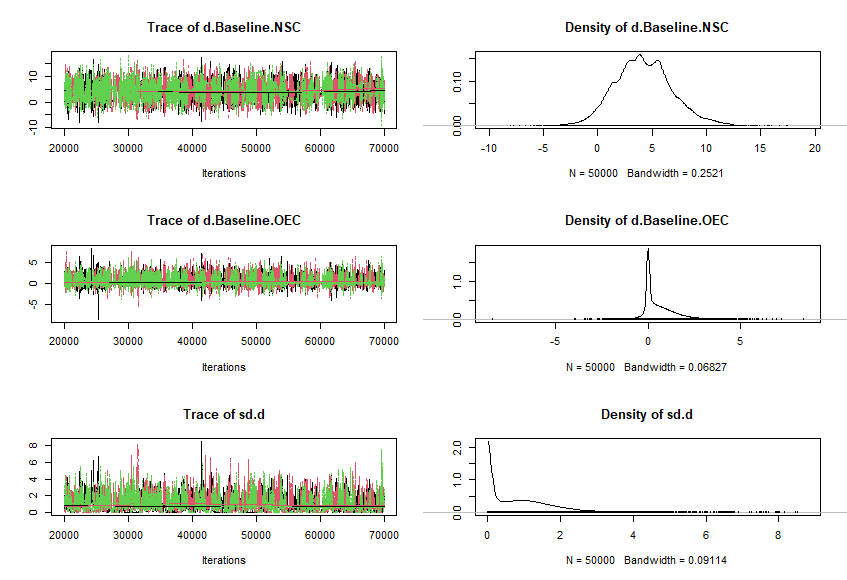

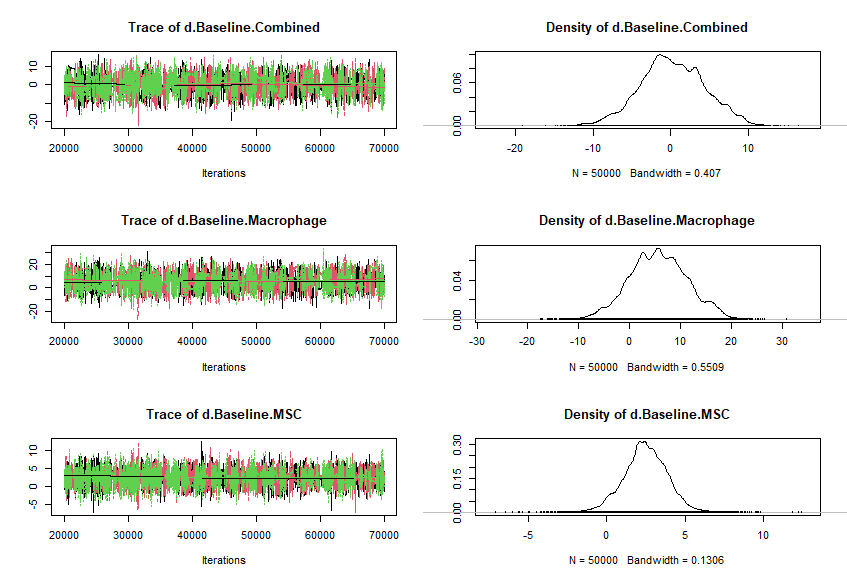


**B. The iterative plots.**


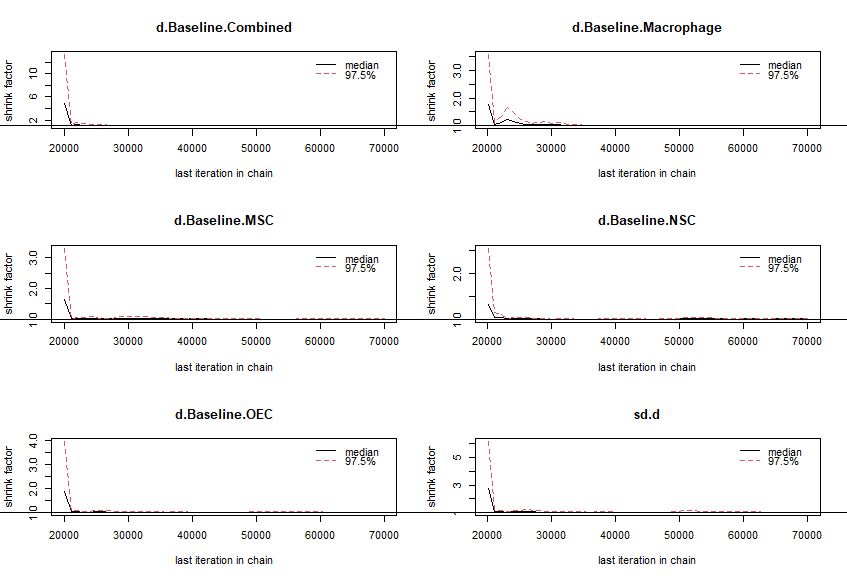


Figure S10. Assessment of the aggregation for ASIA light touch score at 6 months.

**A. The trace plots and density plots.**


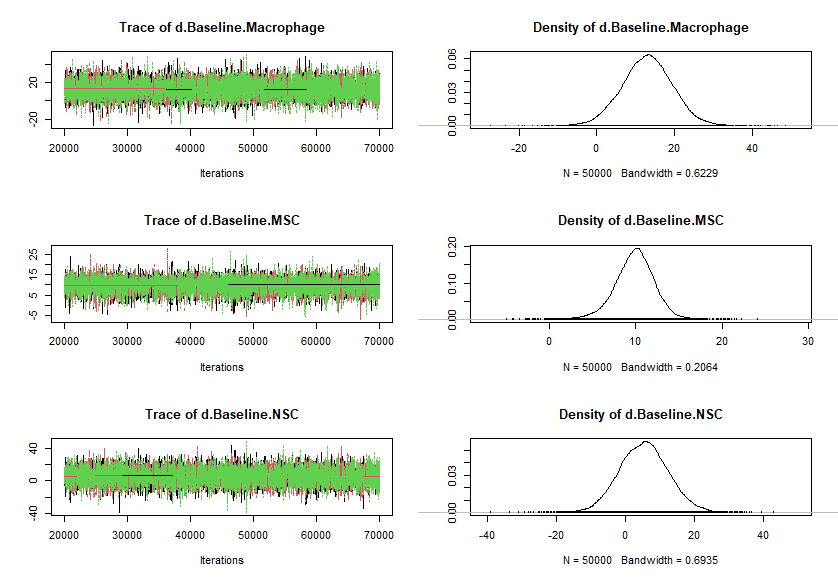

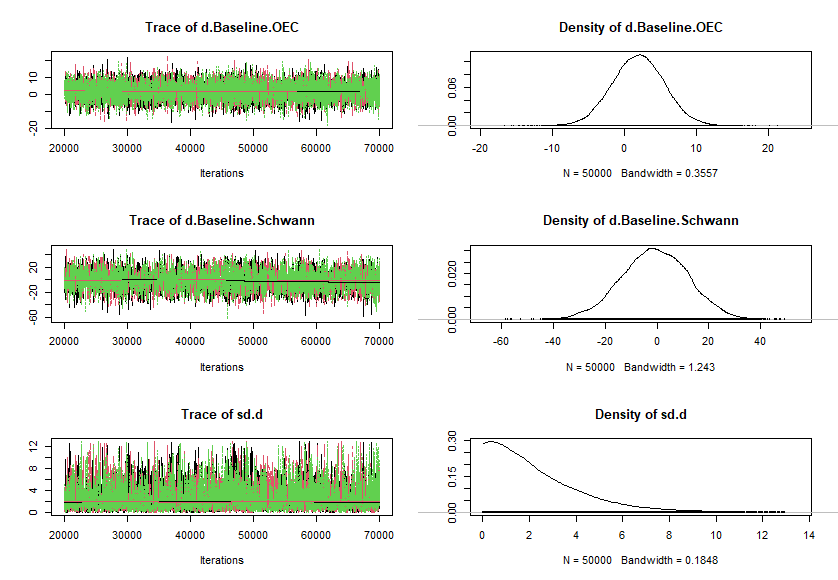


**B. The iterative plots.**


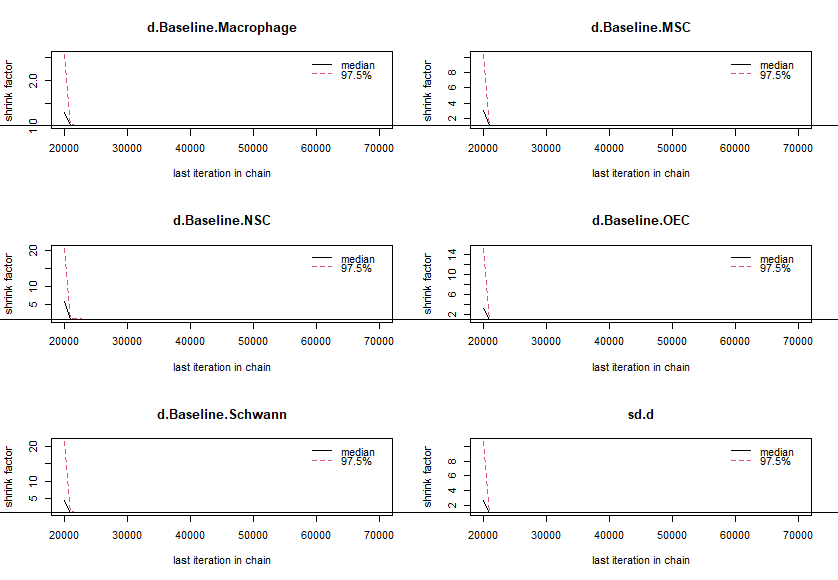


Figure S11. Assessment of the aggregation for ASIA light touch score at 12 months.

**A. The trace plots and density plots.**


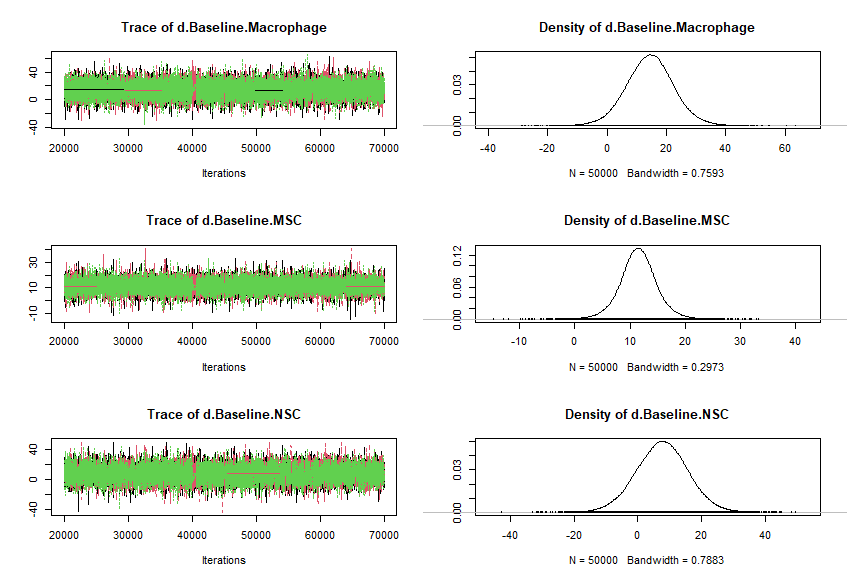

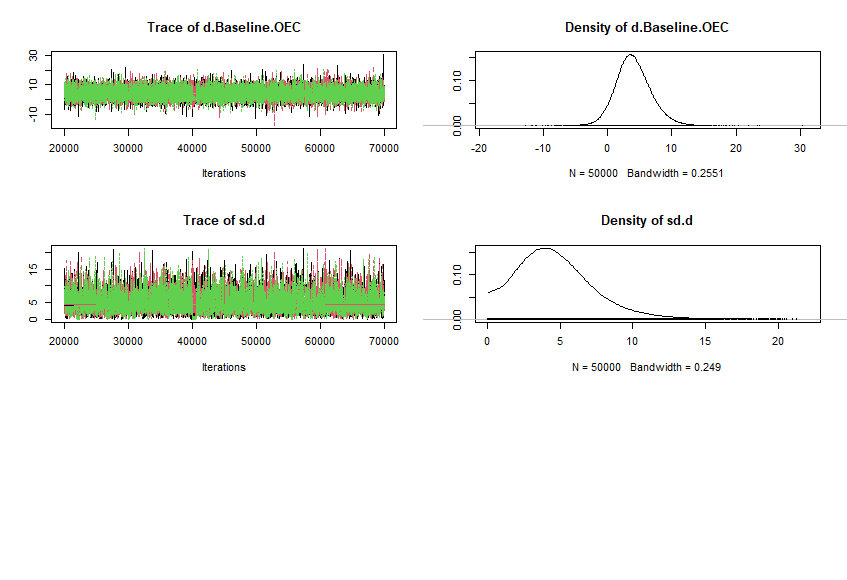


**B. The iterative plots.**


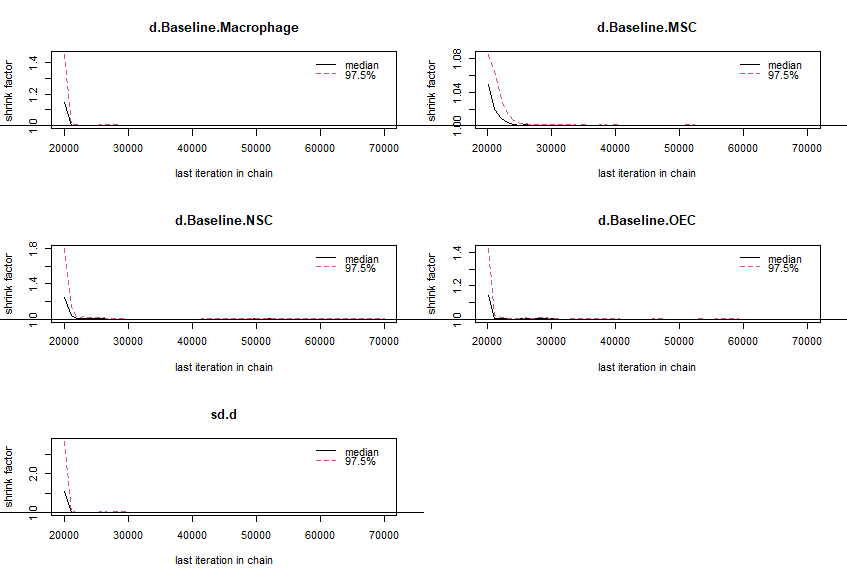


Figure S12. Assessment of the aggregation for ASIA pinprick score at 6 months.

**A. The trace plots and density plots.**


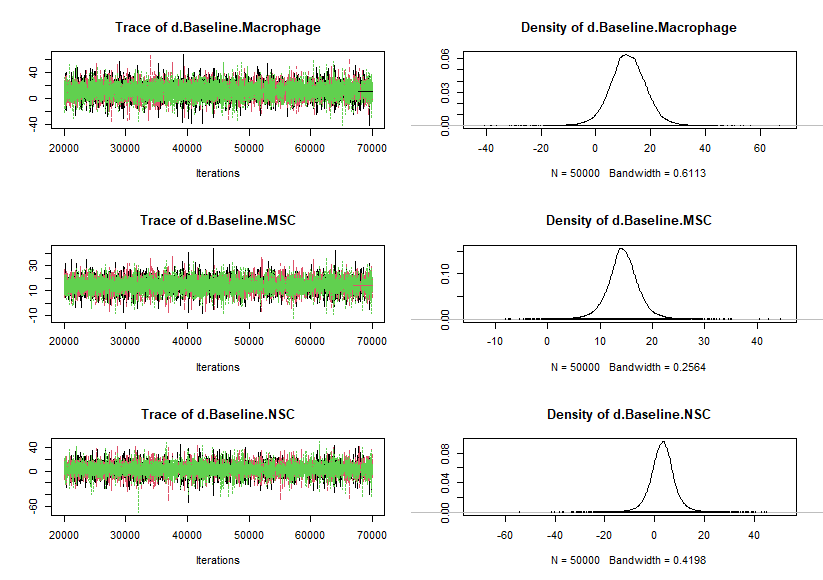

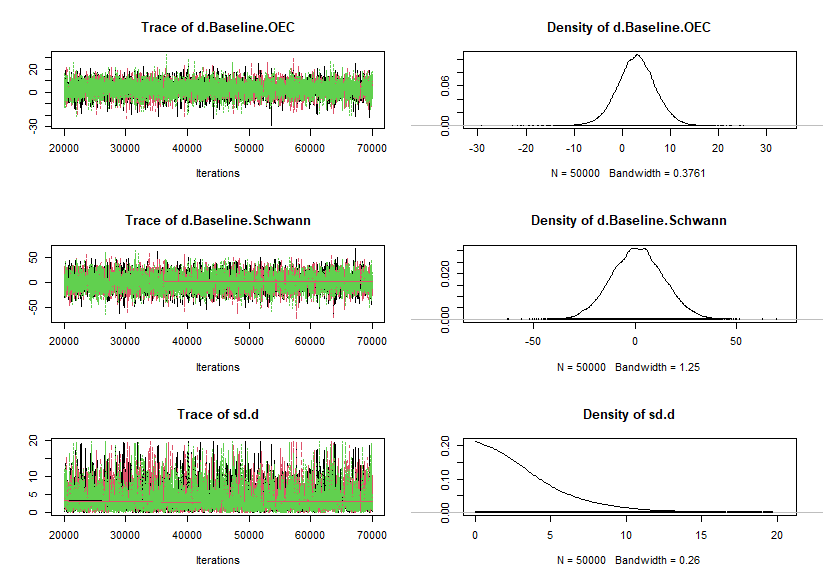


**B. The iterative plots.**


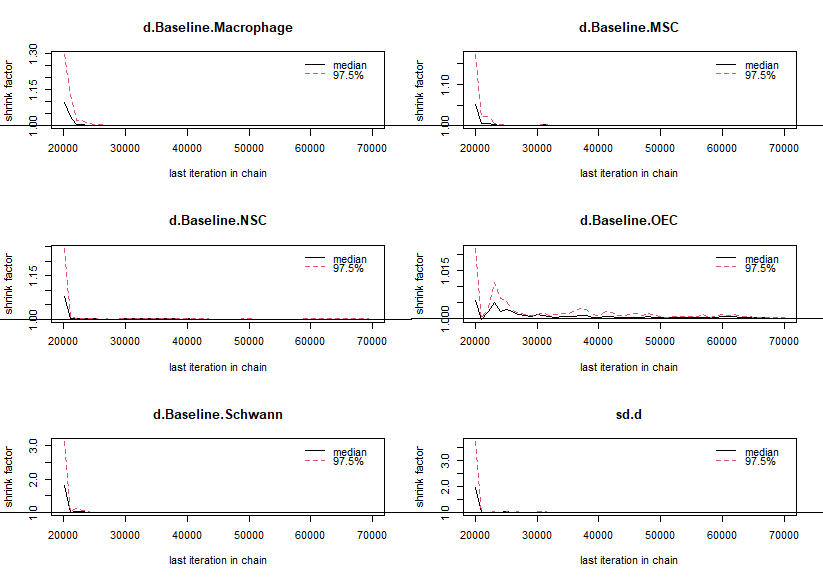


Figure S13. Assessment of the aggregation for ASIA pinprick score at 12 months.

**A. The trace plots and density plots.**


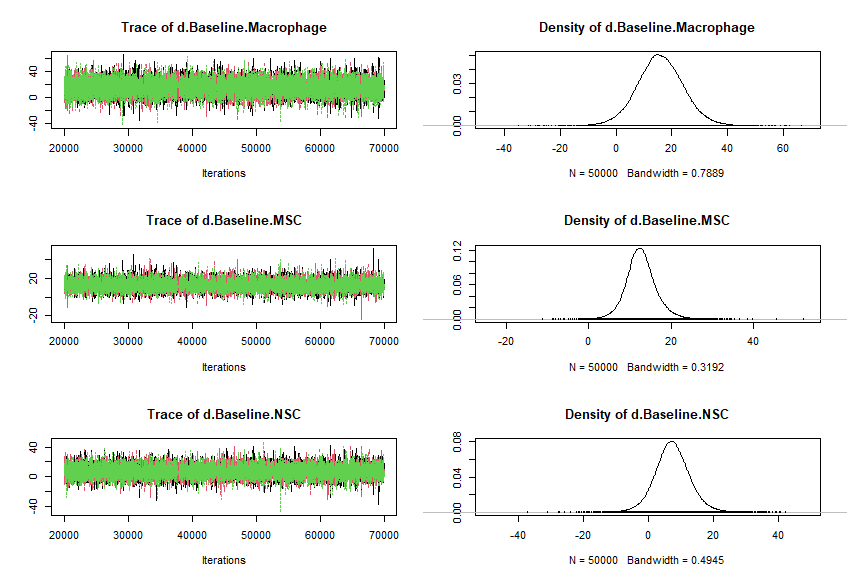

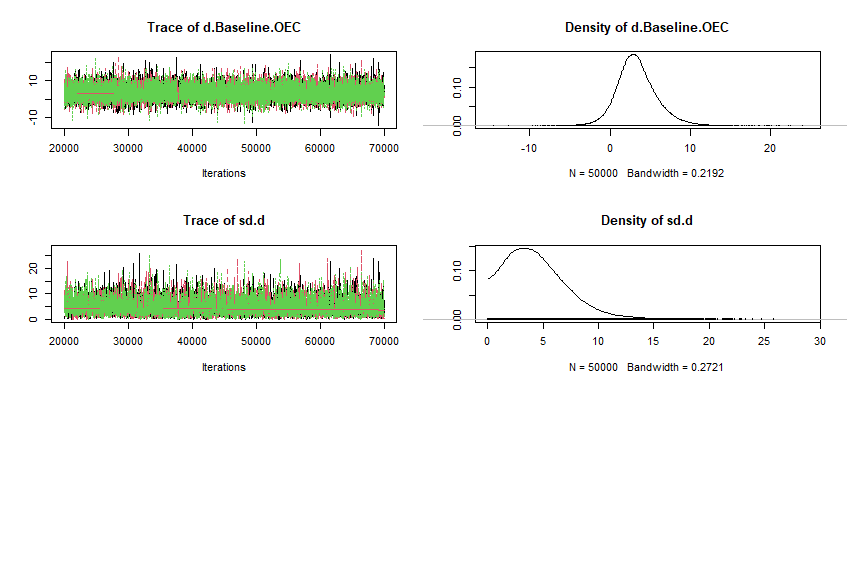


**B. The iterative plots.**


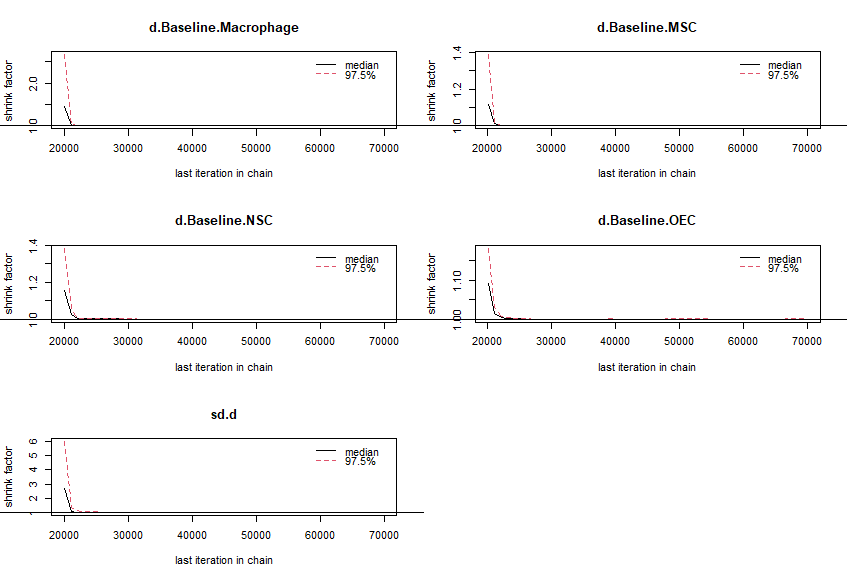


Figure S14. Assessment of the aggregation for FIM score at 6 months.

**A. The trace plots and density plots.**


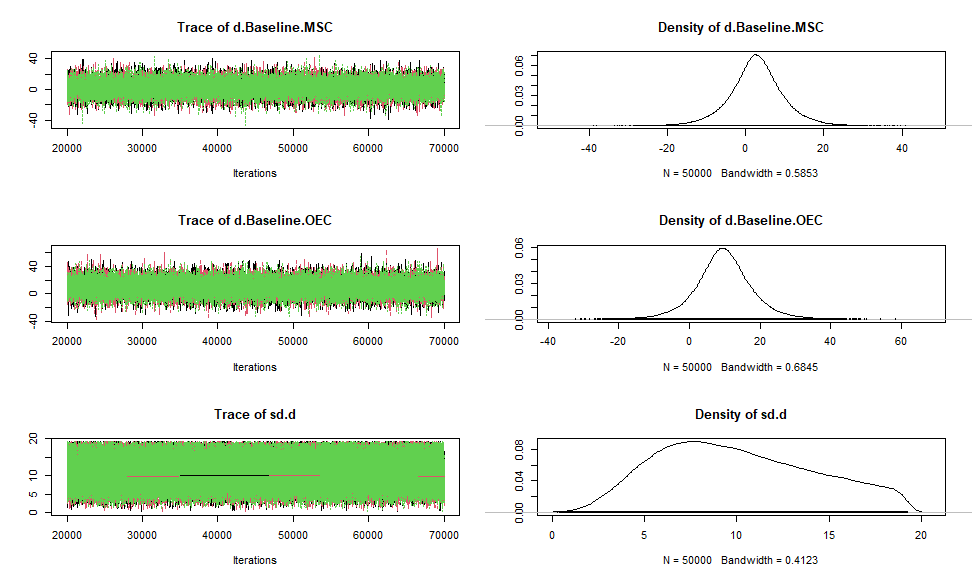

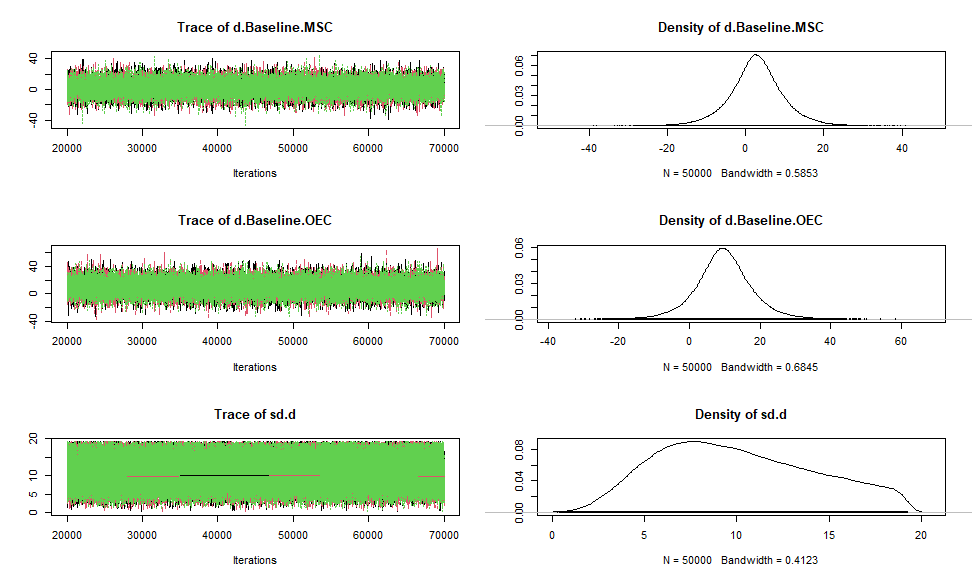


**B. The iterative plots**.


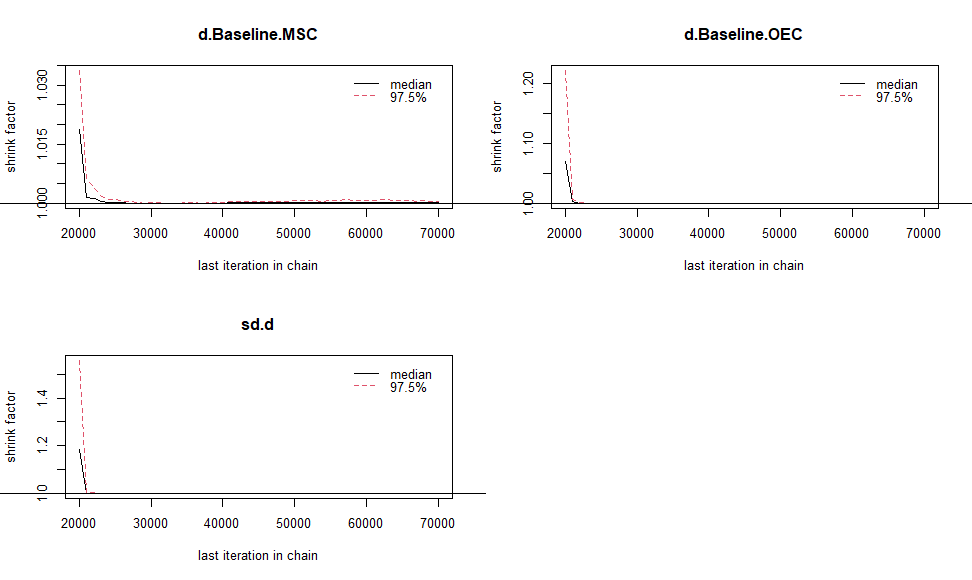


Figure S15. Assessment of the aggregation for FIM score at 12 months.

**A. The trace plots and density plots.**


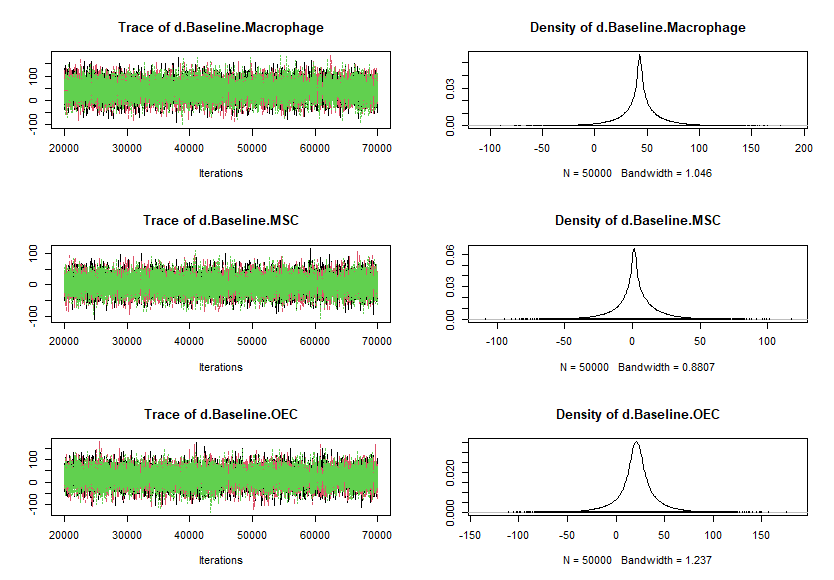

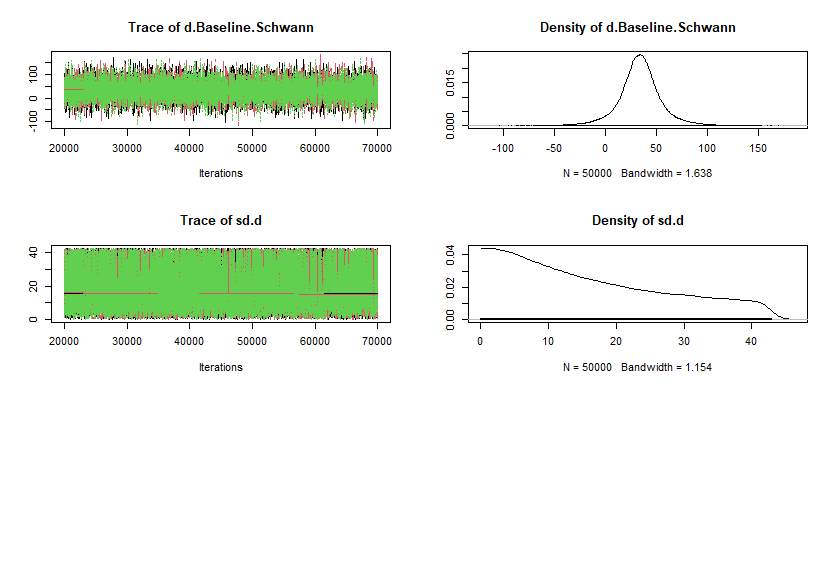


**B. The iterative plots.**


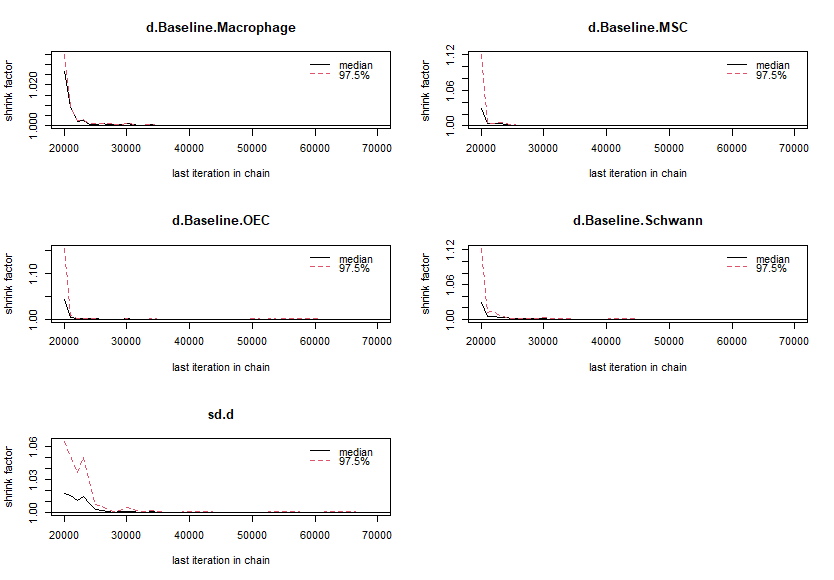


Figure S16. Assessment of the aggregation for IANR-SCIFRS at 6 months.

**A. The trace plots and density plots.**


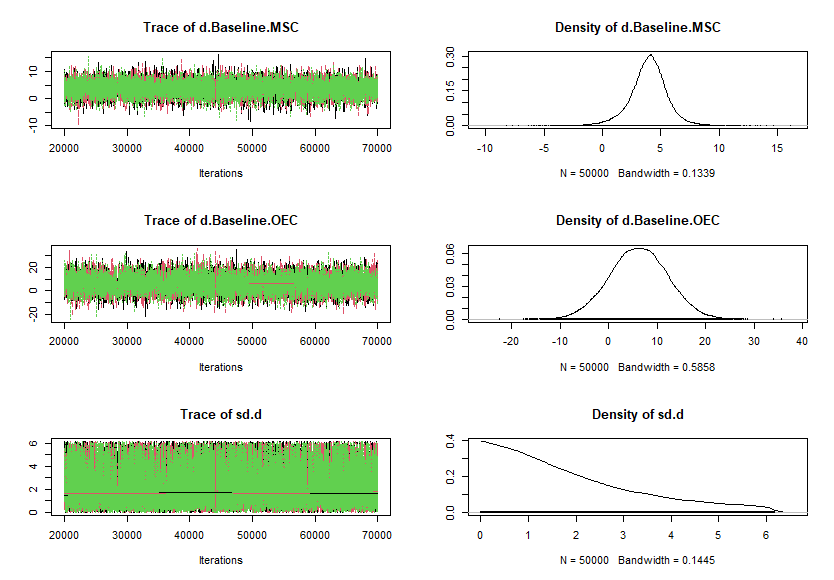


**B. The iterative plots.**


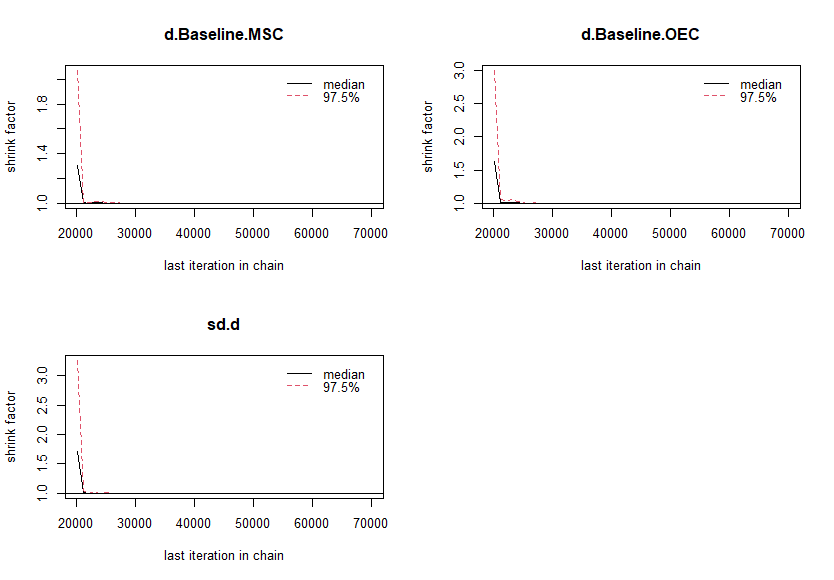


Figure S17. Assessment of the aggregation for IANR-SCIFRS at 12 months.

A. The trace plots and density plots.


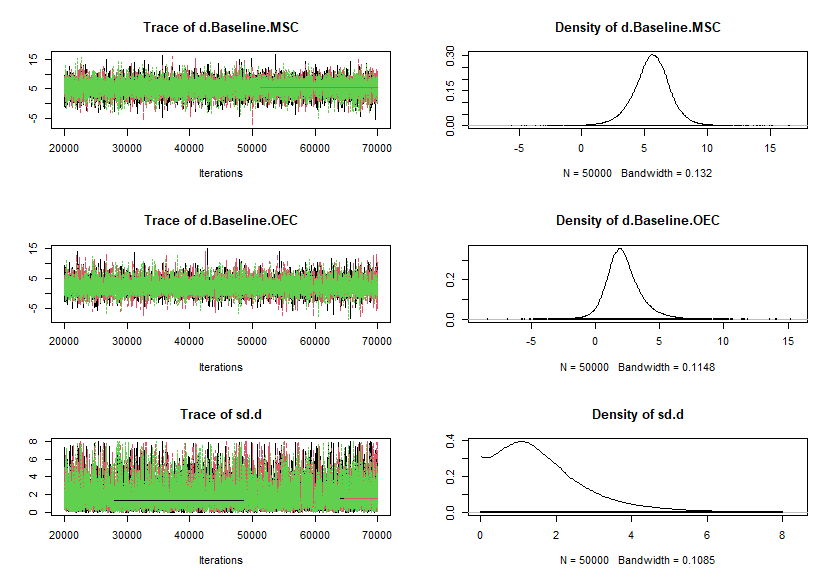


B. The iterative plots.


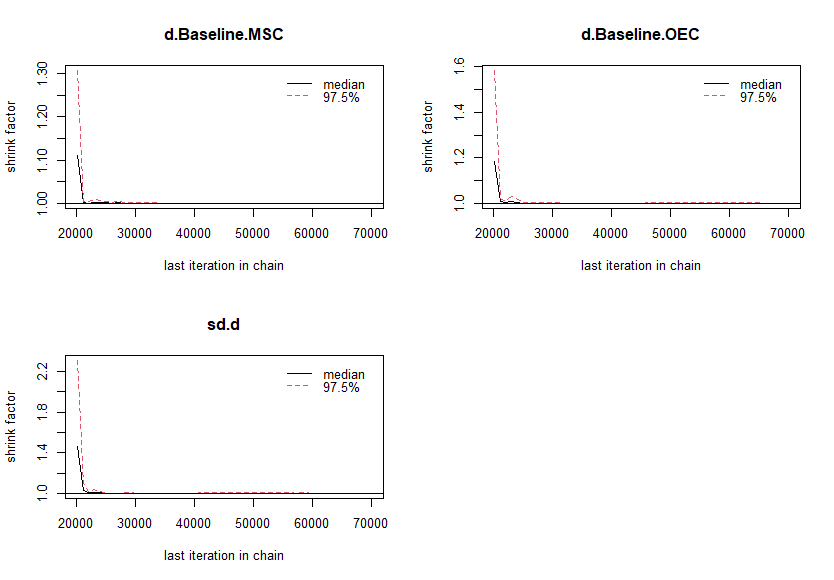

Supplement: Supplementary file 1 [file Data_Sheet_1.zip › Supplementary materials.docx]
